# Comparative Performance of Computer Simulation Models of Intrinsically Disordered Proteins at Different Levels of Coarse-Graining

**DOI:** 10.1021/acs.jcim.3c00113

**Published:** 2023-06-20

**Authors:** Eric Fagerberg, Marie Skepö

**Affiliations:** † Theoretical Chemistry, Lund University, POB 124, SE-221 00 Lund, Sweden; ‡ LINXS - Institute of Advanced Neutron and X-ray Science, Scheelevägen 19, SE-223 70 Lund, Sweden

## Abstract

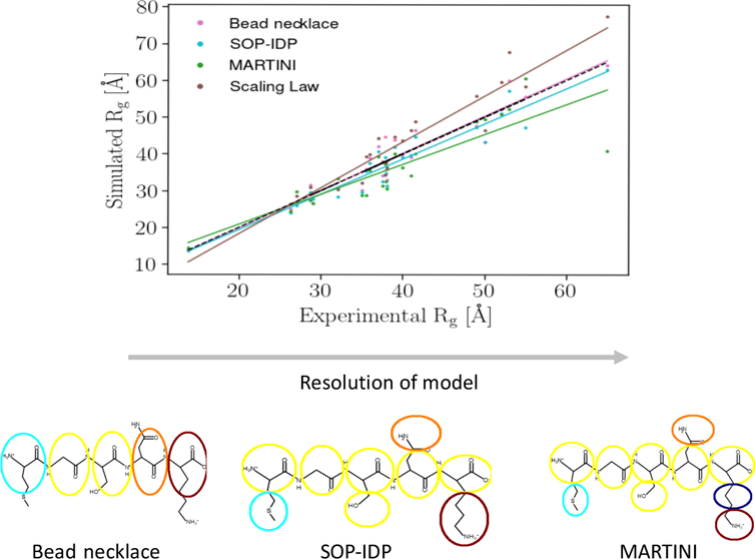

Coarse-graining is commonly used to decrease the computational cost of simulations. However, coarse-grained models are also considered to have lower transferability, with lower accuracy for systems outside the original scope of parametrization. Here, we benchmark a bead-necklace model and a modified Martini 2 model, both coarse-grained models, for a set of intrinsically disordered proteins, with the different models having different degrees of coarse-graining. The SOP-IDP model has earlier been used for this set of proteins; thus, those results are included in this study to compare how models with different levels of coarse-graining compare. The sometimes naive expectation of the least coarse-grained model performing best does not hold true for the experimental pool of proteins used here. Instead, it showed the least good agreement, indicating that one should not necessarily trust the otherwise intuitive notion of a more advanced model inherently being better in model choice.

## 1 Introduction

In order to decrease the computational cost of molecular simulations, coarse-graining (CG) can be an option, enabling simulations of both larger systems and slower processes. The idea is to average out finer details while keeping the degrees of freedom having the greatest impact on conformation and, thereby, obtaining a much simpler model.^1^ The potential energy function considers only an effective interaction between pseudoatoms, also denoted as beads, as in beads-on-a-string, or CG sites. A smoother free energy surface is attained, allowing for larger integration steps, which is why CG models may decrease the computational load by several orders of magnitude.^2,3^ Both the level of CG, i.e., the mapping of atoms or chemical fragments to pseudoatoms, and the effective energy potential vary widely depending on what properties are of interest or the main system of study. This could be mechanical properties,^4^ physical response to stimuli,^5^ protein folding,^6−10^ phase behavior of proteins,^11^ partitioning free energies,^12^ protein–protein interactions,^13−15^ structural properties,^16^ or aggregation,^17−19^ though models may find use or later be extended beyond the initial target properties. However, the simplifications introduced may also have drawbacks of lower transferability, having decreased accuracy for systems dissimilar to the parametrization source systems of the model.^20^ The choice of the model must be considered carefully.

Intrinsically disordered proteins (IDPs) lack a single specified structure, instead existing as an ensemble of wide-ranging conformers in solution.^21−23^ Despite this, they are biologically relevant, having roles in, for example, cell signaling, transcription, and regulation.^24−27^ In addition, they are abundant — estimates indicate 32% of residues in eukaryotic proteins to be disordered.^28^

Their flexibility, having an energy landscape of many, often shallow, minima rather than a single deep minimum,^29−31^ requires longer simulations in the absence of enhanced sampling techniques in order to properly explore all conformers of relevance. From that perspective, CG modeling is an excellent and advantageous fit for studying IDPs.

Recently, Baul et al.^32^ presented a two-bead per amino acid CG model named SOP-IDP, which was found to have, after parametrization, excellent agreement with small-angle X-ray scattering (SAXS) data for a curated set of IDPs. The same model was later used to find distinctions between Aβ40 and Aβ42 conformational ensembles.^33^ The SOP-IDP model was in part based on earlier two-bead models focused on folding,,^34^ but also in part based on a model by Cragnell et al.,^35^ more simple in terms of the number of parameters, size of the training set, and having a one-bead per amino acid mapping. Despite its simplicity and limited training set, it has been shown to have some transferability.^36^ Given the relationship between the models and the results of the SOP-IDP model, we here ask the question: How much more performance is gained by the more advanced, computationally expensive SOP-IDP model? Taking this somewhat provocative question, given that one should expect better performance from a more advanced model, one step further, we investigate how much more a yet more advanced model than the SOP-IDP model offers in terms of performance. The model of choice in this regard was the Martini 2 model,^37,38^ with the corrections of Stark et al.^39^ (hereafter referred to as “Martini Stark”). This model offers a four-bead per amino acid, thus being the most high-resolution model in this study.

This latter choice requires some motivation, as there are a few competitors. Primarily, there are more recent corrections made by Benayad et al.,^40^ which uses the same tuning strategy as Stark et al. of setting up a hyperparameter to scale interaction parameters. However, they find a different optimal value, in conflict with Stark et al. The latter tunes parameter for protein–protein interactions, as found by osmotic second virial coefficient experimental data, while the former tunes for liquid–liquid phase separation (LLPS) properties, e.g., droplet formation, excess transfer energy of a protein from/to solution and a droplet. Here, we take a cue from a study by Dannenhoffer-Lafage and Best,^41^ who found in the parametrization of a CG model a conflict between tuning for single-chain properties and LLPS properties and make an *a priori* hypothesis that there may be a similar situation here, choosing the parameters of Stark et al. as we are investigating single-chain properties.

Additionally, the newer version of Martini (version 3) should be considered. However, previous testing of a beta version of this model yielded less good results for an IDP.^42^ LLPS properties have, however, been semiquantitatively predicted,^43^ which would indicate that, if the “LLPS versus single chain properties conflict” is a general problem, this latest iteration of Martini is on the side of computing LLPS properties. Indeed, a recent paper published while this work was in progress showed Martini 3 to underestimate IDP dimensions for several IDPs.^44^

The performance measure employed here is the radius of gyration (*R*_g_), experimentally available through SAXS data, as found in the curated data set by Baul et al. This observable can be computed for all models considered. Still, it should be stressed that the performance of other properties may be different and, in some cases, cannot be reasonably considered for highly CG models.

## 2 Models and Methods

### 2.1 The Bead-Necklace Model

The bead-necklace model of Cragnell et al.,^35^ which traces its roots from studies of complexation between polyelectrolytes and macroions^45^ and surface adsorption of IDPs,^46,47^ considers each amino acid residue and the end terminals as a hard sphere with a radius of 2 Å. The hard spheres are linked with a harmonic potential, with the equilibrium distance set to 4.1 Å and a force constant of 0.4 N/m. The amino acid residues are differentiated by their charge, which is set to a fixed value of either +1, 0, or −1. To set these charges, a short titration simulation was run in the software Faunus,^48,49^ which uses the intrinsic p*K*_*a*_ values of Nozaki and Tanford.^50^ Electrostatics were treated through an extended Debye–Hückel potential where the volume of the particles was taken into account, and van der Waals interactions were considered through a soft short-range attractive potential, set to be the same for all amino acids, giving an attractive potential of 0.6 kT at the closest contact. Explicit counterions, one for each protein charge, were modeled as hard spheres of 2 Å radius and a +1 or −1 charge.

Simulations employing the bead-necklace model were performed using the software MOLSIM,^51^ in the canonical ensemble, thus, a constant number of particles, temperature, and volume, through the Monte Carlo Metropolis algorithm.^52^ A cubic simulation box was used, with a side length large enough to contain each protein’s contour length in the box. Periodic boundary conditions were applied in all directions, and the minimum image convention was employed to truncate long-ranged Coulomb interactions. Four trial moves were used: single-bead translation, pivot rotation, translation of the whole chain, and the slithering move.^53^ The probability of a single-bead trial move was 17 times more likely than the other moves, according to previous research that established this as a good ratio.^35,36^ For the starting structure, beads were randomly distributed in the box. For the initial equilibration of the system, 200,000 cycles of Monte Carlo simulation were used, followed by 1,000,000 cycles for the production run.

### 2.2 The Martini 2 Model with Modifications of Stark et al

Starting structures of the IDPs were obtained through the Robetta protein structure prediction service using the RoseTTAFold algorithm,^54^ with the exception of Histatin 5 (Hst5), which used a starting structure obtained from the final configuration of an atomistic molecular dynamics simulation previously published.^55^ The starting structures were converted to the Martini 2 resolution using the martinize.py^38^ script (version 2.4), with all residues set to have a secondary structure as a random coil. GROMACS version 2019.2,^56−59^ including associated tools, was used to further set up and perform the simulations. Initially, the box sizes were set to be the largest distances between the atoms in the initial structure, including a specified distance for the smaller proteins, of 20 Å, but increased to, initially, 40 Å for the larger proteins. More specifically, the Gromacs command *gmx editconf* was used, with input -d first set to 1.0; notice that Gromacs uses nm as the default length unit, which increases the box size so that the box edge is the specified distance away from any part of the protein in all directions. As initial results were gathered, box sizes were increased accordingly. The starting systems were energy minimized in a “dry” state, without solvent molecules, then solvated by GROMACS tool solvate. Solvent molecules were replaced by a number of ions (Na^+^, Cl^–^) via the GROMACS tool genion to achieve the specific salt content of each system. The system was thereafter energy minimized and equilibrated for 5 ns in the canonical ensemble, with the temperature held constant through the velocity-rescale algorithm^60^ using a temperature coupling parameter, referred to as tau-t in Gromacs, of 1 ps, grouping proteins, water, and ions separately,^61^ thus one coupling for each of these groups of particles. Another 5 ns equilibration in the isothermal–isobaric ensemble, e.g., constant pressure, constant temperature, and a constant number of particles, followed, using a Parrinello-Raman barostat,^62^ with a coupling constant of 12. Settings followed the recommendations of de Jong et al.^63^ The leapfrog algorithm^64^ with a 20 fs time step was used to integrate the equations of motion, a Verlet cutoff scheme, periodic boundary conditions in all directions, and a reaction field with a relative dielectric constant of 15 for electrostatics. Three replicates for each system were simulated. The simulation length for each system is detailed in SI, with a minimum of 5000 ns.

### 2.3 Scaling Law for IDPs

As the most minimal model possible, a scaling law for IDPs developed by Cragnell et al.^36^ is considered. The scaling law predicts *R*_g_ according to
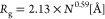
1where *N* is the number of amino acids in a protein. This scaling law considers no regard for the amino acid’s identity. In general, scaling laws with exponents roughly equal to 0.6 approximate self-avoiding random coil and exponents equal to 0.5 approximate random coils.^65^

### 2.4 The Data Set of IDPs

The original data set compiled by Baul et al. has been used as a basis. However, a few changes were necessary, as some of the cited studies did not provide full information on experimental conditions. For instance, in the case of Prothymosin α, where a study by Uversky et al.^66^ was originally cited, the amount of salt used in the experiment was not detailed, and information was not available. In the case of α-synuclein, the cited study did not state the salt concentration used. We, therefore, considered the study of Ahmed et al.^67^ instead, in which a lower *R*_g_ was found, which would compare more favorably with the result of the SOP-IDP model. The proteins KEIF^68^ and (Histatin 5)_2_^69^ were also considered for the bead-necklace model and the Martini Stark model.

### 2.5 Evaluation Metrics

To compute errors for each system and model, a signed percentage deviation is used, defined here as
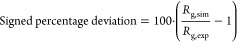
2where *R*_g,exp_ is the radius of gyration found in the experiment, and *R*_g,sim_ is the radius of gyration found from a given simulation model. To compare the models across all systems, a modified Pearson χ^2^ value was used
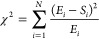
3where *E*_*i*_ is the experimental *R*_g_ for system *i*, and *S*_*i*_ is the simulation predicted *R*_g_ for system *i*. To further analyze the models in the form of biases, the fraction of charged residues, FCR = f_+_ + f_–_, with f_+_ being the fraction of positively charged residues and f_–_ being the fraction of negatively charged residues, the net charge per residue, NCPR = | f_+_ – f_–_|, hydrophobicity as per the Kyte-Doolittle scale,^70^ the proline content, and disorder score as per the PrDOS algorithm^71^ were considered. FCR, NCPR, and the hydrophobicity score were estimated via the CIDER Web server.^72^

## 3 Results and Discussion

### 3.1 Predictions of the Radius of Gyration of Model IDPs

The full comparison of predicted *R*_g_ using the models considered in this study with the experimental data is presented in Table 1.

**Table 1:** List of IDPs in Data Set of Baul et al., Along with Experimental *R*_g_ and Results from the Scaling Law^a^

Chain	N	*R*_g_, Exp	Scaling law	SOP-IDP	Bead-necklace	Martini Stark	Comment
Hst5	24	13.8 ± 0.04	13.9	13.6	13.8	14.4	
ACTR	71	26.3 ± UNK	26.3	24.0	25	24.3	Exp. *T* = 278 K
Nucleoporin	81	27 ± 4	28.5	25.9	26.8	29.7	Exp. *T* = 296 K
SH4-UD	85	29 ± 0.4	29.3	27.8	26.6	26.5	Exp. *T* = 277 K
Sic1	90	32.1 ± 0.8	30.3	28.4	30.7	33.4	Bead necklace from Cragnell et al.^35^
p53	93	28.7 ± UNK	30.9	29.5	31.5	27.4	Exp. *T* = 293 K
Proth. α	111	37.9 ± 0.9	34.3	39.1	72.3 (44.6)	37.2	Exp. *T* = 296 K, 0 (150) mM NaCl
ERM TADn	122	38.1 ± 0.7	36.3	31.4	33.0	30.9	Exp. *T* = 293 K
hNHE1	131	37.5 ± UNK	37.8	32.5	34.1	31.3	Exp. *T* = 278 K
Alp. Syn.	140	40.0 (35.5 ± 0.5)	39.3	35.4	36.0	28.8	*T* = 293, 200 mM NaCl
An16	185	50 ± 5	46.3	43.2	43.2	49.5	
Osteopontin	273	55 ± 1.7	58.3	47.2	55.6	60.5	
K19	99	35 ± 1	32.0	29.6	30.1	28.7	Exp. *T* = 288 K
K18	130	38 ± 3	37.6	34.8	35.8	32.5	Exp. *T* = 288 K
K17	143	36 ± 2	39.8	37.4	38.9	35.4	Exp. *T* = 288 K
K10	167	40 ± 1	43.6	39	39.9	36.3	Exp. *T* = 288 K
K27	171	37 ± 2	44.2	40.6	42	39.3	Exp. *T* = 288 K
K16	174	39 ± 3	44.7	41.8	43.8	40.0	Exp. *T* = 288 K
K25	185	41 ± 2	46.3	39.7	39.1	34.2	Exp. *T* = 288 K
K32	202	41.5 ± 3	48.8	44.7	46.4	40.1	Exp. *T* = 288 K
K23	254	49 ± 2	54.7	47.3	47.6	48.9	Exp. *T* = 288 K
K44	283	52 ± 2	59.6	51	52.2	50.8	Exp. *T* = 288 K
hTau23	352	53 ± 3	67.7	57.1	59.9	52.3	Exp. *T* = 288 K
hTau40	441	65 ± 3	77.4	62.9	64.1	40.8	Exp. *T* = 288 K, 140 mM NaCl

a Included are also simulations using the SOP-IDP model of Baul et al.,^32^ the bead-necklace model of Cragnell et al.,^35^ and the Martini Stark model with the modifications of Stark et al.^39^ All *R*_g_ given in Å. The number after ± indicates an error. UNK denotes the error to be unknown, i.e., not reported in the original literature.

For easier comparison across different IDPs, the percentage value is also calculated; see Table 2.

**Table 2:** Signed Percentage Deviation of Each Model from the Experiment in Terms of *R*_g_^a^

Chain	*N*	%, Scaling law	%, SOP-IDP	%, Bead-necklace	%, Martini Stark
Hst5	24	+1	–1	0	+5
ACTR	71	0	–9	–5	–7
Nucleoporin	81	+5	–4	–1	+10
SH4-UD	85	+1	–4	–8	–9
Sic1	90	–6	–12	–4	+4
p53	93	+8	+3	+10	–5
Proth. α	111	–10	+3	+18	–2
ERM TADn	122	–5	–18	–13	–20
hNHE1	131	+1	–13	–9	–17
Alp. Syn.	140	+11	0	+1	–19
An16	185	–7	–14	–14	–1
Osteopontin	273	+6	–14	+1	+10
K19	99	–8	–15	–14	–18
K18	130	–1	–8	–6	–14
K17	143	+11	+4	+8	–1
K10	167	+9	–3	0	–9
K27	171	+20	+10	+14	+6
K16	174	+15	+7	+12	+3
K25	185	+13	–3	–5	–17
K32	202	+18	+8	+12	–3
K23	254	+14	–3	–3	0
K44	283	+15	–2	0	–2
hTau23	352	+28	+8	+13	–1
hTau40	441	+19	–3	–1	–37

a Rounded to the closest integer.

From the above values, a “total” score is determined for each model according to a modified Pearson χ^2^, which was found to be 15.4 for the scaling law, 7.1 for the SOP-IDP model, 7.6 for the bead-necklace model, and 17.8 for the Martini Stark model. The difference found between SOP-IDP and the bead-necklace model should be considered small or nonexistent as a consistent 1% difference would, in this case, translate to a χ^2^ difference of about 0.2. In contrast, a consistent 2% difference yields a χ^2^ difference of about 0.8, particularly in the light of experiments also suffering from various levels of experimental uncertainty, detailed in Table 1. Thus, no overall performance increase is found, using the *R*_g_ as a performance metric. The Martini Stark model, on the other hand, has much lower performance than expected. Our expectations are based on the *a priori* assumption that a less coarse-grained model should have greater transferability and the expectation of the corrections of Stark et al. to have improved not just protein–protein interactions but also intrachain interactions. Probably, the corrections have had a positive impact, but seemingly not enough to be competitive with computationally less demanding models.

### 3.2 Deviations between Simulations and Experiments with Respect to IDP Properties

**Figure 1:**
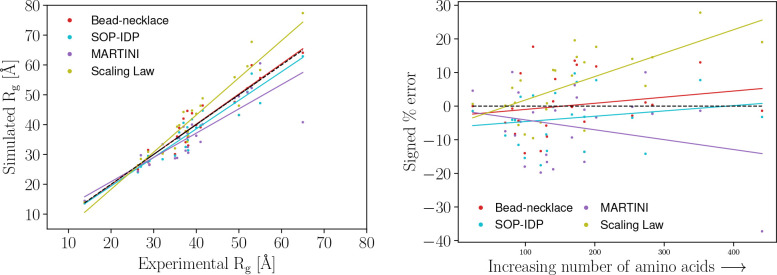
(Left) Simulated *R*_g_ plotted versus experimental *R*_g_ for each model. A perfect model would have all values distributed on the dashed black line. Trendlines for each model are also added, with *R*^2^ being: 0.96 for the scaling law, 0.95 for the SOP-IDP model, 0.95 for the bead-necklace model, and 0.86 for the Martini Stark model. (Right) The size of each protein in terms of the number of amino acids plotted against the signed percentage error. Trendlines have *R*^2^ of 0.67 for the scaling law, 0.19 for the SOP-IDP model, 0.19 for the bead-necklace model, and −0.25 for the Martini Stark model.

**Figure 2:**
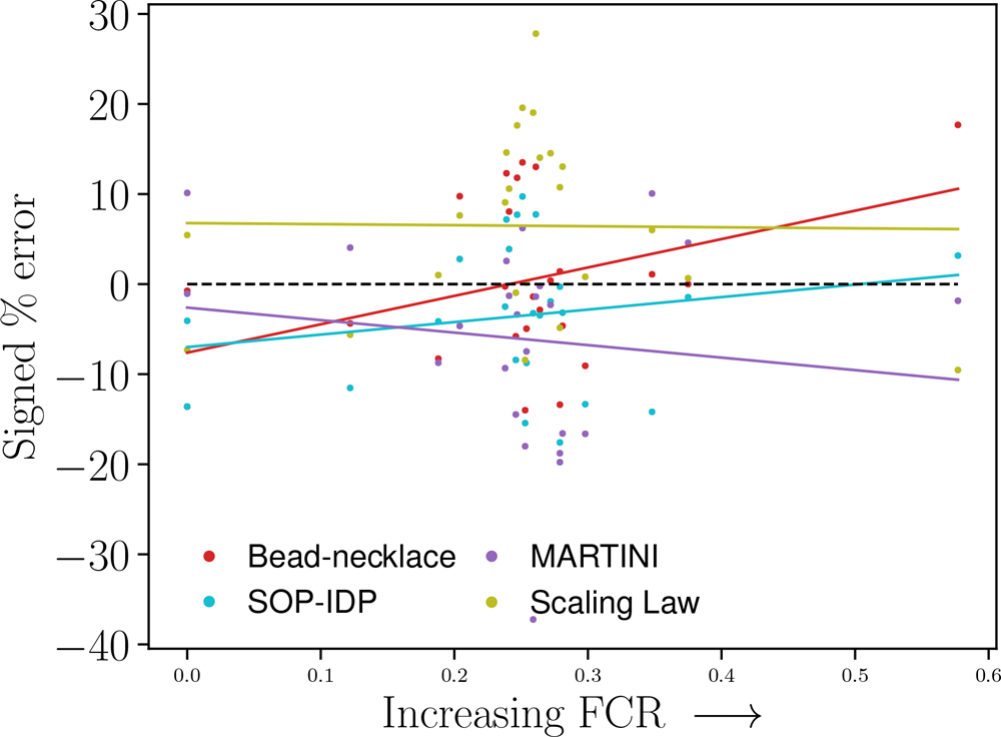
Signed percentage error of each model versus FCR. The black dashed line denotes a perfect match; i.e., each prediction has a signed percentage error of zero. Trendlines for each model included, with *R*^2^ being 0 for the scaling law, 0.19 for the SOP-IDP model, 0.38 for the bead-necklace model, and −0.14 for the Martini Stark model.

**Figure 3:**
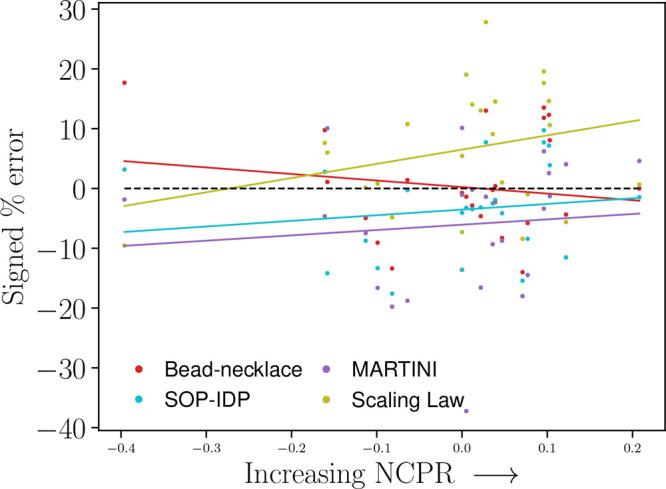
Signed percentage error of each model versus NCPR. The black dashed line denotes a perfect match; i.e., each prediction has a zero signed percentage error. Trendlines for each model included with *R*^2^ being 0.29 for the scaling law, 0.15 for the SOP-IDP model, −0.15 for the bead-necklace model, and 0.10 for the Martini Stark model.

**Figure 4:**
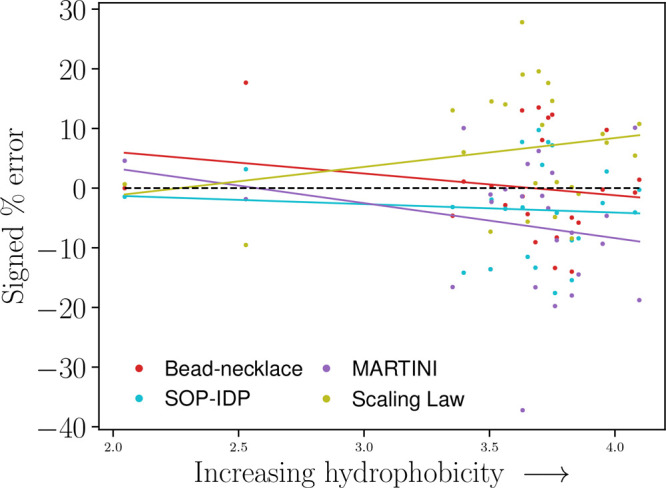
Signed percentage error of each model versus hydrophobicity. The black dashed line denotes a perfect match; i.e., each prediction has a signed percentage error of zero. Trendlines for each model included, with *R*^2^ being 0.22 for the scaling law, −0.08 for the SOP-IDP model, −0.18 for the bead-necklace model, and −0.24 for the Martini Stark model.

**Figure 5:**
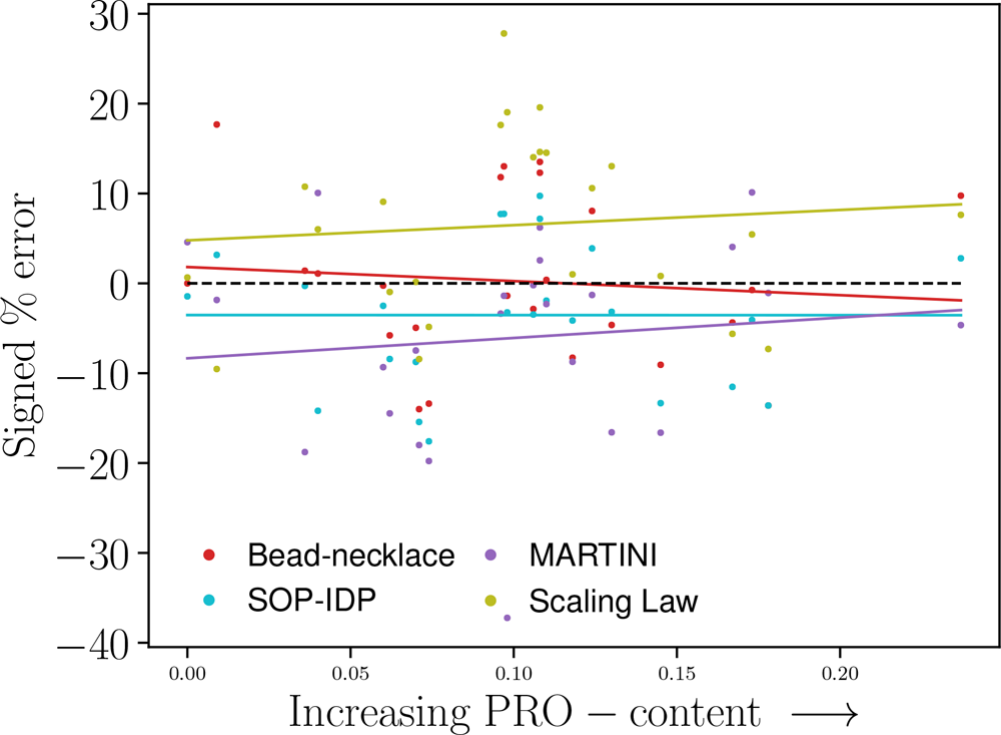
Signed percentage error of each model versus proline content. The black dashed line denotes a perfect match; i.e., each prediction has a signed percentage error of zero. Trendlines for each model included, with *R*^2^ being 0.09 for the scaling law, 0.00 for the SOP-IDP model, −0.09 for the bead-necklace model, and 0.11 for the Martini Stark model.

**Figure 6:**
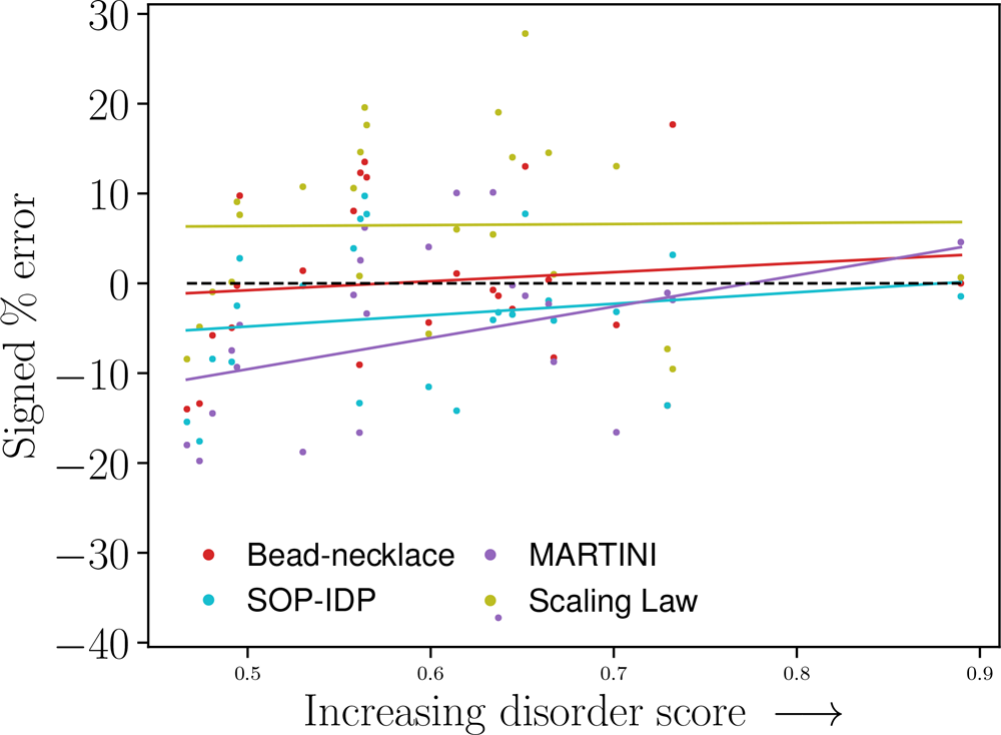
Signed percentage error of each model versus disorder score. The black dashed line denotes a perfect match; i.e., each prediction has a signed percentage error of zero. Trendlines for each model included, with *R*^2^ being 0.01 for the scaling law, 0.16 for the SOP-IDP model, 0.11 for the bead-necklace model, and 0.32 for the Martini Stark model.

One should consider whether any model performs better for any subclass of IDPs in the data set, thus if this particular data set may favor some models above others. Therefore, we also consider different metrics that could be a basis of bias, choosing the value of *R*_g_, number of amino acids, FCR, NCPR, hydrophobicity, proline content, and disorder score as computed by the PrDOS algorithm.^71^ The data are plotted as a function of each of these metrics for each model in Figures 1–6. The specific value for each metric for each individual protein is found in SI Table S1.

#### 3.2.1 Model Deviations as a Function of IDP length

Investigating the error as a function of size (first defining size as the experimentally found *R*_g_), we plot the signed percentage error for each model against the protein size in Figure 1 (left). Seemingly, the SOP-IDP and Martini Stark models tend to underestimate *R*_g_ as *R*_g_ becomes larger. In contrast, the scaling law has a tendency to overestimate *R*_g_ for large *R*_g_. Defining size as the number of amino acids (Figure 1, right), the overall trends from using *R*_g_ remain except for the SOP-IDP model. In particular, the trendline *R*^2^ for the scaling law is considerably higher than for the other models, indicating that this model’s tendency to overestimate *R*_g_ for large proteins is strong. The overprediction could be due to an overly large exponent (a current setting of roughly 0.6 indicates self-avoiding random coil) or a too-large prefactor. By fitting the experimental *R*_g_ data to an equation of the same form as eq 1, a fit of
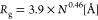
4with an *R*^2^ of 0.93 was found. The exponent found in eq 1 is closer to 0.5, indicating that the data set resembles random coils better than self-avoiding random walks.

On a more speculative note on the performance of the models as a function of the number of residues in the protein chain, the chain would have the possibility of self-entangling for longer protein chains. Very coarse-grained models, such as the bead-necklace model, should experience less self-entanglement, as less resolved mappings would be less easily entangled. The reverse would tentatively also be true; high-resolution models can self-entangle to a higher degree. Thus, the behavior of the Martini Stark model can be viewed from a polymer theory perspective: with longer chains, there are possibly exaggerated self-entanglement effects. Such an issue would ultimately be related to the possible convergence problem, discussed further below. This effect would speculatively contribute to the observation in Figure 1 (left) of Martini Stark having a slight tendency of predicting too compact structures.

#### 3.2.2 Model Deviations as a Function of IDP Charge Properties

Regarding FCR, the bead-necklace model seems biased and less performing with increasing FCR. This would be somewhat counterintuitive, given that the model was parametrized with Hst5, having the second largest FCR of the entire data set. However, this indication may be fairly influenced by the larger deviation in the prediction of the bead-necklace model with Prothymosin α, having the largest FCR in the data set.

Using net charge per residue (NCPR), the scaling law indicates a weak tendency to overestimate *R*_g_ with increasing net charge per residue, though it is a very weak trend (Figure 3).

#### 3.2.3 Model Deviations as a Function of IDP Hydrophobicity

A trendline showing an increasing negative percentage error with increasing hydrophobicity is indicated for the Martini Stark model, while the reverse is true for the scaling law. Both of these trends are very weak. The bead-necklace model was expected to perform less with increasing hydrophobicity, given that it only differentiates proteins by charged residues. Still, the observed bias for this data set is similar to that of the Martini Stark model.

#### 3.2.4 Model Deviations as a Function of Proline Content

As seen in Figure 5 and the *R*^2^ values (given in the figure caption), no strong bias is found regarding proline content. Also, previous research by Cragnell et al.^36^ found, in particular, the bead-necklace model to be able to predict *R*_g_ for IDPs containing proline, though considering a limited data set. Our results confirm these early reports.

#### 3.2.5 Model Deviations as a Function of IDP PrDOS Disorder Score

As seen in Figure 6, the Martini Stark model shows less performance with systems with a low PrDOS disorder score; note that the lowest disorder score was 0.47. This was unexpected, as the improvements made by Stark et al. were found using globular proteins such as hen egg white lysozyme, PDB code 1HEL, with a disorder score of 0.18, and bovine chymotrypsinogen, PDB code 2CGA, with a disorder score of 0.13. The opposite trend would have been expected.

As shown by most *R*^2^ values, no strong bias is found. Indeed, one could easily confound different variables, even if the correlations were stronger. A larger and more diverse (in terms of sampling different properties) data set could reach different findings.

### 3.3 Extension with KEIF and (Histatin 5)_2_

Extending the data set slightly, we also consider how the bead-necklace and Martini Stark models compare with the short peptides KEIF and (Histatin 5)_2_. The latter, in particular, has been found to be somewhat challenging to simulate even with a fully atomistic model, predicting *R*_g_ to be 23.3 Å while SAXS data indicate *R*_g_ to be 18.7 Å.^69^ As well, also the bead-necklace model has previously predicted a too large *R*_g_ of 21.0 Å. Here, it is found that the Martini Stark model predicts *R*_g_ to be 23.6 Å, in line with the atomistic simulation but deviating compared to the experimental value. The bead-necklace model seems to be the best predictor, albeit still erroneous.

KEIF is, on the other hand, predicted by both the bead-necklace model and the Martini Stark model to have *R*_g_ of 16.0 Å, smaller than the experimental value of 17.6 Å,^68^ but in line with previous atomistic simulation (16.4 Å). Thus, all models considered here, even referenced atomistic models, differ very little regarding predicted *R*_g_ for KEIF. However, the scaling law prediction, 16.8 Å, is actually the best predictor, which is fairly curious given that the scaling law overall is the worst predictor. Speculatively, the small size of KEIF may play a part in this, as well as (Histatin 5)_2_. The peptide closest in size to these is Hst5, which was overall well predicted, but Hst5 was also part of the training set for both the scaling law and the bead-necklace model, which is why a more strict evaluation should leave Hst5 out. This leaves the overall conclusion that these smaller peptides are difficult to model.

### 3.4 The Issue of Convergence

A CG model should generally have a smoother energy landscape than an atomistic model. This would influence the ease at which a simulation reaches convergence.^2^ With decreasing CG among the bench-marked models here, in principle, the bead-necklace model should have the least issue with convergence, the SOP-IDP model having slightly more convergence time, and the Martini Stark model the most convergence issues, in particular for the very large systems. Indeed, for some systems, plots of *R*_g_ versus simulation time indicate replicates being trapped in local minima, see SI, where notable examples are SH4-UD, Prothymosin α, and K44. However, for these examples, the average *R*_g_ values across all replicates are fairly similar to experimental values; hence, sampling may still be considered adequate, probably as replicates sample different local minima. The system with the least experimental agreement in the Martini Stark modeling, hTau40, shows two replicates approaching a low value of about 40 Å, with the third also trending toward a lower than expected value. This is particularly unexpected since the RoseTTAFold initial structure aligns well with the experimental *R*_g_. When a smaller simulation box was used, also here two of three replicates trended toward an *R*_g_ of about 40 Å (data not shown), indicating the tendency toward this structural region to be strong. Thus, the result is considered to be accurate. Investigating this behavior more closely by considering the contact maps (found in Supporting Information), interactions with several residues in the range 50–100 themselves and with residues around 250 and 370 are underestimated in replicates 2 and 3, which is somewhat counterintuitive. More contracted structures would be expected to have more intrachain interactions, not the opposite. Several differences in local contacts are also observed.

It is noted that the hTau40 system contributes greatly to the χ^2^ of the Martini Stark model. Exclusion of this system changes the χ^2^ of all the models to 13.0 for the scaling law, 7.1 for the SOP-IDP model, 7.6 for the bead-necklace model, and 8.8 for the Martini Stark model, showing better performance comparability with the other models. But even with this cherry-picking, the Martini Stark model is least consistent with the experimental data pool of the three simulation models tested here.

### 3.5 Observations of Systems with Large Prediction/Experiment Discrepancy Using Martini Stark

Among other less well-predicted systems using the Martini Stark model, defined as a percentage deviation greater than 15, it is found that ERM TADn has fairly good convergence (see Supporting Information), with all replicates showing similar behavior, in particular, all intrachain interactions are fairly local, with some longer ranged interactions between residues 80–90. Regarding hNHE1, replicate 1 has a much lower *R*_g_ for most of the simulation time until the end, where it achieves similar *R*_g_ as the other two replicates. The contact maps indicate that replicate 1 has many long-range interactions while the others do not. Extending the simulation further may increase agreement with experimental data, provided replicates sample similar to the last part of the simulation. However, given that the other replicates still display too low *R*_g_ for hNHE1, an increased simulation time would only improve *R*_g_ to a limited extent. α-Synuclein has fair convergence, with intrachain interactions being mainly local, with slightly longer-range interactions in residue regions 10–30 and 80–100. K19 has only local intrachain interactions, except in the N-terminal region, up to about residue 15. Lastly, K25 has notable interaction centers around residues 70 and 100 and a weak interaction center at about residue 125.

### 3.6 Distribution of Counterions

The bead-necklace model includes explicit counterions in the simulations, one counterion for each charge. The radial distribution function (RDF) of the counterion distance to the protein chain is shown in Supporting Information. The results are as expected: proteins with a net positive charge attract negative counterions and repel positive ones and vice versa for proteins with a net negative charge. The degree of this behavior would depend on the distribution of the charges along the protein chain and the radially averaged structure of the protein. All simulations have a minimum of two counterions in the system due to explicit modeling of end terminals, which are charged in the system. Having very few counterions in the simulation box would affect the convergence of the RDF. This is particularly clear in the case of An16 (Figure S63). Convergence of counterion RDF does seemingly not have a large effect on *R*_g_ convergence. In particular, An16 has fairly good convergence and slight bias toward smaller *R*_g_ in the later part of the simulation.

### 3.7 Remarks on the Models

In this study, we only use three models of varying CG level to gain insight into the added performance of more fine-grained models. Additional models would have been useful, as these models might not be the best representative of each CG level. At the same time, it is recognized that the CG level and the number of parameters matter.

To some extent, the SOP-IDP model has an advantage in this data set, as six IDPs (Histatin 5, ACTR, hNHE1, K32, K23, and hTau40) were part of the training set for SOP-IDP. In this context, it is interesting that hNHE1, one of the training set IDPs, is one of the proteins most problematic to simulate with the SOP-IDP model. This could indicate that SOP-IDP parametrization did not overfit the initial training set.

The most advanced model, Martini Stark, was found to have the least good agreement with experimental data. However, this model has a much larger degree of generality since it can also be used to model globular proteins, for which the other models are unfit. Therefore, it may not be surprising that the IDP-specific models outperform the Martini Stark model for an IDP data set.

During the setup of the Martini Stark model, one needs to define the secondary structure for each amino acid. Here, we have let the secondary structure be a coil for the entirety of the IDPs considered. Still, a possibility is to use a prediction tool of secondary structure and set the secondary structure to that predicted. Even if this may seem contrary to the flexibility of IDPs, it can be possible that any segment of an IDP samples a specific secondary structure to such a great extent while still interconverting to other structures. Hence, it is reasonable to set that segment to have a secondary structure throughout the simulation. However, this would be heavily dependent on the accuracy of the method for determining secondary structure, which is why we here avoid such a procedure while noting that it might be possible to achieve higher performance with such a procedure.

## 4 Conclusions

CG modeling is advantageous because it allows us to simulate complex and dense systems and to achieve an understanding of the underlying physics, the former being next to impossible with atomistic simulations with current computation technology. Here, we present an initial study using a set of 24 IDPs to determine to what extent CG models actually differ with respect to the level of detail.

This initial study concludes that a more fine-grained model may not have more predictive power than a simpler one. No strong biases for either model for a selected number of metrics were found, which would otherwise have motivated the preference of one model for a subclass of IDPs. Future studies should involve a larger pool of IDPs, including increased length of the primary sequence and more diversity in different IDP properties.

## Data Availability

The software package MOLSIM is available at https://github.com/joakimstenhammar/molsim. Gromacs is available at https://manual.gromacs.org. Simulation setup files can be found at https://zenodo.org/record/7523635.

## Supporting Information Available

Table of properties for the proteins. Table containing the starting *R*_g_ from the RoseTTAFold model and *R*_g_ obtained for each replicate for the Martini Stark model, figures of the running average of *R*_g_ evolution over time for the Martini Stark simulations, for convergence consideration. Computed contact maps for Martini Stark model and the radial distribution function of counterions versus protein chain for the bead-necklace model. Graphs of average *R*_g_ for each macrostep for the bead-necklace simulations for convergence consideration are included. (PDF)

## References

[ref1] Levitt M.; Warshel A. Computer simulation of protein folding. Nature 1975, 253, 694–698. 10.1038/253694a0. 1167625

[ref2] Pak A.; Voth G. Advances in coarse-grained modeling of macromolecular complexes. Curr. Opin. Struct. Biol. 2018, 52, 119 10.1016/j.sbi.2018.11.005. 30508766 PMC6296860

[ref3] Ingólfsson H. I.; Lopez C. A.; Uusitalo J. J.; de Jong D. H.; Gopal S. M.; Periole X.; Marrink S. J. The power of coarse graining in biomolecular simulations. WIREs Computational Molecular Science 2014, 4, 225–248. 10.1002/wcms.1169. 25309628 PMC4171755

[ref4] Pasi M.; Lavery R.; Ceres N. PaLaCe: A Coarse-Grain Protein Model for Studying Mechanical Properties. J. Chem. Theory Comput. 2013, 9, 785–793. 10.1021/ct3007925. 26589071

[ref5] Yeo J.; Huang W.; Tarakanova A.; Zhang Y.-W.; Kaplan D. L.; Buehler M. J. Unraveling the molecular mechanisms of thermo-responsive properties of silk-elastin-like proteins by integrating multiscale modeling and experiment. J. Mater. Chem. B 2018, 6, 3727–3734. 10.1039/C8TB00819A. 30467524 PMC6241539

[ref6] Bereau T.; Deserno M. Generic coarse-grained model for protein folding and aggregation. J. Chem. Phys. 2009, 130, 235106 10.1063/1.3152842. 19548767 PMC3910140

[ref7] Coluzza I. Transferable Coarse-Grained Potential for De Novo Protein Folding and Design. PLoS One 2014, 9, e112852 10.1371/journal.pone.0112852. 25436908 PMC4249799

[ref8] Das P.; Matysiak S.; Clementi C. Balancing energy and entropy: A minimalist model for the characterization of protein folding landscapes. Proc. Natl. Acad. Sci. U. S. A. 2005, 102, 10141–10146. 10.1073/pnas.0409471102. 16006532 PMC1177359

[ref9] Wolff K.; Vendruscolo M.; Porto M. Coarse-grained model for protein folding based on structural profiles. Phys. Rev. E 2011, 84, 041934 10.1103/PhysRevE.84.041934. 22181202

[ref10] Maupetit J.; Tuffery P.; Derreumaux P. A coarse-grained protein force field for folding and structure prediction. Proteins: Struct., Funct., Bioinf. 2007, 69, 394–408. 10.1002/prot.21505. 17600832

[ref11] Dignon G. L.; Zheng W.; Kim Y. C.; Best R. B.; Mittal J. Sequence determinants of protein phase behavior from a coarse-grained model. PLOS Computational Biology 2018, 14, e1005941 10.1371/journal.pcbi.1005941. 29364893 PMC5798848

[ref12] Marrink S. J.; Risselada H. J.; Yefimov S.; Tieleman D. P.; de Vries A. H. The MARTINI Force Field: Coarse Grained Model for Biomolecular Simulations. J. Phys. Chem. B 2007, 111, 7812–7824. 10.1021/jp071097f. 17569554

[ref13] Andrews C. T.; Elcock A. H. COFFDROP: A Coarse-Grained Nonbonded Force Field for Proteins Derived from All-Atom Explicit-Solvent Molecular Dynamics Simulations of Amino Acids. J. Chem. Theory Comput. 2014, 10, 5178–5194. 10.1021/ct5006328. 25400526 PMC4230375

[ref14] Frembgen-Kesner T.; Andrews C. T.; Li S.; Ngo N. A.; Shubert S. A.; Jain A.; Olayiwola O. J.; Weishaar M. R.; Elcock A. H. Parametrization of Backbone Flexibility in a Coarse-Grained Force Field for Proteins (COFFDROP) Derived from All-Atom Explicit-Solvent Molecular Dynamics Simulations of All Possible Two-Residue Peptides. J. Chem. Theory Comput. 2015, 11, 2341–2354. 10.1021/acs.jctc.5b00038. 26574429 PMC4658516

[ref15] Basdevant N.; Borgis D.; Ha-Duong T. Modeling Protein–Protein Recognition in Solution Using the Coarse-Grained Force Field SCORPION. J. Chem. Theory Comput. 2013, 9, 803–813. 10.1021/ct300943w. 26589072

[ref16] Ghavami A.; van der Giessen E.; Onck P. R. Coarse-Grained Potentials for Local Interactions in Unfolded Proteins. ournal of Chemical Theory and Computation 2013, 9, 432–440. 10.1021/ct300684j. 26589045

[ref17] Ruff K. M.; Harmon T. S.; Pappu R. V. CAMELOT: A machine learning approach for coarse-grained simulations of aggregation of block-copolymeric protein sequences. J. Chem. Phys. 2015, 143, 243123 10.1063/1.4935066. 26723608 PMC4644154

[ref18] Bellesia G.; Shea J.-E. Self-assembly of β -sheet forming peptides into chiral fibrillar aggregates. J. Chem. Phys. 2007, 126, 245104 10.1063/1.2739547. 17614592

[ref19] Morriss-Andrews A.; Brown F. L. H.; Shea J.-E. A Coarse-Grained Model for Peptide Aggregation on a Membrane Surface. J. Phys. Chem. B 2014, 118, 8420–8432. 10.1021/jp502871m. 24791936

[ref20] Kar P.; Feig M. Biomolecular Modelling and Simulations; Advances in Protein Chemistry and Structural Biology series; Karabencheva-Christova T., Ed.; Academic Press, 2014; Vol. 96; pp 143–180. 10.1016/bs.apcsb.2014.06.005 PMC5366245 25443957

[ref21] Dyson H. J.; Wright P. E. Intrinsically unstructured proteins and their functions. Nat. Rev. Mol. Cell Biol. 2005, 6, 197–208. 10.1038/nrm1589. 15738986

[ref22] Uversky V. N. Intrinsically disordered proteins and their “mysterious”(meta) physics. Frontiers in Physics 2019, 7, 10 10.3389/fphy.2019.00010.

[ref23] Radivojac P.; Obradovic Z.; Smith D. K.; Zhu G.; Vucetic S.; Brown C. J.; Lawson J. D.; Dunker A. K. Protein flexibility and intrinsic disorder. Protein Sci. 2004, 13, 71–80. 10.1110/ps.03128904. 14691223 PMC2286519

[ref24] Uversky V. N.; Dunker A. K. Understanding protein non-folding. Biochim. Biophys. Acta, Proteins Proteomics 2010, 1804, 1231–1264. 10.1016/j.bbapap.2010.01.017. PMC2882790 20117254

[ref25] Tompa P. Intrinsically disordered proteins: a 10-year recap. Trends Biochem. Sci. 2012, 37, 509–516. 10.1016/j.tibs.2012.08.004. 22989858

[ref26] Iadanza M. G.; Jackson M. P.; Hewitt E. W.; Ranson N. A.; Radford S. E. A new era for understanding amyloid structures and disease. Nat. Rev. Mol. Cell Biol. 2018, 19, 755 10.1038/s41580-018-0060-8. 30237470 PMC7617691

[ref27] Karamanos T. K.; Jackson M. P.; Calabrese A. N.; Goodchild S. C.; Cawood E. E.; Thompson G. S.; Kalverda A. P.; Hewitt E. W.; Radford S. E. Structural mapping of oligomeric intermediates in an amyloid assembly pathway. eLife 2019, 8, e46574 10.7554/eLife.46574. 31552823 PMC6783270

[ref28] Basile W.; Salvatore M.; Bassot C.; Elofsson A. Why do eukaryotic proteins contain more intrinsically disordered regions?. PLOS Computational Biology 2019, 15, e1007186 10.1371/journal.pcbi.1007186. 31329574 PMC6675126

[ref29] Chebaro Y.; Ballard A. J.; Chakraborty D.; Wales D. J. Intrinsically Disordered Energy Landscapes. Sci. Rep. 2015, 5, 10386 10.1038/srep10386. 25999294 PMC4441119

[ref30] Kulkarni P.; Leite V. B. P.; Roy S.; Bhattacharyya S.; Mohanty A.; Achuthan S.; Singh D.; Appadurai R.; Rangarajan G.; Weninger K.; Orban J.; Srivastava A.; Jolly M. K.; Onuchic J. N.; Uversky V. N.; Salgia R. Intrinsically disordered proteins: Ensembles at the limits of Anfinsen’s dogma. Biophysics Reviews 2022, 3, 011306 10.1063/5.0080512. PMC10903413 38505224

[ref31] Granata D.; Baftizadeh F.; Habchi J.; Galvagnion C.; De Simone A.; Camilloni C.; Laio A.; Vendruscolo M. The inverted free energy landscape of an intrinsically disordered peptide by simulations and experiments. Sci. Rep. 2015, 5, 15449 10.1038/srep15449. 26498066 PMC4620491

[ref32] Baul U.; Chakraborty D.; Mugnai M. L.; Straub J. E.; Thirumalai D. Sequence Effects on Size, Shape, and Structural Heterogeneity in Intrinsically Disordered Proteins. J. Phys. Chem. B 2019, 123, 3462–3474. 10.1021/acs.jpcb.9b02575. 30913885 PMC6920032

[ref33] Chakraborty D.; Straub J. E.; Thirumalai D. Differences in the free energies between the excited states of Aβ40 and Aβ42 monomers encode their aggregation propensities. Proc. Natl. Acad. Sci. U. S. A. 2020, 117, 19926–19937. 10.1073/pnas.2002570117. 32732434 PMC7443889

[ref34] Hyeon C.; Dima R. I.; Thirumalai D. Pathways and Kinetic Barriers in Mechanical Unfolding and Refolding of RNA and Proteins. Structure 2006, 14, 1633–1645. 10.1016/j.str.2006.09.002. 17098189

[ref35] Cragnell C.; Durand D.; Cabane B.; Skepö M. Coarse-grained modeling of the intrinsically disordered protein Histatin 5 in solution: Monte Carlo simulations in combination with SAXS. Proteins: Struct., Funct., Bioinf. 2016, 84, 777–791. 10.1002/prot.25025. 26914439

[ref36] Cragnell C.; Rieloff E.; Skepö M. Utilizing Coarse-Grained Modeling and Monte Carlo Simulations to Evaluate the Conformational Ensemble of Intrinsically Disordered Proteins and Regions. J. Mol. Biol. 2018, 430, 2478–2492. 10.1016/j.jmb.2018.03.006. 29573987

[ref37] Monticelli L.; Kandasamy S. K.; Periole X.; Larson R. G.; Tieleman D. P.; Marrink S. J. The MARTINI Coarse-Grained Force Field: Extension to Proteins. J. Chem. Theory Comput. 2008, 4, 819–834. 10.1021/ct700324x. 26621095

[ref38] de Jong D. H.; Singh G.; Bennett W. F. D.; Arnarez C.; Wassenaar T. A.; Schäfer L. V.; Periole X.; Tieleman D. P.; Marrink S. J. Improved Parameters for the Martini Coarse-Grained Protein Force Field. J. Chem. Theory Comput. 2013, 9, 687–697. 10.1021/ct300646g. 26589065

[ref39] Stark A. C.; Andrews C. T.; Elcock A. H. Toward Optimized Potential Functions for Protein–Protein Interactions in Aqueous Solutions: Osmotic Second Virial Coefficient Calculations Using the MARTINI Coarse-Grained Force Field. J. Chem. Theory Comput. 2013, 9, 4176–4185. 10.1021/ct400008p. PMC3819042 24223529

[ref40] Benayad Z.; von Bülow S.; Stelzl L. S.; Hummer G. Simulation of FUS Protein Condensates with an Adapted Coarse-Grained Model. J. Chem. Theory Comput. 2021, 17, 525–537. 10.1021/acs.jctc.0c01064. 33307683 PMC7872324

[ref41] Dannenhoffer-Lafage T.; Best R. B. A Data-Driven Hydrophobicity Scale for Predicting Liquid–Liquid Phase Separation of Proteins. J. Phys. Chem. B 2021, 125, 4046–4056. 10.1021/acs.jpcb.0c11479. 33876938 PMC12442143

[ref42] Fagerberg E.; Lenton S.; Skepö M. Evaluating Models of Varying Complexity of Crowded Intrinsically Disordered Protein Solutions Against SAXS. J. Chem. Theory Comput. 2019, 15, 6968–6983. 10.1021/acs.jctc.9b00723. 31714774

[ref43] Tsanai M.; Frederix P. W. J. M.; Schroer C. F. E.; Souza P. C. T.; Marrink S. J. Coacervate formation studied by explicit solvent coarse-grain molecular dynamics with the Martini model. Chemical Science 2021, 12, 8521–8530. 10.1039/D1SC00374G. 34221333 PMC8221187

[ref44] Thomasen F. E.; Pesce F.; Roesgaard M. A.; Tesei G.; Lindorff-Larsen K. Improving Martini 3 for Disordered and Multidomain Proteins. J. Chem. Theory Comput. 2022, 18, 2033–2041. 10.1021/acs.jctc.1c01042. 35377637

[ref45] Jonsson M.; Linse P. Polyelectrolyte–macroion complexation. I. Effect of linear charge density, chain length, and macroion charge. J. Chem. Phys. 2001, 115, 3406–3418. 10.1063/1.1385792.

[ref46] Skepö M.; Linse P.; Arnebrant T. Coarse-Grained Modeling of Proline Rich Protein 1 (PRP-1) in Bulk Solution and Adsorbed to a Negatively Charged Surface. J. Phys. Chem. B 2006, 110, 12141–12148. 10.1021/jp056033o. 16800528

[ref47] Skepö M. Model simulations of the adsorption of statherin to solid surfaces: Effects of surface charge and hydrophobicity. J. Chem. Phys. 2008, 129, 185101 10.1063/1.3002317. 19045429

[ref48] Lund M.; Trulsson M.; Persson B. Faunus: An object oriented framework for molecular simulation. Source Code for Biology and Medicine 2008, 3, 1 10.1186/1751-0473-3-1. 18241331 PMC2266748

[ref49] Stenqvist B.; Thuresson A.; Kurut A.; Vácha R.; Lund M. Faunus – a flexible framework for Monte Carlo simulation. Mol. Simul. 2013, 39, 1233–1239. 10.1080/08927022.2013.828207.

[ref50] Nozaki Y.; Tanford C. Enzyme Structure; Methods in Enzymology; Academic Press, 1967; Vol. 11; pp 715–734.

[ref51] Jurij R.; Per L. MOLSIM: A modular molecular simulation software. J. Comput. Chem. 2015, 36, 1259–1274. 10.1002/jcc.23919. 25994597 PMC5033024

[ref52] Allen M. P.; Tildesley D. H. Computer Simulation of Liquids, Second ed.; Oxford University Press, 2017.

[ref53] Binder K., Ed.; Monte Carlo and Molecular Dynamics Simulations in Polymer Science.; Oxford University Press, 1995.

[ref54] Baek M.; DiMaio F.; Anishchenko I.; Dauparas J.; Ovchinnikov S.; Lee G. R.; Wang J.; Cong Q.; Kinch L. N.; Schaeffer R. D.; Millán C.; Park H.; Adams C.; Glassman C. R.; DeGiovanni A.; Pereira J. H.; Rodrigues A. V.; van Dijk A. A.; Ebrecht A. C.; Opperman D. J.; Sagmeister T.; Buhlheller C.; Pavkov-Keller T.; Rathinaswamy M. K.; Dalwadi U.; Yip C. K.; Burke J. E.; Garcia K. C.; Grishin N. V.; Adams P. D.; Read R. J.; Baker D. Accurate prediction of protein structures and interactions using a three-track neural network. Science 2021, 373, 871–876. 10.1126/science.abj8754. 34282049 PMC7612213

[ref55] Henriques J.; Skepö M. Molecular Dynamics Simulations of Intrinsically Disordered Proteins: On the Accuracy of the TIP4P-D Water Model and the Representativeness of Protein Disorder Models. J. Chem. Theory Comput. 2016, 12, 3407–3415. 10.1021/acs.jctc.6b00429. 27243806

[ref56] Berendsen H.; van der Spoel D.; van Drunen R. GROMACS: A message-passing parallel molecular dynamics implementation. Comput. Phys. Commun. 1995, 91, 43–56. 10.1016/0010-4655(95)00042-E.

[ref57] Lindahl E.; Hess B.; van der Spoel D. GROMACS 3.0: a package for molecular simulation and trajectory analysis. J. Mol. Model. 2001, 7, 306–317. 10.1007/s008940100045.

[ref58] van Der Spoel D.; Lindahl E.; Hess B.; Groenhof G.; Mark A. E.; Berendsen H. J. C. GROMACS: Fast, flexible, and free. J. Comput. Chem. 2005, 26, 1701–1718. 10.1002/jcc.20291. 16211538

[ref59] Hess B.; Kutzner C.; van der Spoel D.; Lindahl E. GROMACS 4: Algorithms for Highly Efficient, Load-Balanced, and Scalable Molecular Simulation. J. Chem. Theory Comput. 2008, 4, 435–447. 10.1021/ct700301q. 26620784

[ref60] Bussi G.; Donadio D.; Parrinello M. Canonical sampling through velocity rescaling. J. Chem. Phys. 2007, 126, 014101 10.1063/1.2408420. 17212484

[ref61] Abraham M.; van der Spoel D.; Lindahl E.; Hess B.; GROMACS User Manual version 2019. GROMACS development team, 2019; Chapter 5.4, pp 317–322. http://www.gromacs.org.

[ref62] Parrinello M.; Rahman A. Polymorphic transitions in single crystals: A new molecular dynamics method. J. Appl. Phys. 1981, 52, 7182–7190. 10.1063/1.328693.

[ref63] de Jong D. H.; Baoukina S.; Ingólfsson H. I.; Marrink S. J. Martini straight: Boosting performance using a shorter cutoff and GPUs. Comput. Phys. Commun. 2016, 199, 1–7. 10.1016/j.cpc.2015.09.014.

[ref64] Berendsen H. J. C.; van Gunsteren W. F. Practical Algorithms for Dynamic Simulation. In Molecular-Dynamics Simulation of Statistical-Mechanical Systems; Enrico Fermi Summer School, 1986; pp 43–.

[ref65] Flory P. J. The Configuration of Real Polymer Chains. J. Chem. Phys. 1949, 17, 303–310. 10.1063/1.1747243.

[ref66] Uversky V. N.; Gillespie J. R.; Millett I. S.; Khodyakova A. V.; Vasilenko R. N.; Vasiliev A. M.; Rodionov I. L.; Kozlovskaya G. D.; Dolgikh D. A.; Fink A. L.; Doniach S.; Permyakov E. A.; Abramov V. M. Zn2+-Mediated Structure Formation and Compaction of the “Natively Unfolded” Human Prothymosin α. Biochem. Biophys. Res. Commun. 2000, 267, 663–668. 10.1006/bbrc.1999.2013. 10631119

[ref67] Ahmed M. C.; Skaanning L. K.; Jussupow A.; Newcombe E. A.; Kragelund B. B.; Camilloni C.; Langkilde A. E.; Lindorff-Larsen K. Refinement of α-Synuclein Ensembles Against SAXS Data: Comparison of Force Fields and Methods. Frontiers in Molecular Biosciences 2021, 8, na 10.3389/fmolb.2021.654333. PMC8100456 33968988

[ref68] Jephthah S.; Månsson L.; Belić D.; Morth J.; Skepö M. Physicochemical Characterisation of KEIF—The Intrinsically Disordered N-Terminal Region of Magnesium Transporter A. Biomolecules 2020, 10, 623 10.3390/biom10040623. 32316569 PMC7226168

[ref69] Fagerberg E.; Månsson L. K.; Lenton S.; Skepö M. The Effects of Chain Length on the Structural Properties of Intrinsically Disordered Proteins in Concentrated Solutions. J. Phys. Chem. B 2020, 124, 11843–11853. 10.1021/acs.jpcb.0c09635. 33337879 PMC7872433

[ref70] Kyte J.; Doolittle R. F. A simple method for displaying the hydropathic character of a protein. J. Mol. Biol. 1982, 157, 105–132. 10.1016/0022-2836(82)90515-0. 7108955

[ref71] Ishida T.; Kinoshita K. PrDOS: prediction of disordered protein regions from amino acid sequence. Nucleic Acids Res. 2007, 35, W460–W464. 10.1093/nar/gkm363. 17567614 PMC1933209

[ref72] Holehouse A. S.; Das R. K.; Ahad J. N.; Richardson M. O. G.; Pappu R. V. CIDER: Resources to Analyze Sequence-Ensemble Relationships of Intrinsically Disordered Proteins. Biophys. J. 2017, 112, 16–21. 10.1016/j.bpj.2016.11.3200. 28076807 PMC5232785

