# Supplementary material for: Comparative Performance of Computer Simulation Models of Intrinsically Disordered Proteins at Different Levels of Coarse-Graining
Source: J Chem Inf Model. 2023 Jun 20;63(13):4079–87. doi: 10.1021/acs.jcim.3c00113 (PMC10336962; doi:10.1021/acs.jcim.3c00113)
Supplement: Supplementary file 1 — ci3c00113_si_001.pdf [file ci3c00113_si_001.pdf]

Supporting Information
Comparative performance of computer simulation models of intrinsically disordered proteins at different levels of coarse-graining

Eric Fagerberg[†] and Marie Skepö[*,†,‡]

†Theoretical Chemistry, Lund University, POB 124, SE-221 00 Lund, Sweden

‡LINXS - Lund Institute of Advanced Neutron and X-ray Science, Scheelevägen 19, SE-223 70 Lund, Sweden

E-mail: marie.skepo@teokem.lu.se

1 Computed chain properties for protein data set

Table S1: Properties of the proteins in the data set. N=Number of amino acids in sequence, FCR=Fraction of Charged Residues, NCPR=Net Charge Per Residue. Hydrophobicity uses the Kyte-Doolittle scale. The Disorder score is based on the PrDOS algorithm.

Chain	N	FCR	NCPR	Hydrophobicity	Proline content	Disorder score
Hst5	24	0.375	0.208	2.046	0.000	0.890
ACTR	71	0.254	-0.113	3.828	0.070	0.491
Nucleoporin	81	0	0	4.080	0.173	0.634
SH4UD	85	0.188	0.047	3.768	0.118	0.667
Sic1	90	0.122	0.122	3.652	0.167	0.599
p53	93	0.204	-0.161	3.967	0.237	0.496
Proth. α	111	0.577	-0.396	2.529	0.001	0.732
ERM TADn	122	0.279	-0.082	3.761	0.074	0.474
hNHE1	131	0.298	-0.099	3.683	0.145	0.561
Alp. Syn.	140	0.279	-0.064	4.097	0.036	0.530
An16	185	0	0	3.504	0.178	0.729
Osteopontin	273	0.348	-0.158	3.397	0.040	0.614
K19	99	0.253	0.071	3.828	0.071	0.467
K18	130	0.246	0.077	3.855	0.062	0.481
K17	143	0.241	0.103	3.710	0.124	0.558
K10	167	0.238	0.036	3.951	0.060	0.494
K27	171	0.251	0.096	3.696	0.108	0.564
K16	174	0.239	0.102	3.750	0.108	0.562
K25	185	0.281	0.022	3.353	0.130	0.701
K32	202	0.247	0.096	3.734	0.096	0.565
K23	254	0.264	0.012	3.563	0.106	0.645
K44	283	0.272	0.039	3.508	0.110	0.664
hTau23	352	0.261	0.028	3.630	0.097	0.652
hTau40	441	0.259	0.005	3.632	0.098	0.637

2 Radius of gyration for each replicate

Table S2: The computed radius of gyration for each replicate for each system, including standard deviations, using the MARTINI Stark model. "Start R_g" refers to the radius of gyration of the starting structure predicted by the RoseTTAFold algorithm, after transformation to the MARTINI coarse-grained representation. BSE refers to the Block average Standard Error (in units of Å), as computed with the Gromacs tool *gmx analyze*. In the case of Prothymosin α, no estimate could be produced by this tool.

Chain	Start R_g	Sim length (ns)	Rep 1	Rep2	Rep 3	BSE
Hst5	14.9	5000	14.4 ± 1.4	14.5 ± 1.4	14.4 ± 1.4	0.02
ACTR	46.6	5000	24.1 ± 4.9	24.4 ± 4.4	24.5 ± 5.3	0.4
Nucleoporin	34.6	5000	29.7 ± 5.0	29.5 ± 4.6	30.0 ± 5.0	0.2
SH4-UD	41.6	7500	31.9 ± 1.0	23.8 ± 1.0	23.7 ± 6.0	4.2
Sic1	39.1	5000	34.0 ± 6.0	32.5 ± 5.3	33.7 ± 5.6	0.3
p53	32.1	5000	27.7 ± 5.5	27.5 ± 5.1	26.9 ± 4.9	0.3
Proth. α	48.9	10 000	21.5 ± 3.1	38.9 ± 1.3	51.2 ± 0.8	-
ERM TADn	37.6	5000	31.0 ± 5.5	30.8 ± 6.0	30.9 ± 5.3	0.4
hNHE1	34.5	5000	31.9 ± 7.8	32.5 ± 6.3	29.4 ± 5.8	0.6
Alp. Syn.	28.5	5000	27.8 ± 5.1	28.6 ± 5.9	30.1 ± 6.3	1.9
An16	48.2	5000	49.5 ± 8.0	49.3 ± 9.7	49.6 ± 8.9	0.7
Ostepontin	56.2	7500	59.1 ± 9.9	62.6 ± 11.5	59.9 ± 9.8	1.3
K19	36.4	5000	28.6 ± 5.4	28.8 ± 5.8	28.7 ± 5.6	0.5
K18	35.8	7700	28.3 ± 3.3	35.0 ± 7.5	34.3 ± 7.0	2.3
K17	53.8	5000	34.5 ± 6.7	37.2 ± 8.1	34.6 ± 7.3	2.5
K10	24.4	5000	36.7 ± 8.2	34.2 ± 6.7	37.9 ± 8.1	0.8
K27	45.6	5000	38.8 ± 9.2	39.5 ± 8.5	39.6 ± 8.9	1.1
K16	45.8	5000	39.4 ± 8.4	41.4 ± 8.9	39.2 ± 8.4	1.0
K25	40.9	5000	33.0 ± 5.8	34.1 ± 7.2	35.5 ± 7.2	0.8
K32	45.6	5000	41.0 ± 8.4	41.0 ± 8.7	38.3 ± 0.7	1.7
K23	63.7	5000	58.4 ± 0.6	39.8 ± 10.0	48.5 ± 12.6	3.9
K44	61.4	7500	49.9 ± 0.7	43.6 ± 9.1	58.9 ± 1.6	5.1
hTau23	77.0	5000	44.4 ± 2.3	57.7 ± 4.5	54.7 ± 11.0	3.5
hTau40	65.5	10000	44.2 ± 1.4	39.8 ± 1.9	38.4 ± 1.3	2.2

Table S3: The computed radius of gyration for each replicate for the extended data set, including standard deviations. Starting structures were chosen as fully extended. BSE refers to the Block average Standard Error (in units of Å), as computed with the Gromacs tool *gmx analyze*.

Chain	Sim length (ns)	Rep 1	Rep2	Rep 3	BSE
(Histatin 5)$_2$	5000	23.4 ± 3.3	23.7 ± 3.2	23.7 ± 3.2	0.1
KEIF	5000	15.9 ± 2.2	16.1 ± 2.3	15.9 ± 2.3	0.1

3 Convergence and contact maps

Hst5

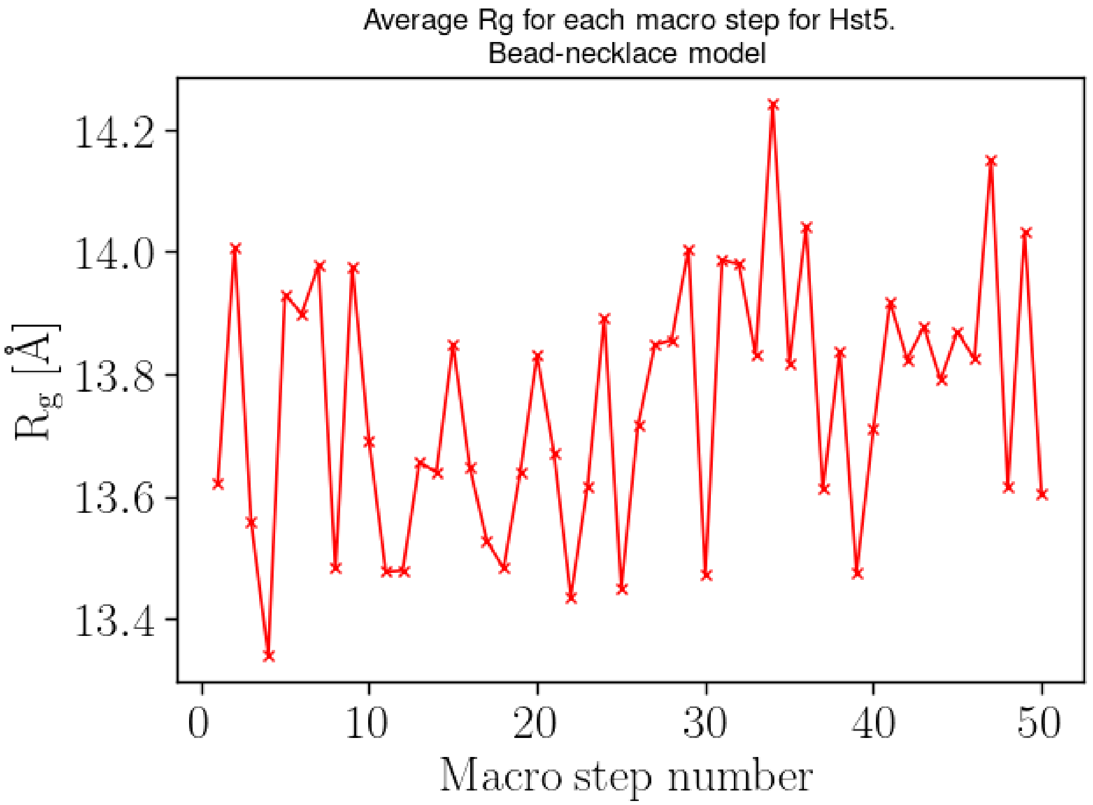

Figure S1: The average R_g for each macrostep (20 000 Monte Carlo steps) in the bead-necklace simulation. Only production run shown here.

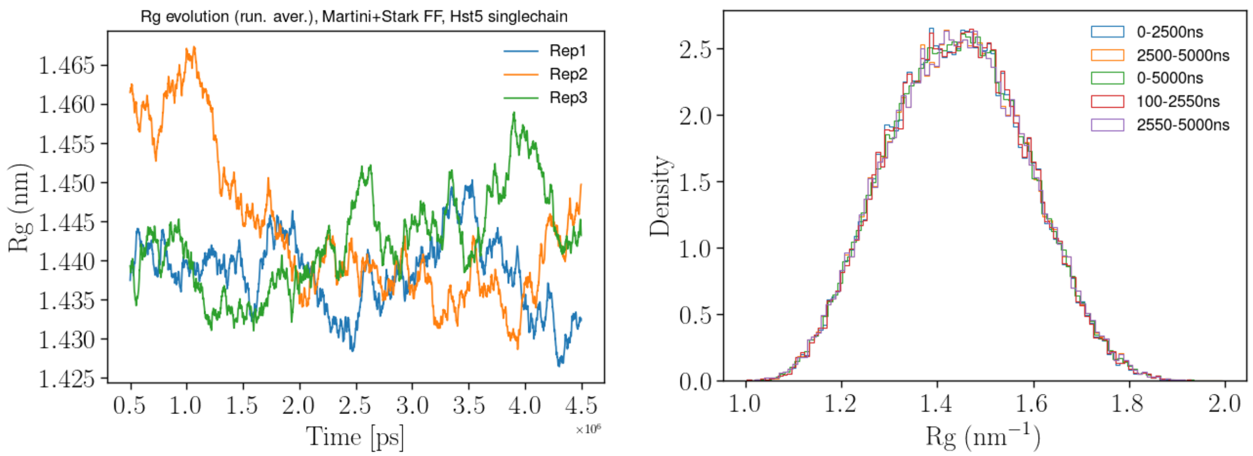

Figure S2: **Left:** Running average of R_g for the different replicate simulations of Hst5. **Right:** Distribution of R_g for all replicates combined, for different parts of the simulation (time-wise).

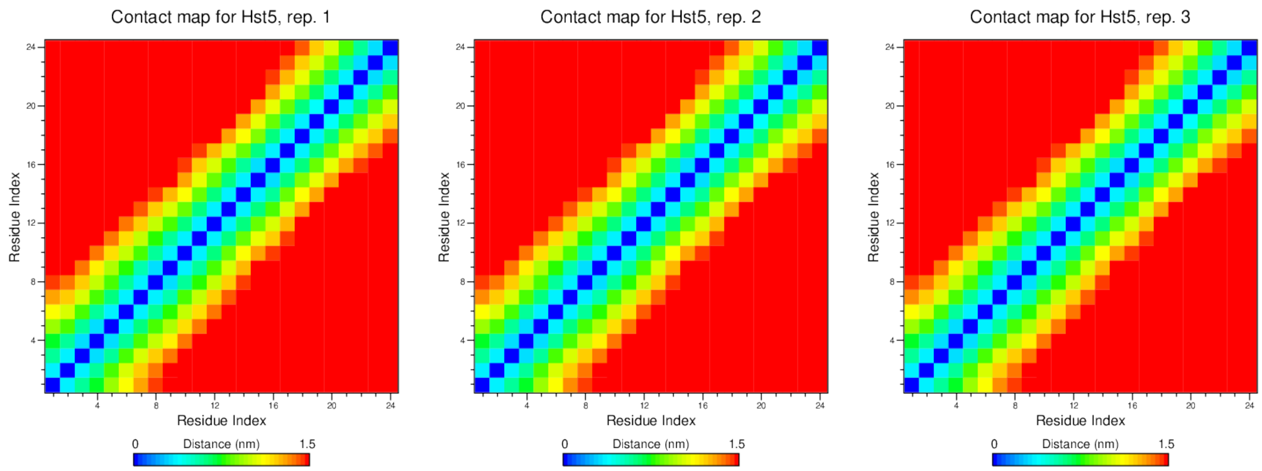

Figure S3: Contact maps from the MARTINI Stark simulation for Hst5. **Left:** Replicate 1. **Middle:** Replicate 2. **Right:** Replicate 3.

ACTR

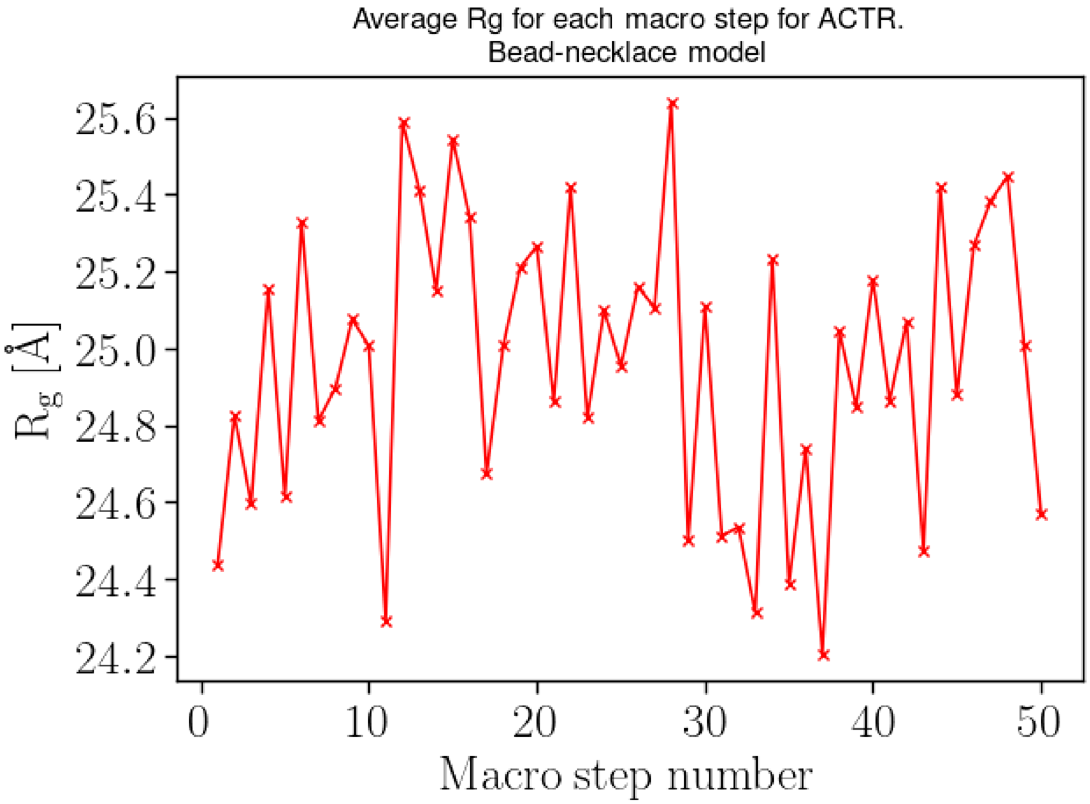

Figure S4: The average R_g for each macrostep (20 000 Monte Carlo steps) in the bead-necklace simulation. Only production run shown here.

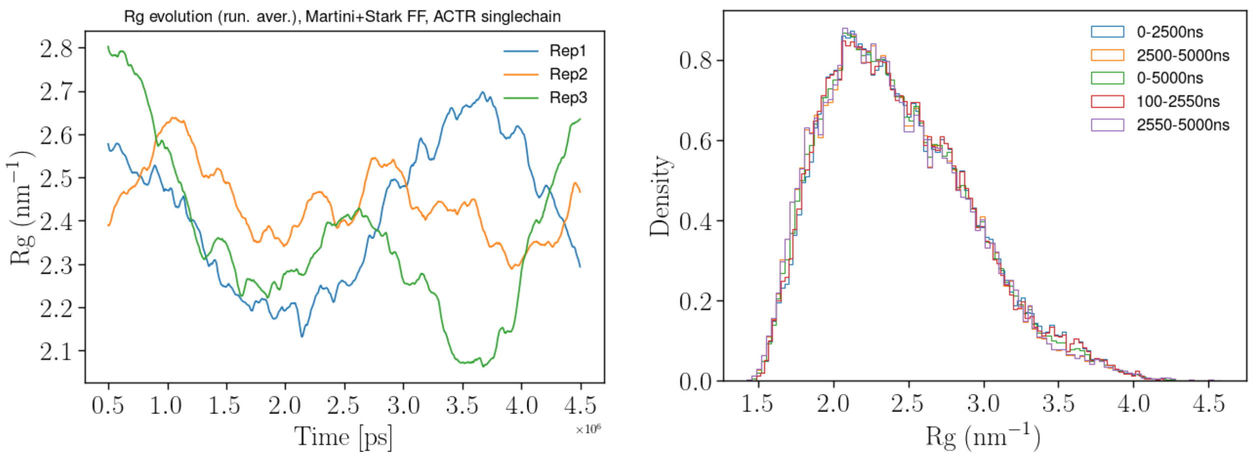

Figure S5: **Left:** Running average of R_g for the different replicate simulations of ACTR. **Right:** Distribution of R_g for all replicates combined, for different parts of the simulation (time-wise).

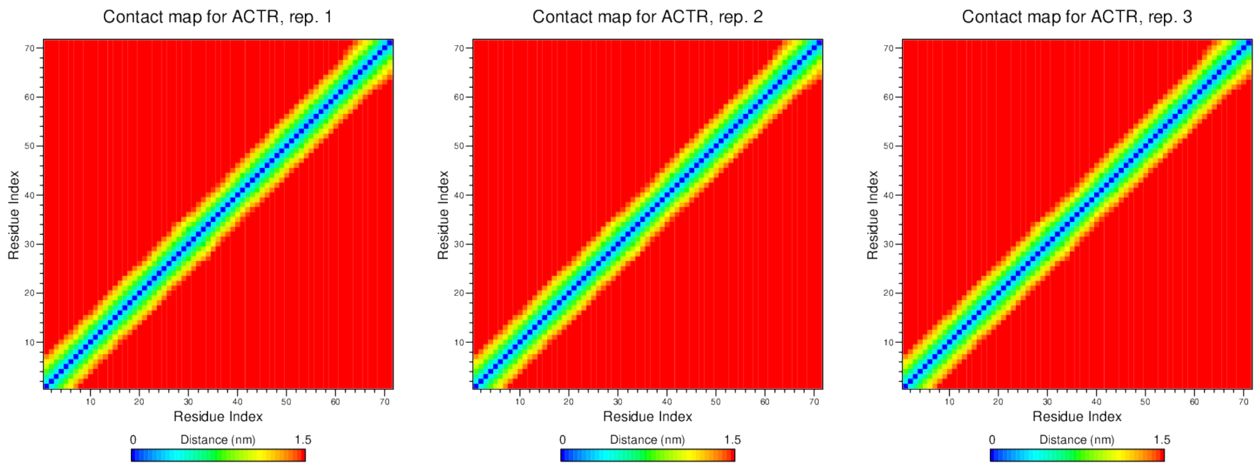

Figure S6: Contact maps from the MARTINI Stark simulation for ACTR. **Left:** Replicate 1. **Middle:** Replicate 2. **Right:** Replicate 3.

Nucleoporin153

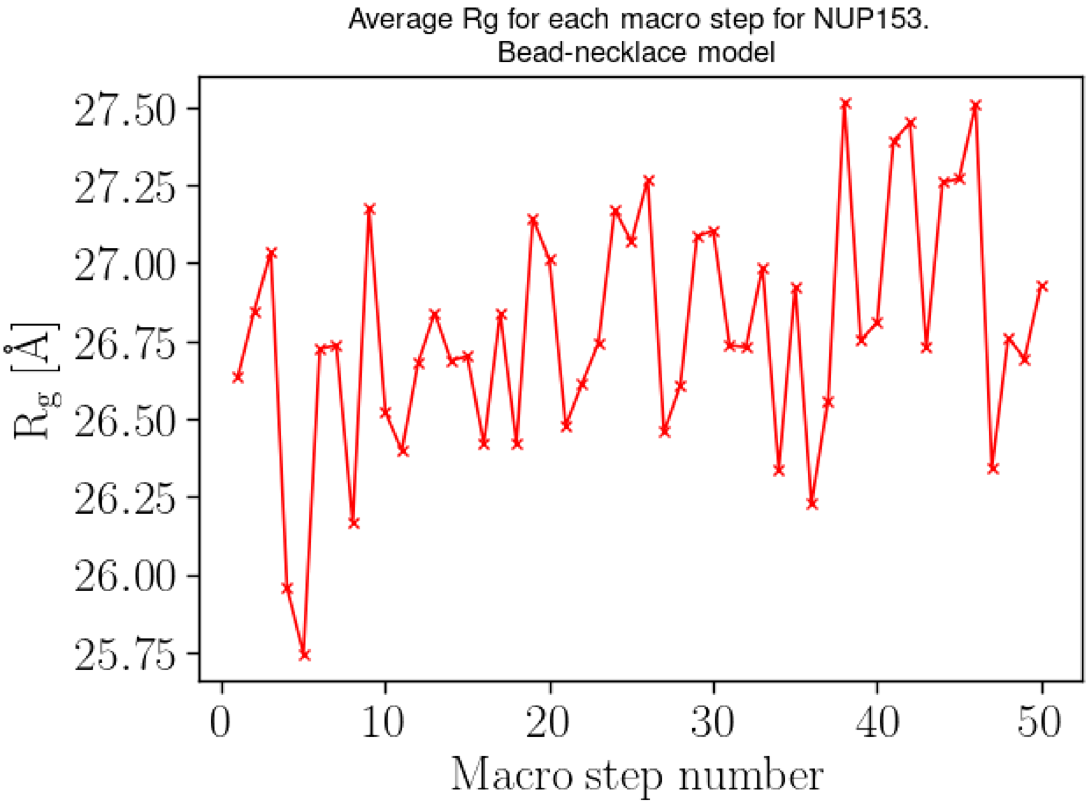

Figure S7: The average R_g for each macrostep (20 000 Monte Carlo steps) in the bead-necklace simulation. Only production run shown here.

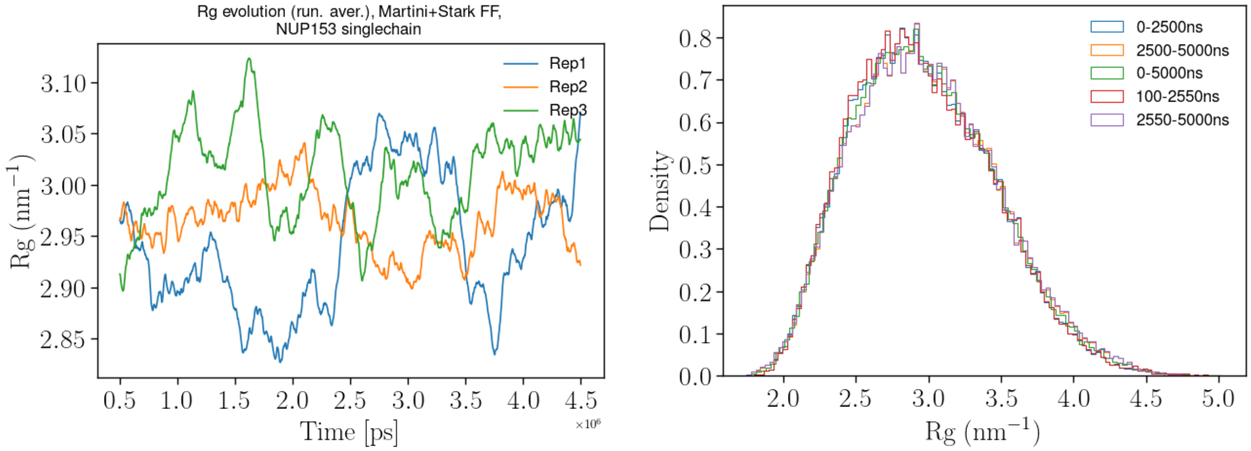

Figure S8: **Left:** Running average of R_g for the different replicate simulations of Nucleoporin153. **Right:** Distribution of R_g for all replicates combined, for different parts of the simulation (time-wise).

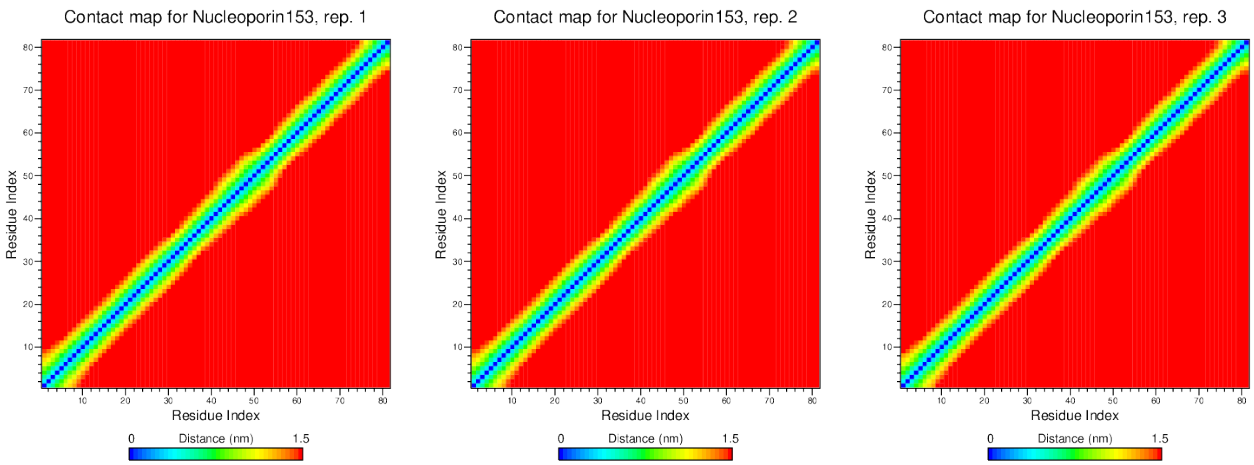

Figure S9: Contact maps from the MARTINI Stark simulation for Nucleoporin153. **Left:** Replicate 1. **Middle:** Replicate 2. **Right:** Replicate 3.

SH4UD

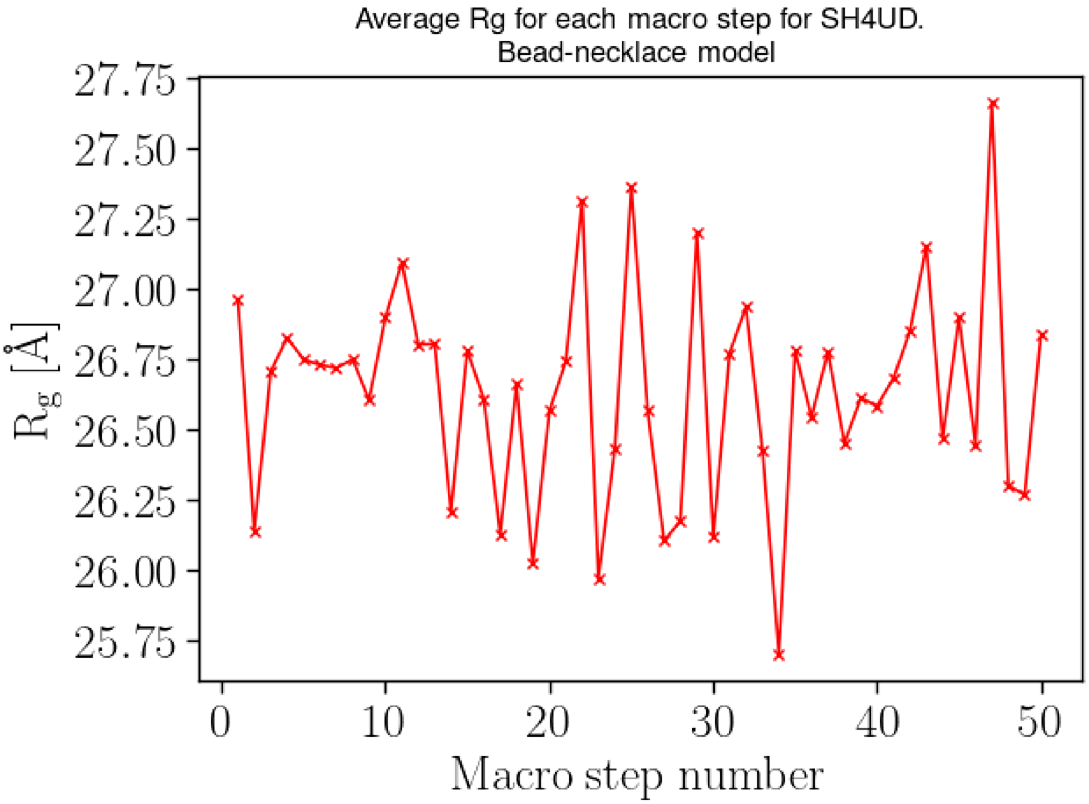

Figure S10: The average R_g for each macrostep (20 000 Monte Carlo steps) in the bead-necklace simulation. Only production run shown here.

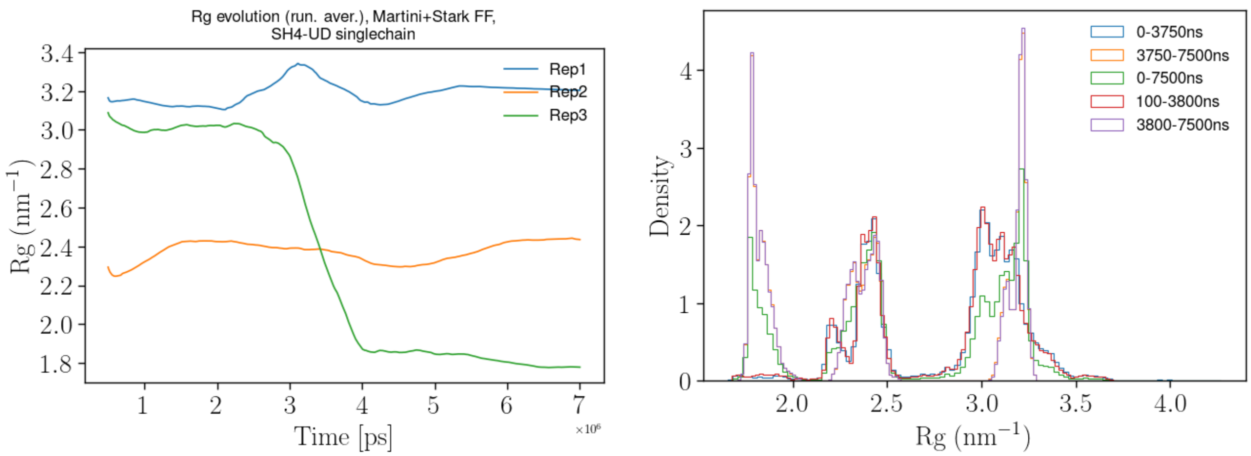

Figure S11: **Left:** Running average of R_g for the different replicate simulations of SH4-UD. **Right:** Distribution of R_g for all replicates combined, for different parts of the simulation (time-wise).

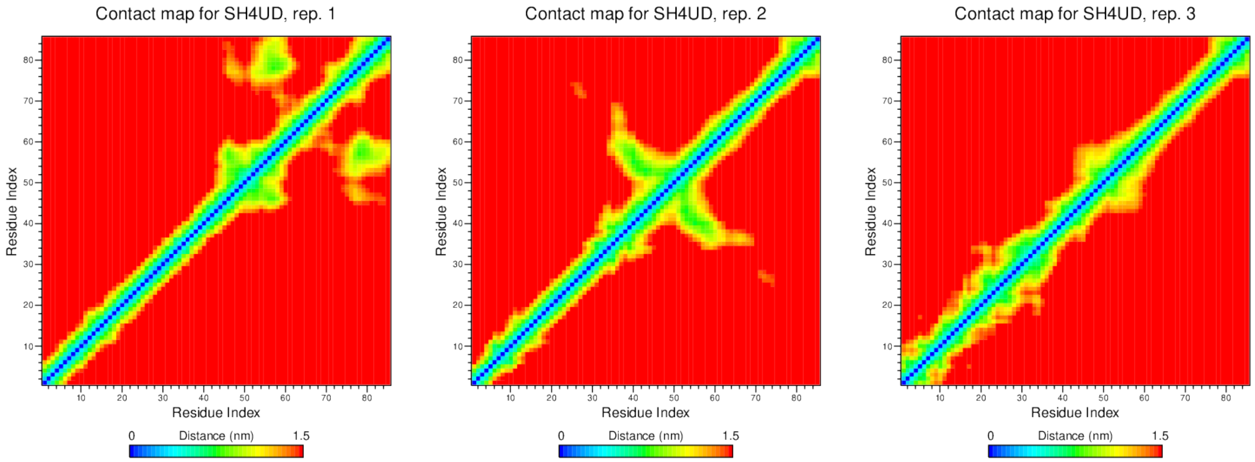

Figure S12: Contact maps from the MARTINI Stark simulation for SH4UD. **Left:** Replicate 1. **Middle:** Replicate 2. **Right:** Replicate 3.

Sic1

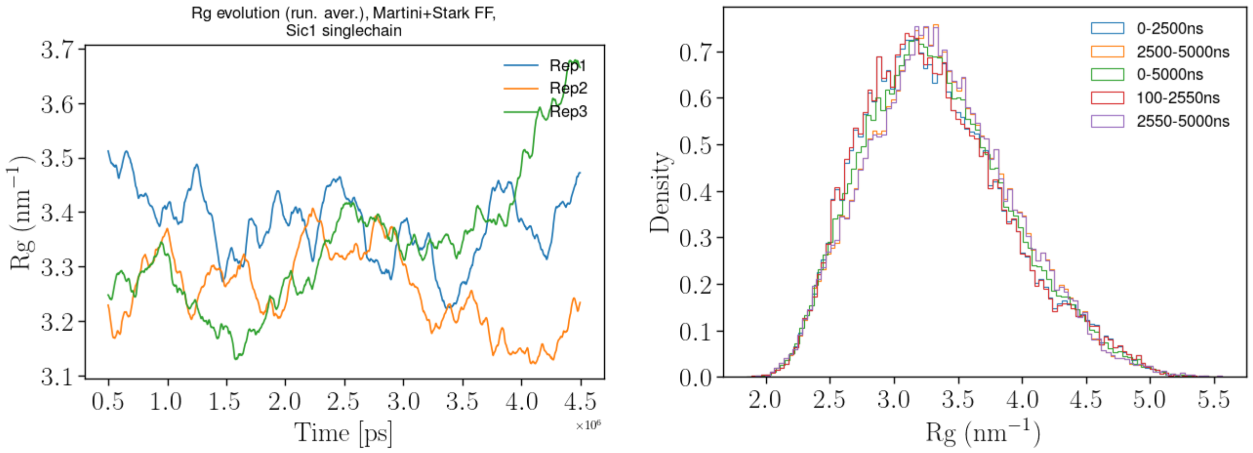

Figure S13: **Left:** Running average of R_g for the different replicate simulations of Sic1. **Right:** Distribution of R_g for all replicates combined, for different parts of the simulation (time-wise).

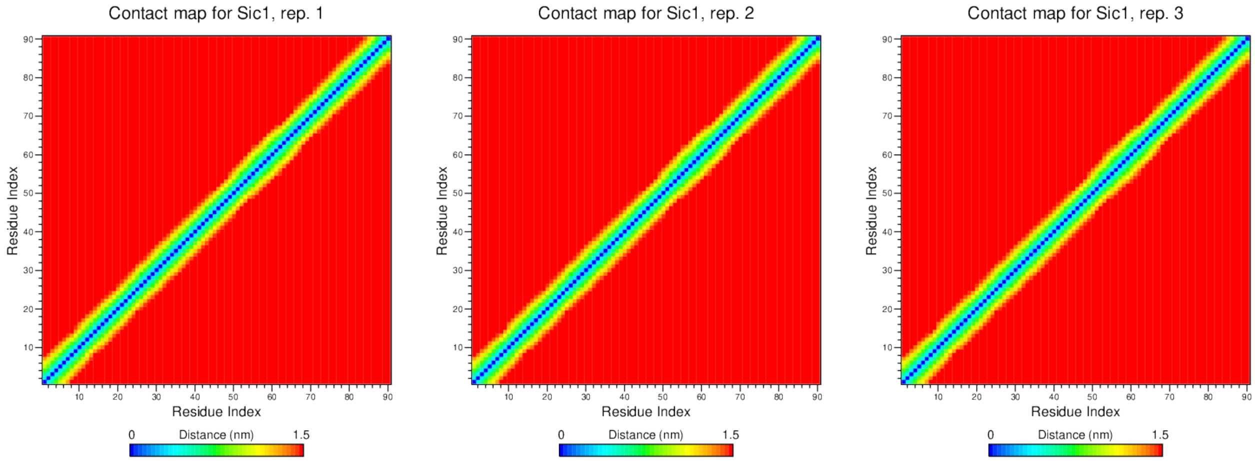

Figure S14: Contact maps from the MARTINI Stark simulation for Sic1. **Left:** Replicate 1. **Middle:** Replicate 2. **Right:** Replicate 3.

p53

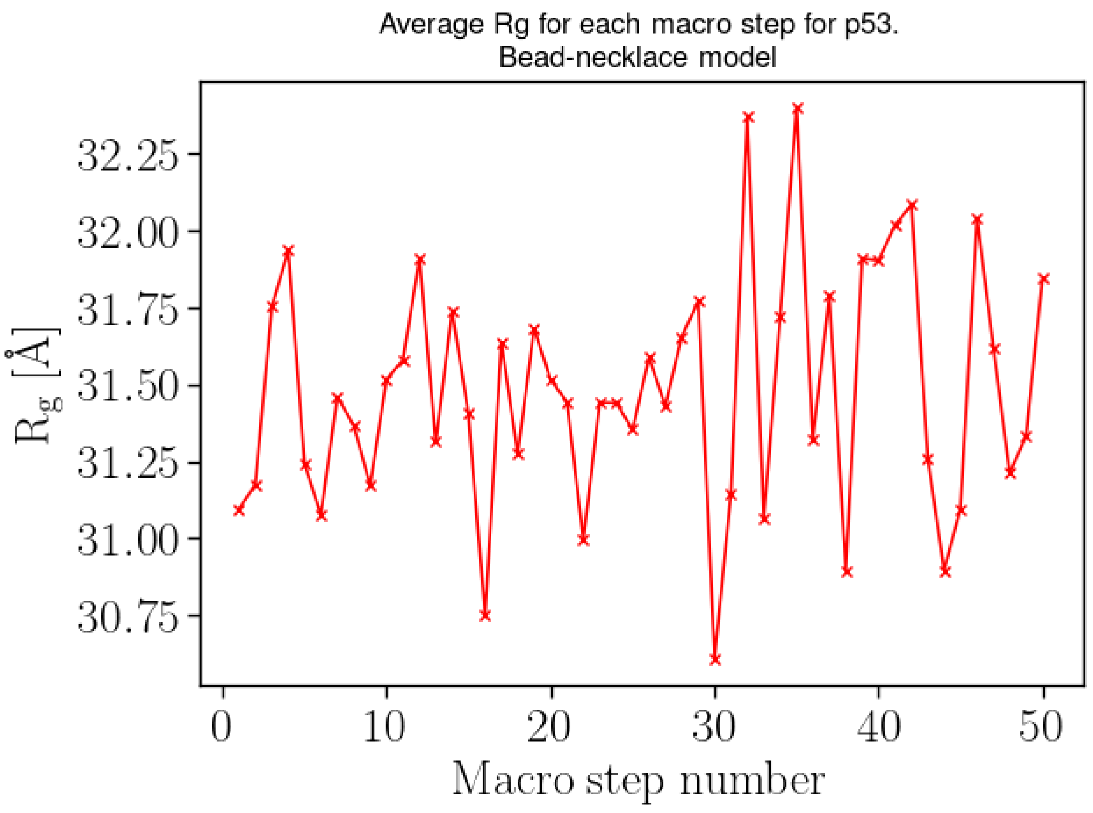

Figure S15: The average R_g for each macrostep (20 000 Monte Carlo steps) in the bead-necklace simulation. Only production run shown here.

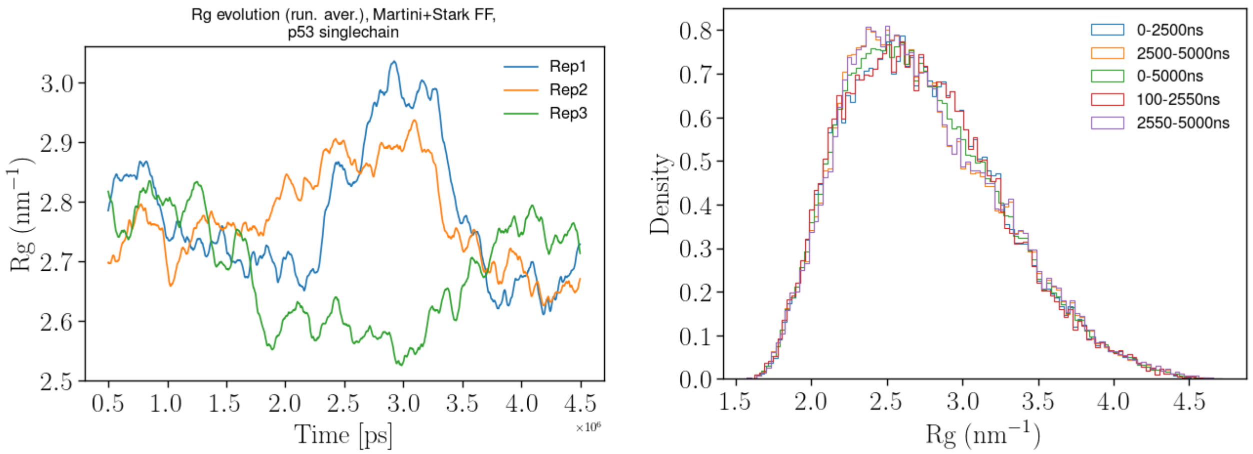

Figure S16: **Left:** Running average of R_g for the different replicate simulations of p53. **Right:** Distribution of R_g for all replicates combined, for different parts of the simulation (time-wise).

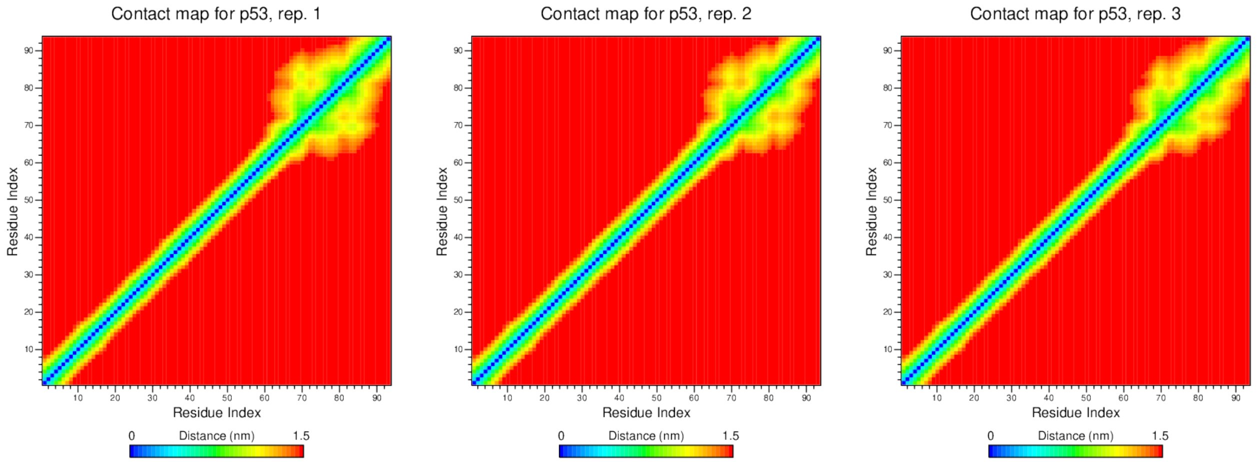

Figure S17: Contact maps from the MARTINI Stark simulation for p53. **Left:** Replicate 1. **Middle:** Replicate 2. **Right:** Replicate 3.

Prothymosin α

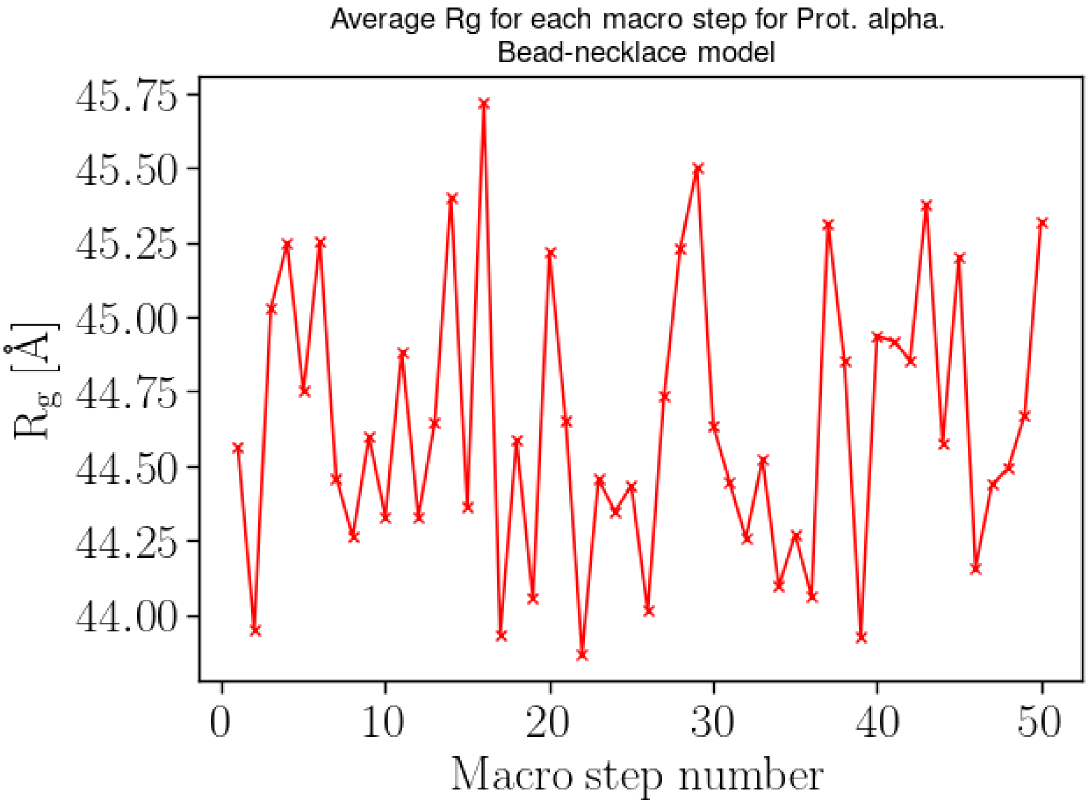

Figure S18: The average R_g for each macrostep (20 000 Monte Carlo steps) in the bead-necklace simulation. Only production run shown here.

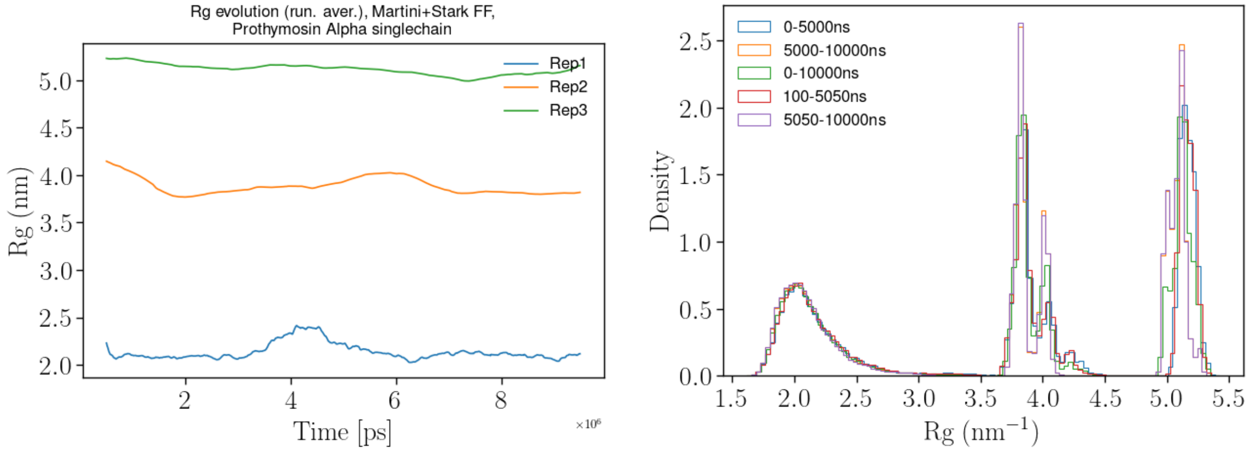

Figure S19: **Left:** Running average of R_g for the different replicate simulations of Prothymosin α. **Right:** Distribution of R_g for all replicates combined, for different parts of the simulation (time-wise).

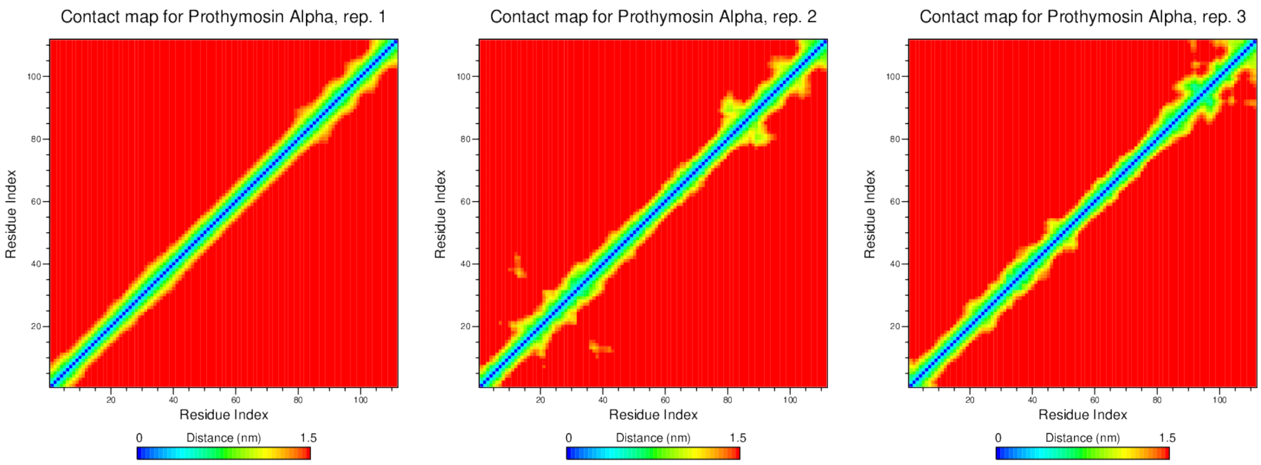

Figure S20: Contact maps from the MARTINI Stark simulation for Prothymosin α. **Left:** Replicate 1. **Middle:** Replicate 2. **Right:** Replicate 3.

ERMTADn

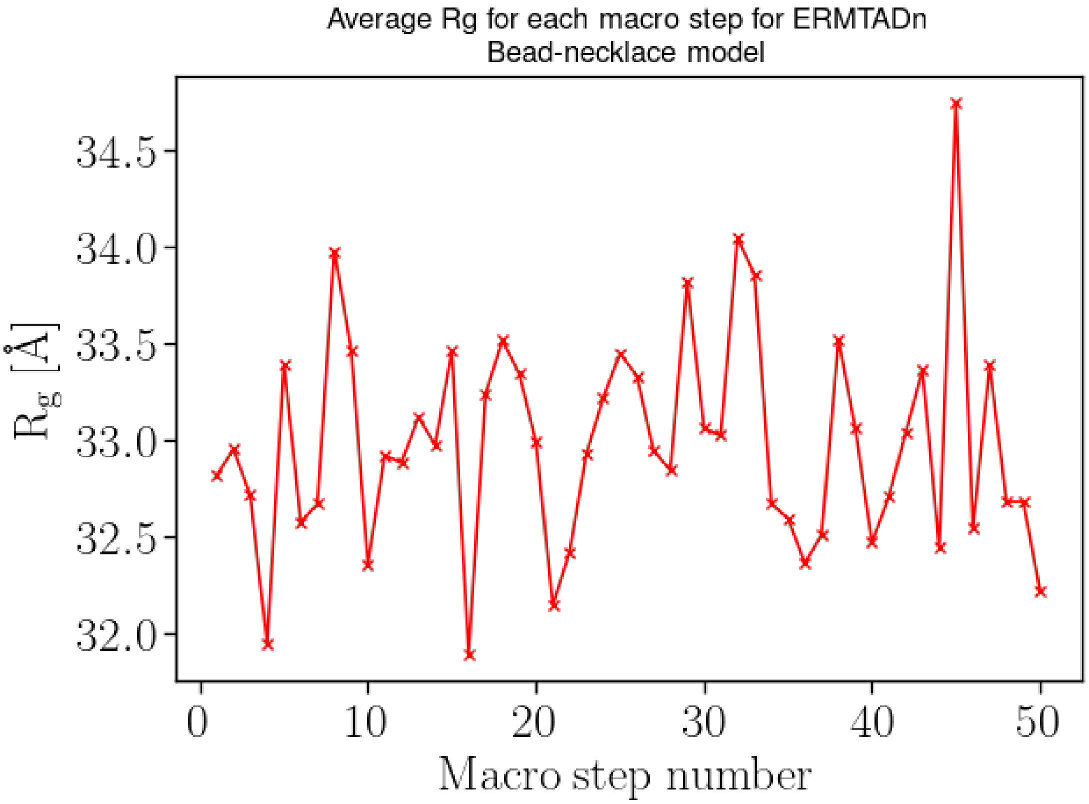

Figure S21: The average R_g for each macrostep (20 000 Monte Carlo steps) in the bead-necklace simulation. Only production run shown here.

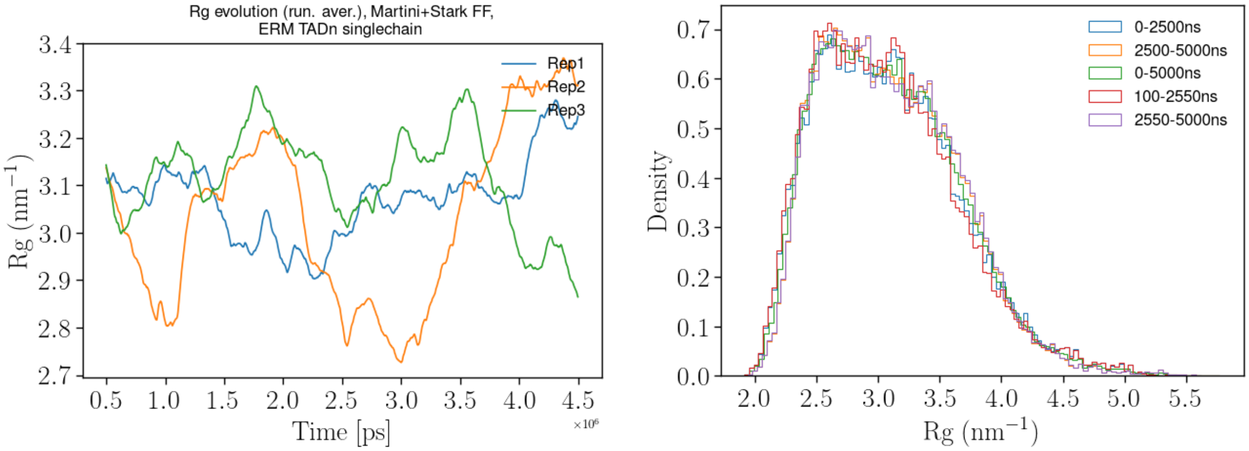

Figure S22: **Left:** Running average of R_g for the different replicate simulations of ERM TADn. **Right:** Distribution of R_g for all replicates combined, for different parts of the simulation (time-wise).

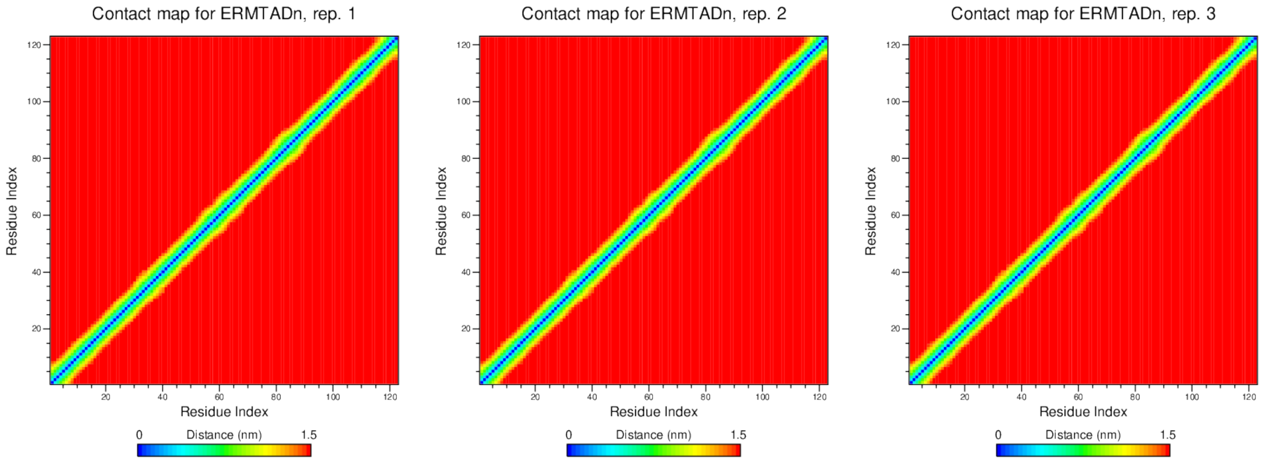

Figure S23: Contact maps from the MARTINI Stark simulation for ERMTADn. **Left:** Replicate 1. **Middle:** Replicate 2. **Right:** Replicate 3.

hNHE1

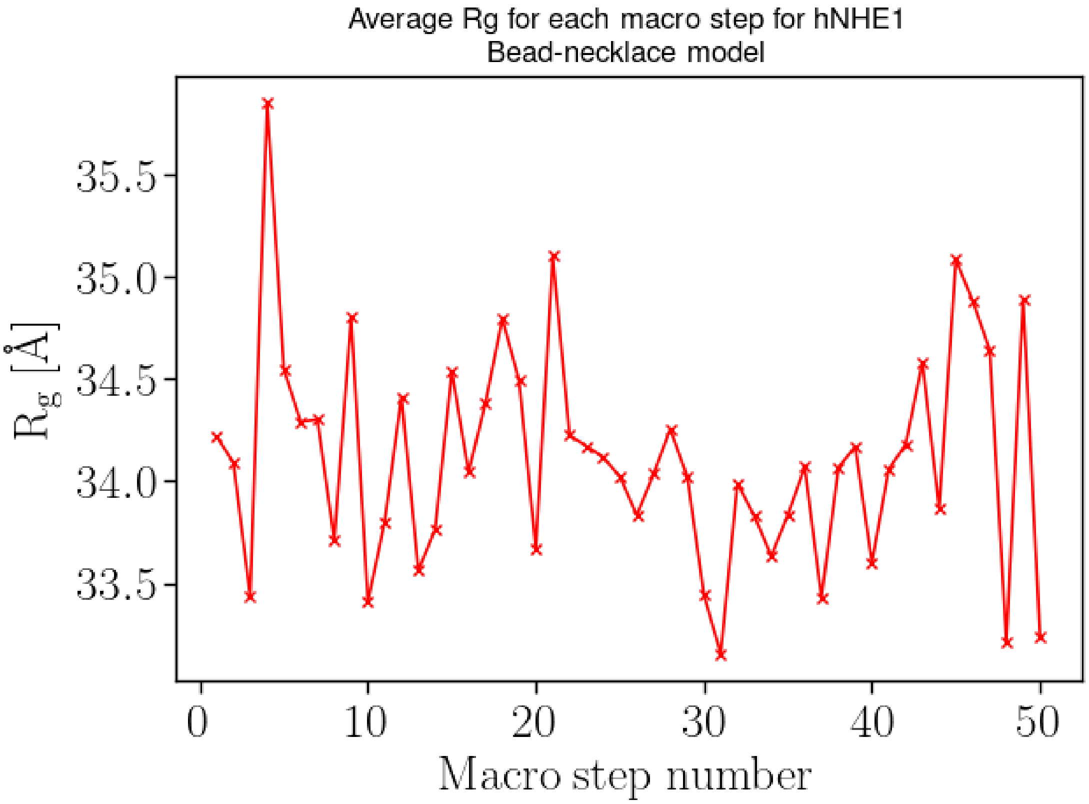

Figure S24: The average R_g for each macrostep (20 000 Monte Carlo steps) in the bead-necklace simulation. Only production run shown here.

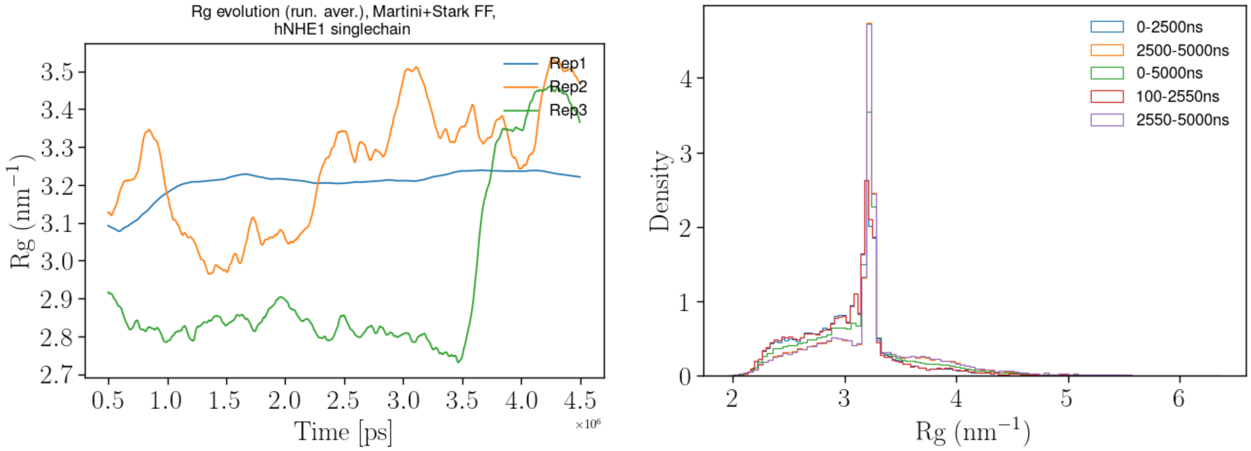

Figure S25: **Left:** Running average of R_g for the different replicate simulations of hNHE1. **Right:** Distribution of R_g for all replicates combined, for different parts of the simulation (time-wise).

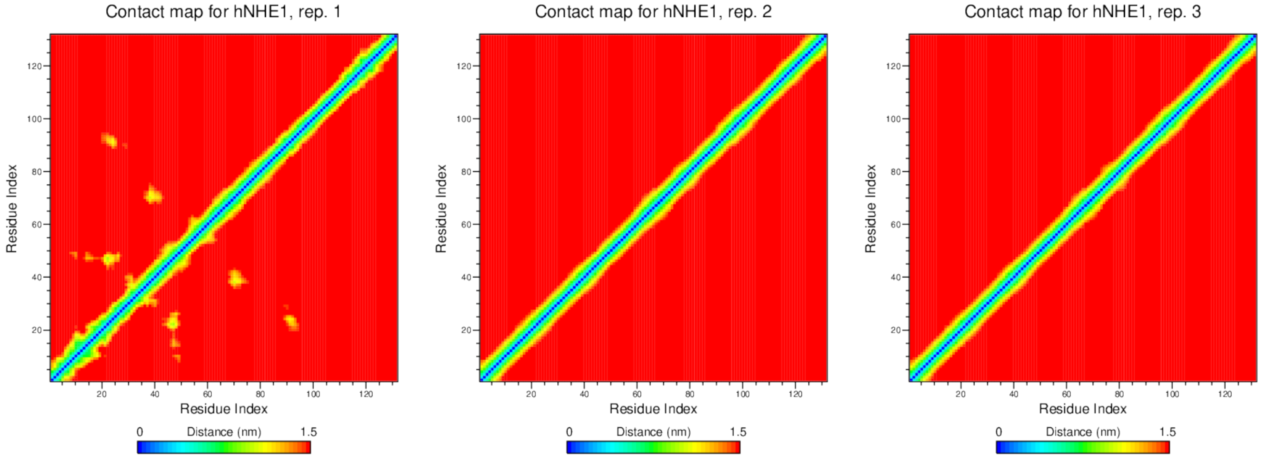

Figure S26: Contact maps from the MARTINI Stark simulation for hNHE1. **Left:** Replicate 1. **Middle:** Replicate 2. **Right:** Replicate 3.

α - Synuclein

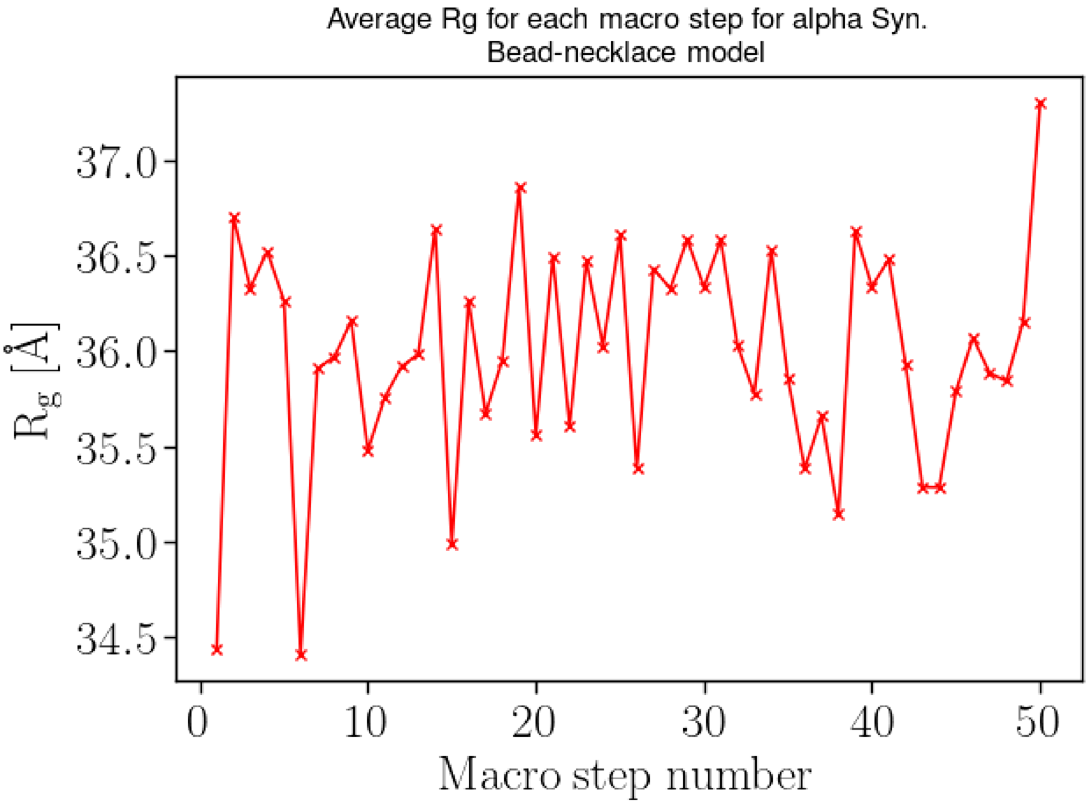

Figure S27: The average R_g for each macrostep (20 000 Monte Carlo steps) in the bead-necklace simulation. Only production run shown here.

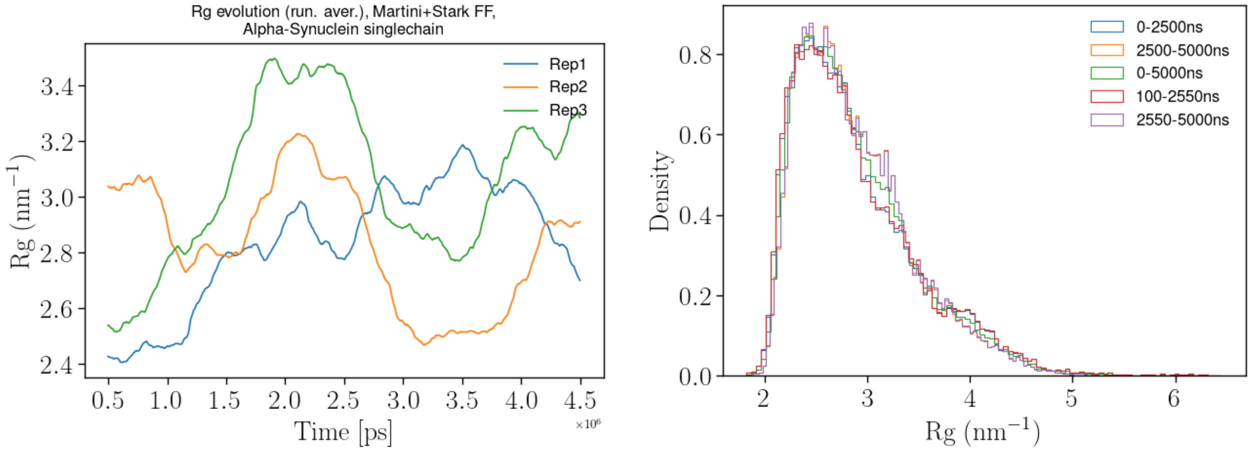

Figure S28: **Left:** Running average of R_g for the different replicate simulations of α Synuclein. **Right:** Distribution of R_g for all replicates combined, for different parts of the simulation (time-wise).

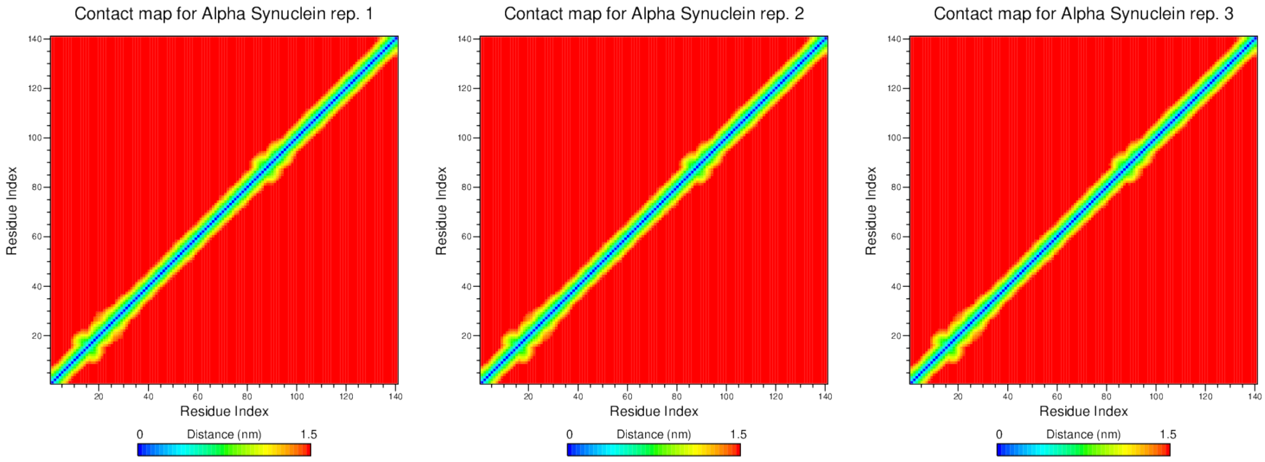

Figure S29: Contact maps from the MARTINI Stark simulation for α Synuclein. **Left:** Replicate 1. **Middle:** Replicate 2. **Right:** Replicate 3.

An16

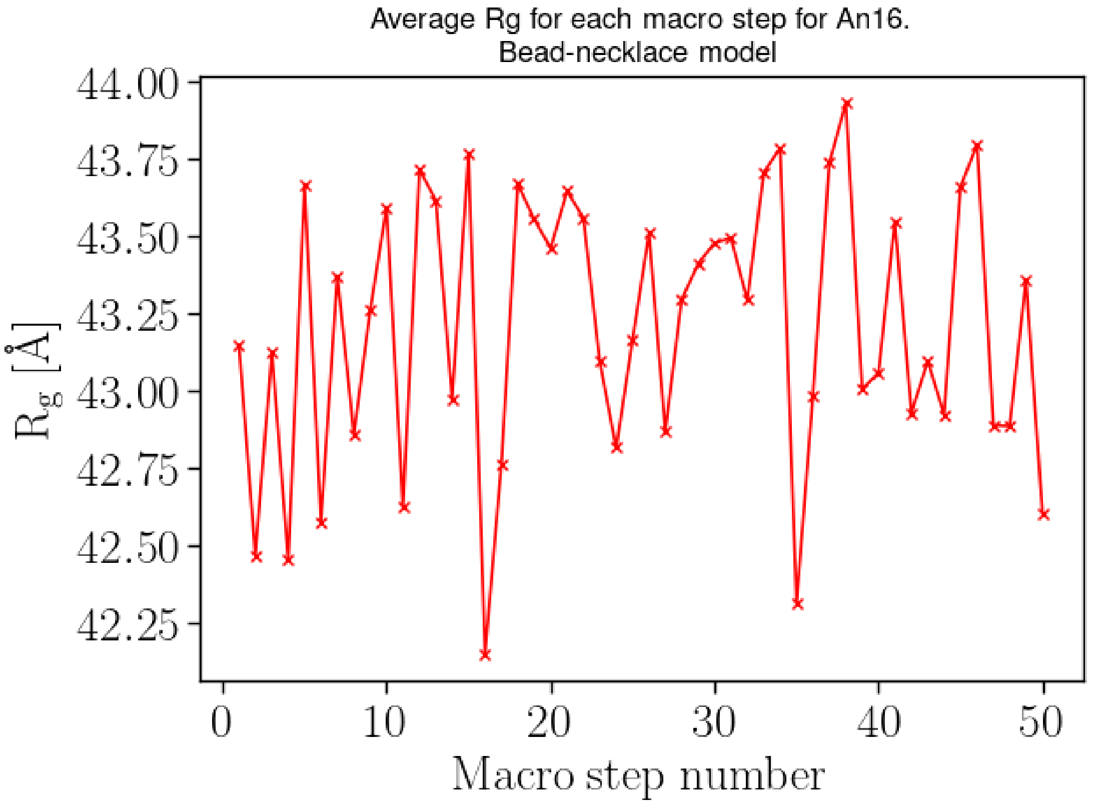

Figure S30: The average R_g for each macrostep (20 000 Monte Carlo steps) in the bead-necklace simulation. Only production run shown here.

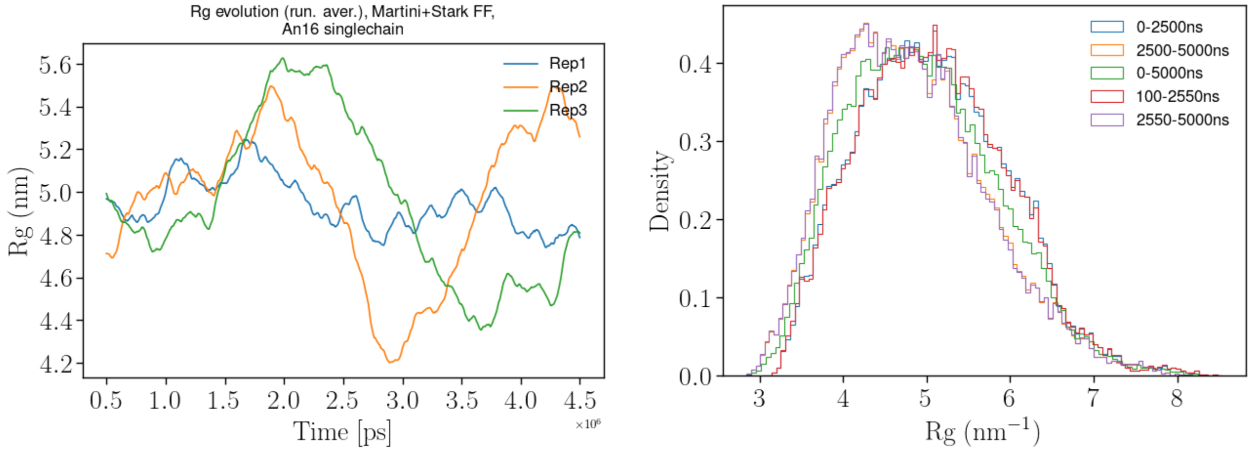

Figure S31: **Left:** Running average of R_g for the different replicate simulations of An16. **Right:** Distribution of R_g for all replicates combined, for different parts of the simulation (time-wise).

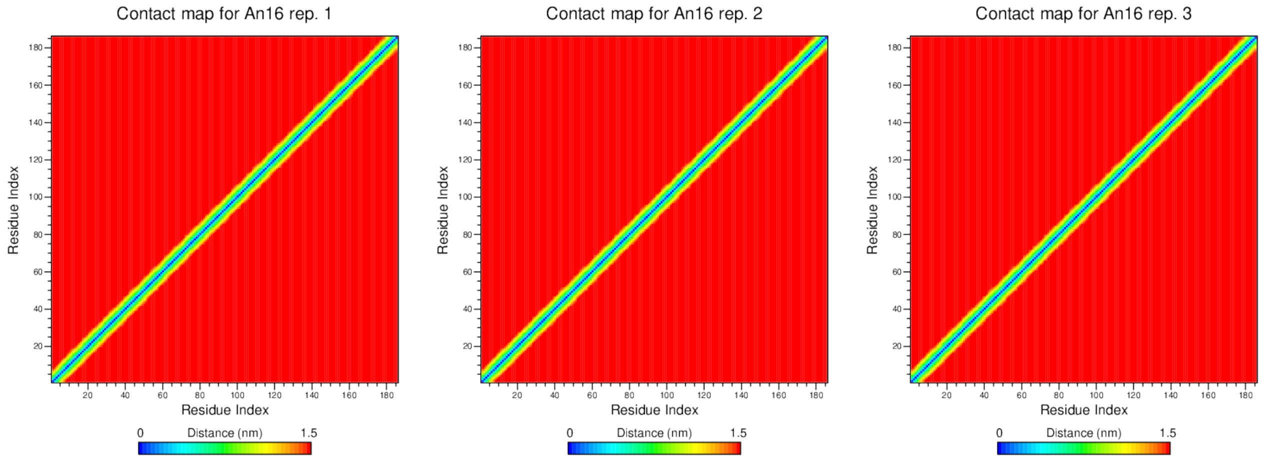

Figure S32: Contact maps from the MARTINI Stark simulation for An16. **Left:** Replicate 1. **Middle:** Replicate 2. **Right:** Replicate 3.

Osteopontin

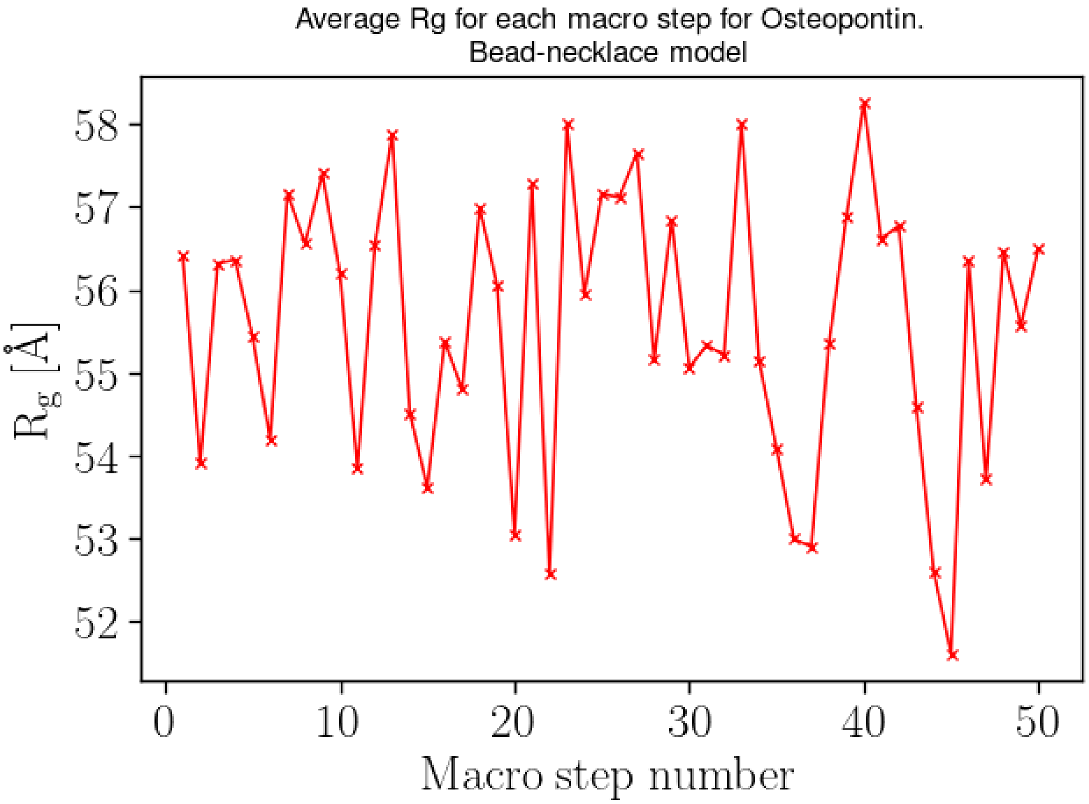

Figure S33: The average R_g for each macrostep (20 000 Monte Carlo steps) in the bead-necklace simulation. Only production run shown here.

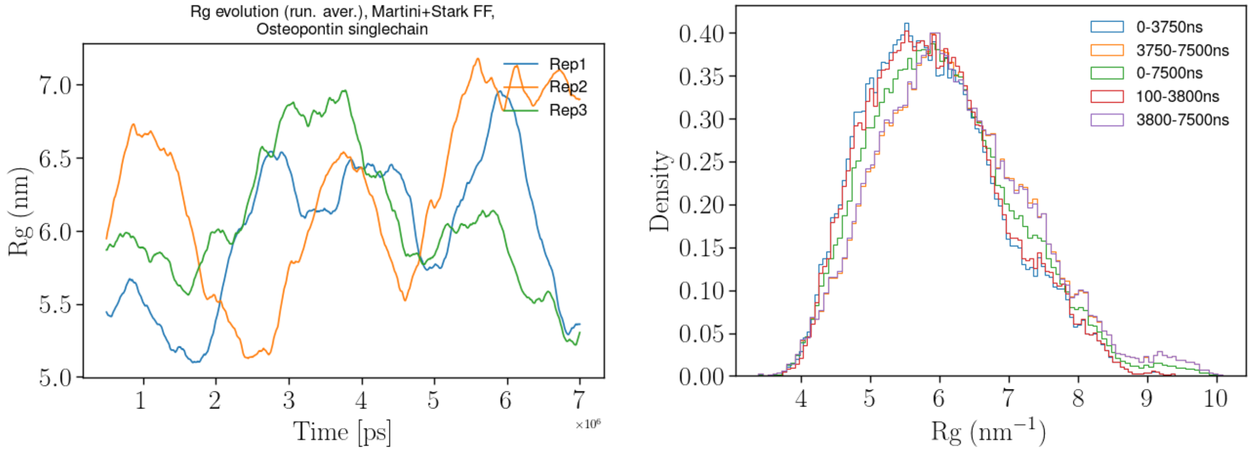

Figure S34: **Left:** Running average of R_g for the different replicate simulations of Osteopontin. **Right:** Distribution of R_g for all replicates combined, for different parts of the simulation (time-wise).

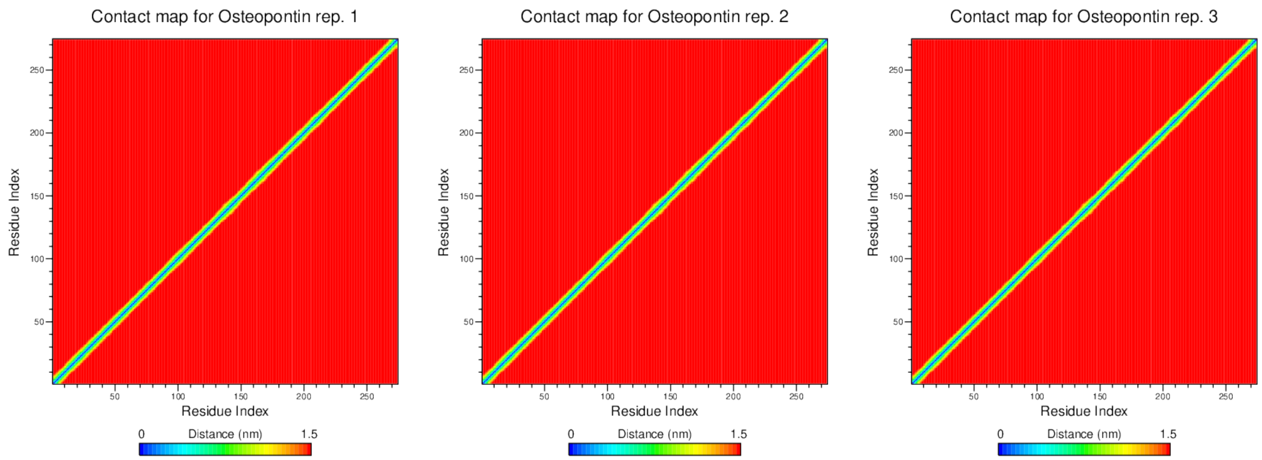

Figure S35: Contact maps from the MARTINI Stark simulation for Osteopontin. **Left:** Replicate 1. **Middle:** Replicate 2. **Right:** Replicate 3.

K19

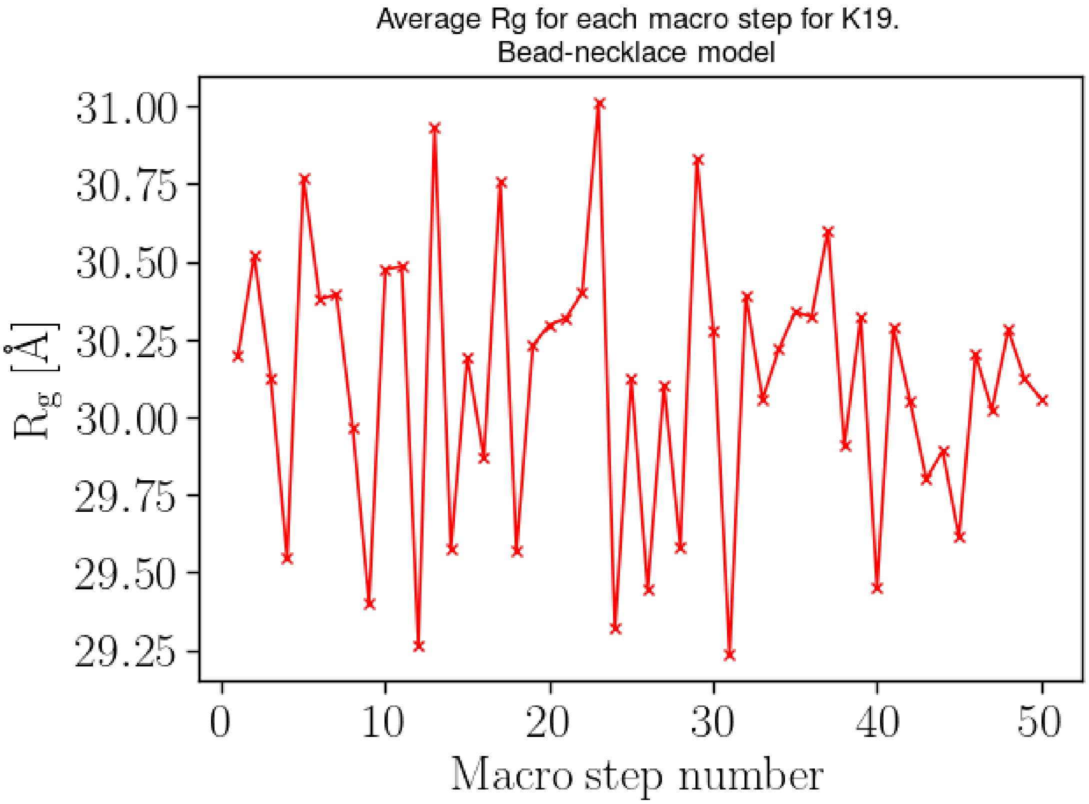

Figure S36: The average R_g for each macrostep (20 000 Monte Carlo steps) in the bead-necklace simulation. Only production run shown here.

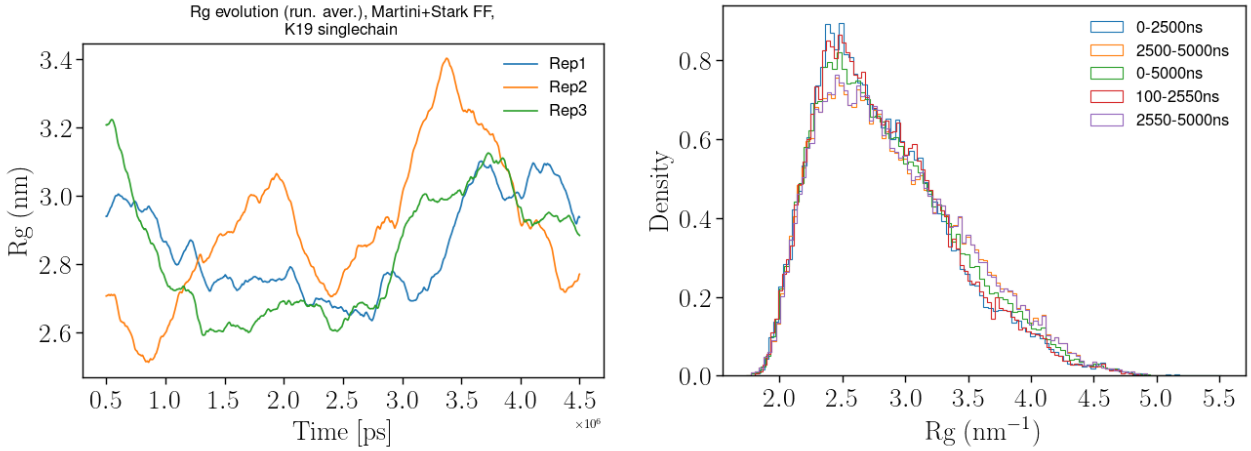

Figure S37: **Left:** Running average of R_g for the different replicate simulations of K19. **Right:** Distribution of R_g for all replicates combined, for different parts of the simulation (time-wise).

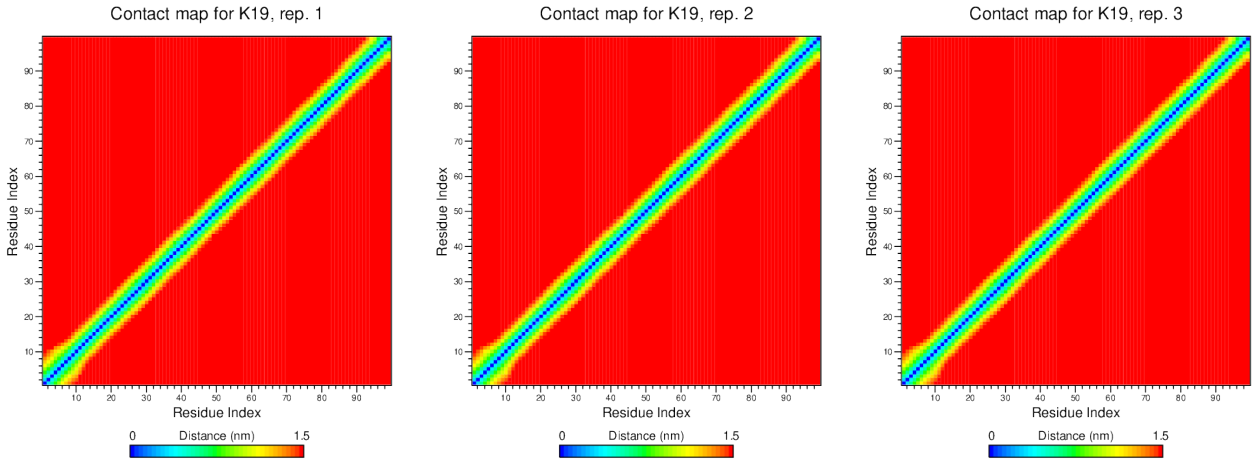

Figure S38: Contact maps from the MARTINI Stark simulation for K19. **Left:** Replicate 1. **Middle:** Replicate 2. **Right:** Replicate 3.

K18

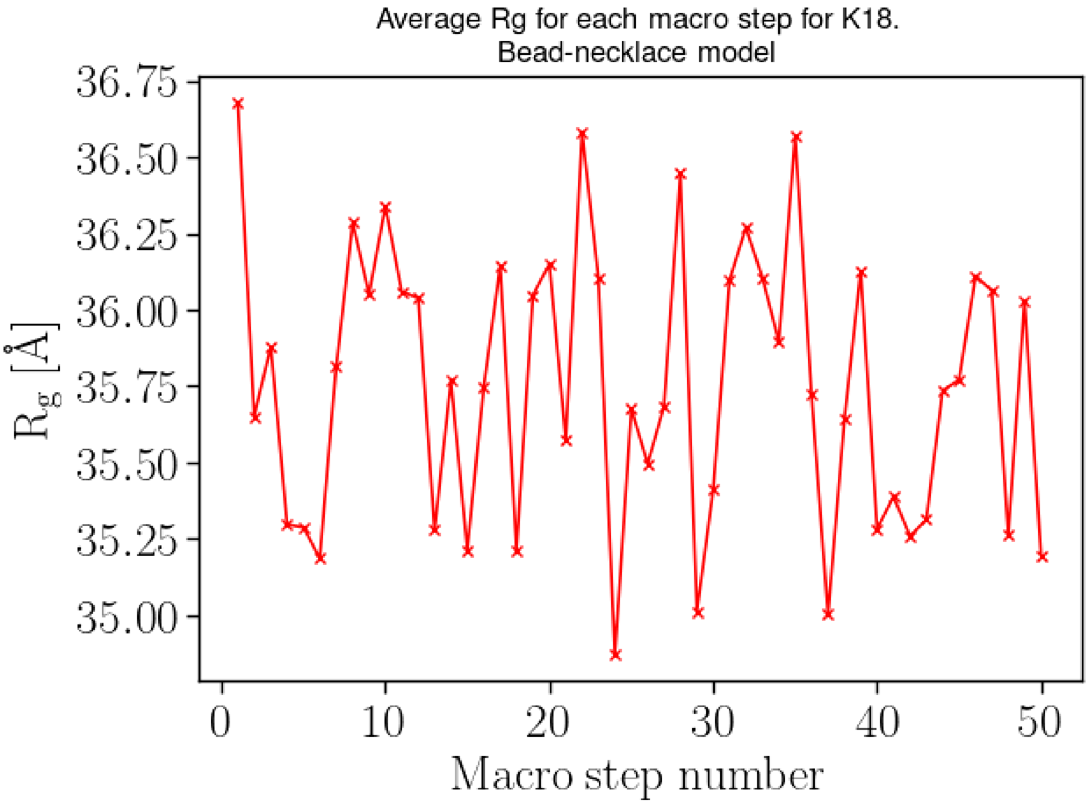

Figure S39: The average R_g for each macrostep (20 000 Monte Carlo steps) in the bead-necklace simulation. Only production run shown here.

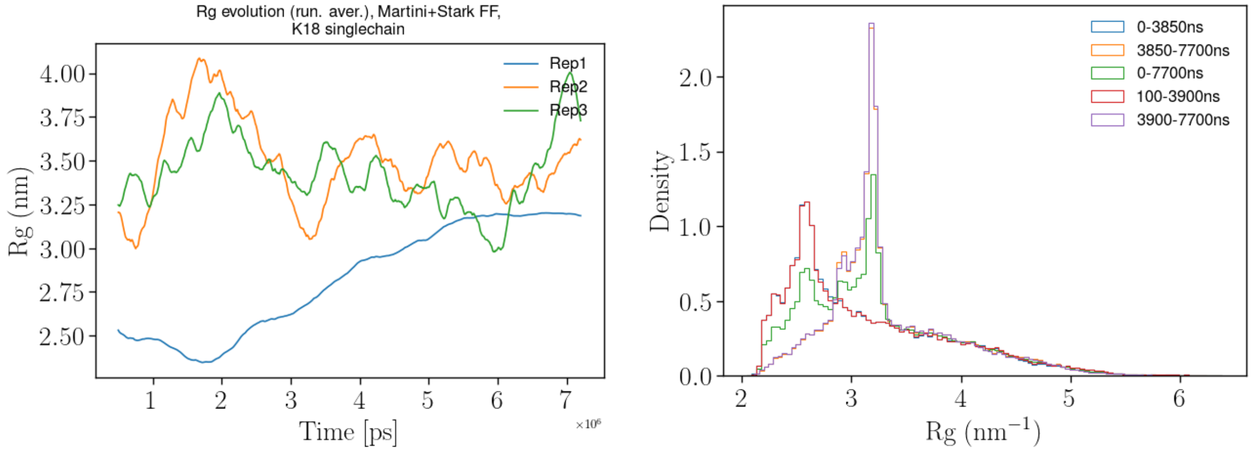

Figure S40: **Left:** Running average of R_g for the different replicate simulations of K18. **Right:** Distribution of R_g for all replicates combined, for different parts of the simulation (time-wise).

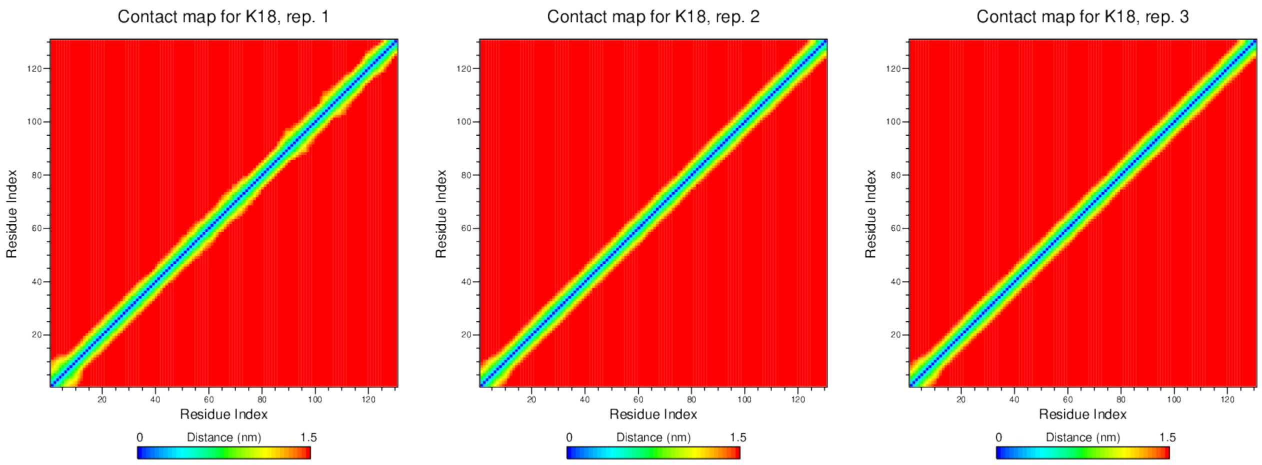

Figure S41: Contact maps from the MARTINI Stark simulation for K18. **Left:** Replicate 1. **Middle:** Replicate 2. **Right:** Replicate 3.

K17

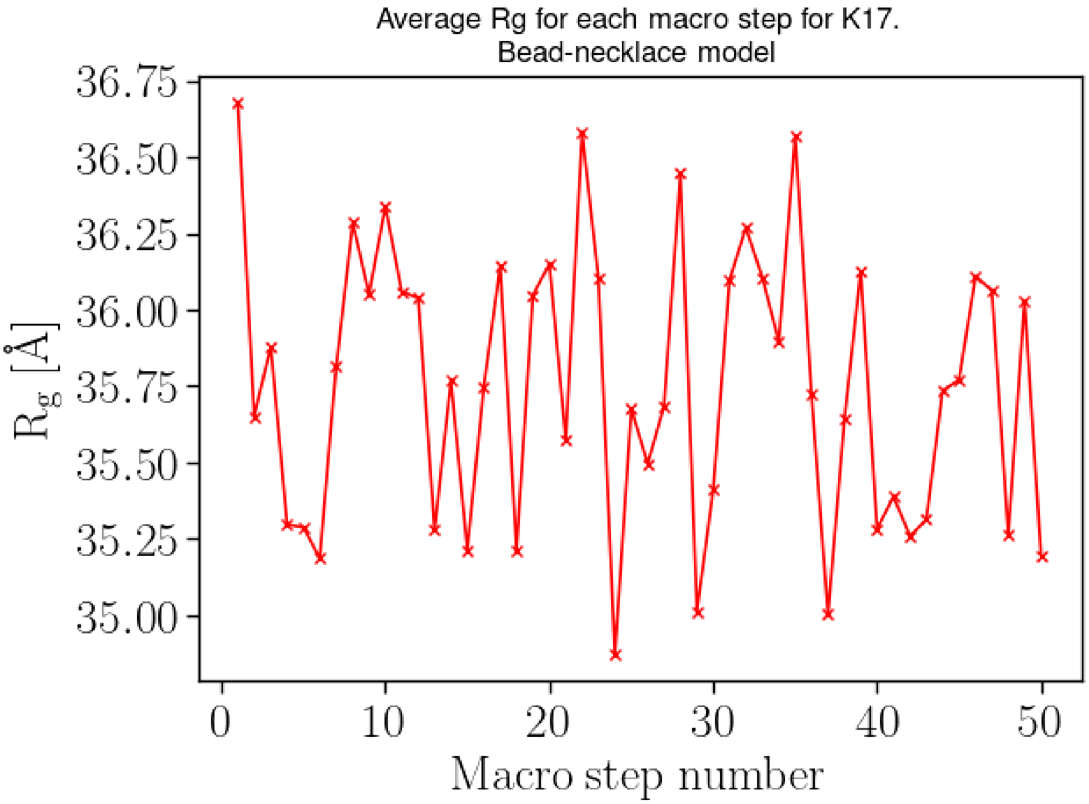

Figure S42: The average R_g for each macrostep (20 000 Monte Carlo steps) in the bead-necklace simulation. Only production run shown here.

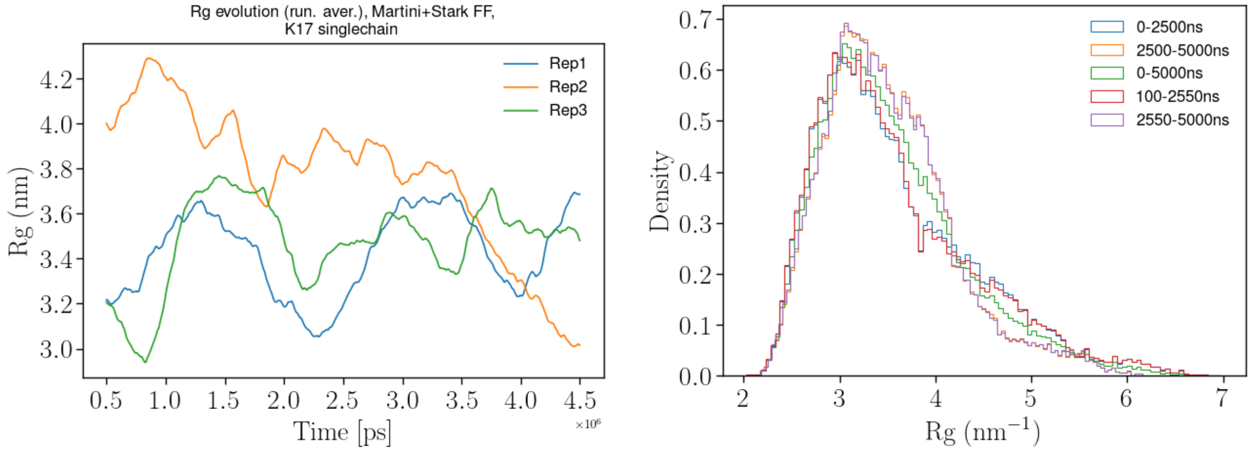

Figure S43: **Left:** Running average of R_g for the different replicate simulations of K17. **Right:** Distribution of R_g for all replicates combined, for different parts of the simulation (time-wise).

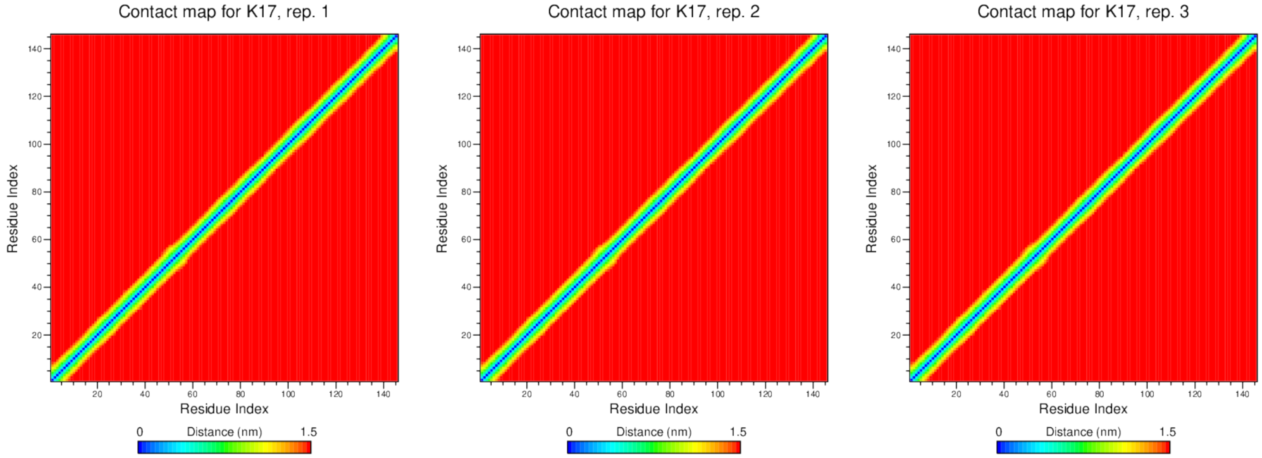

Figure S44: Contact maps from the MARTINI Stark simulation for K17. **Left:** Replicate 1. **Middle:** Replicate 2. **Right:** Replicate 3.

K10

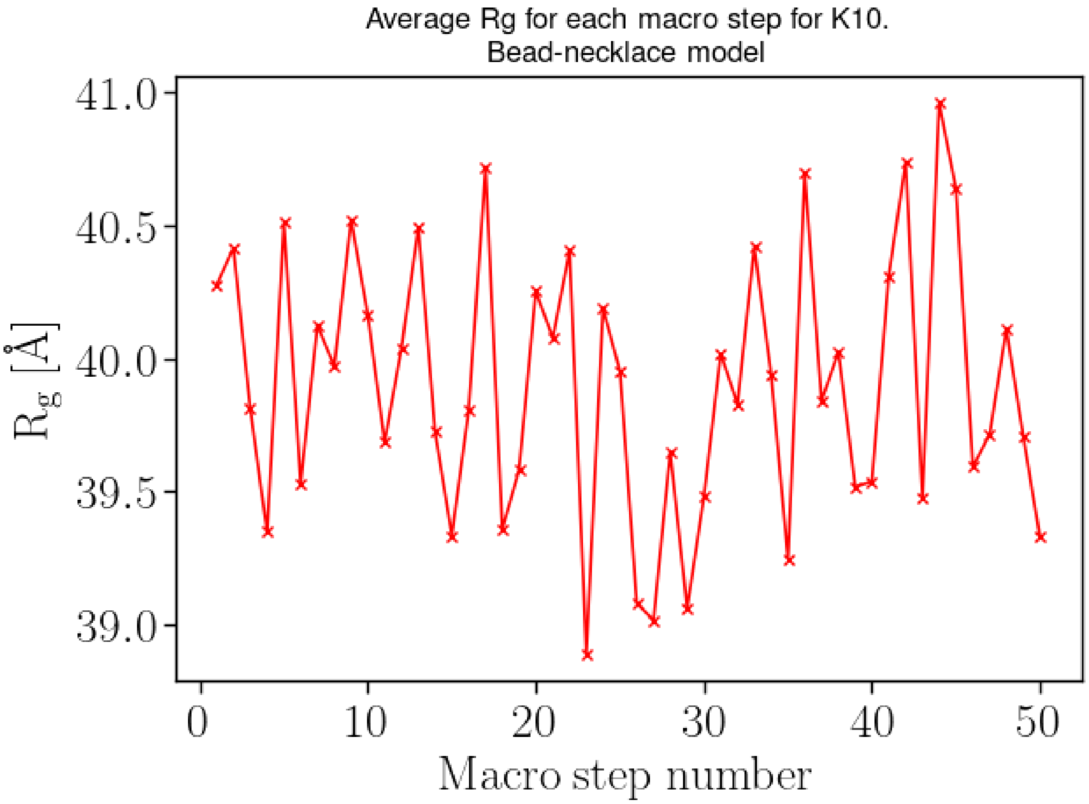

Figure S45: The average R_g for each macrostep (20 000 Monte Carlo steps) in the bead-necklace simulation. Only production run shown here.

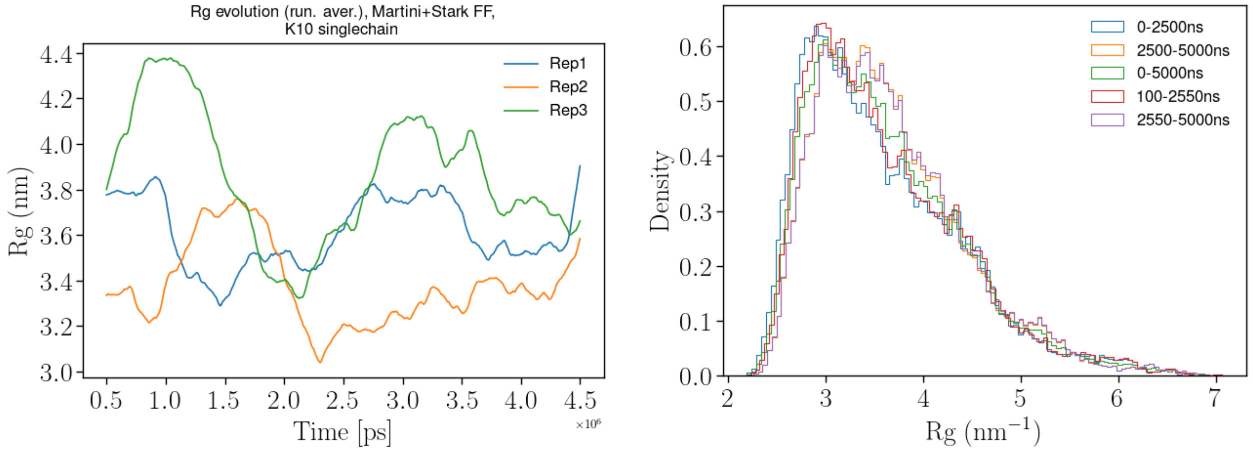

Figure S46: **Left:** Running average of R_g for the different replicate simulations of K10. **Right:** Distribution of R_g for all replicates combined, for different parts of the simulation (time-wise).

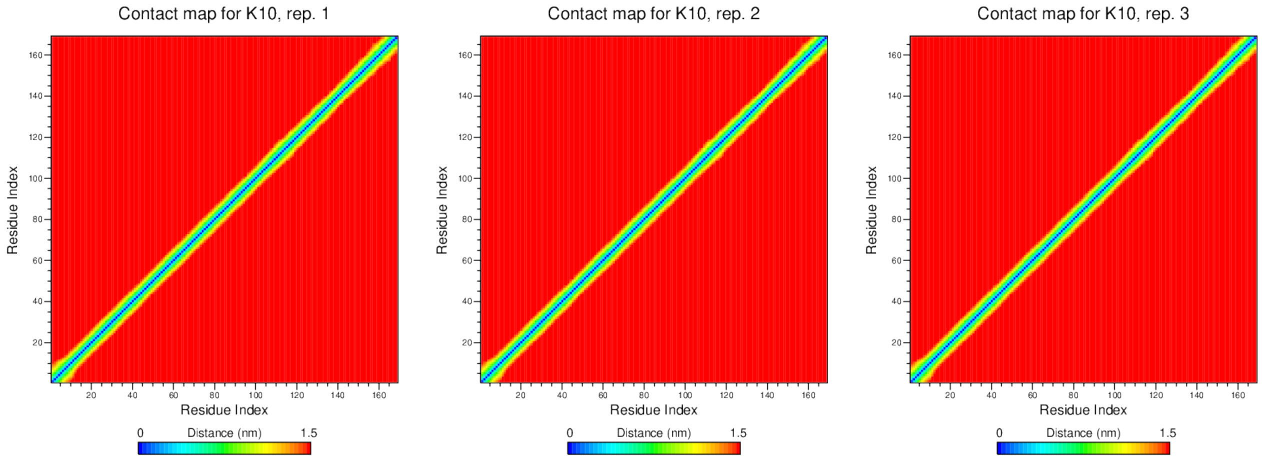

Figure S47: Contact maps from the MARTINI Stark simulation for K10. **Left:** Replicate 1. **Middle:** Replicate 2. **Right:** Replicate 3.

K27

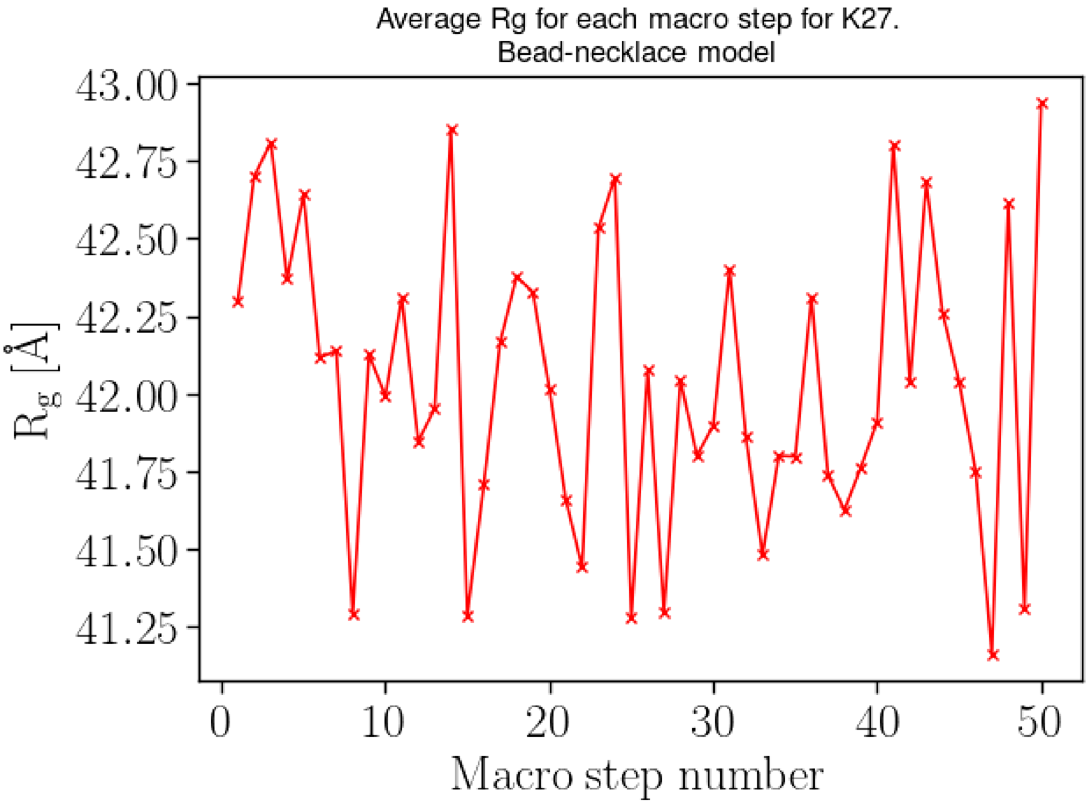

Figure S48: The average R_g for each macrostep (20 000 Monte Carlo steps) in the bead-necklace simulation. Only production run shown here.

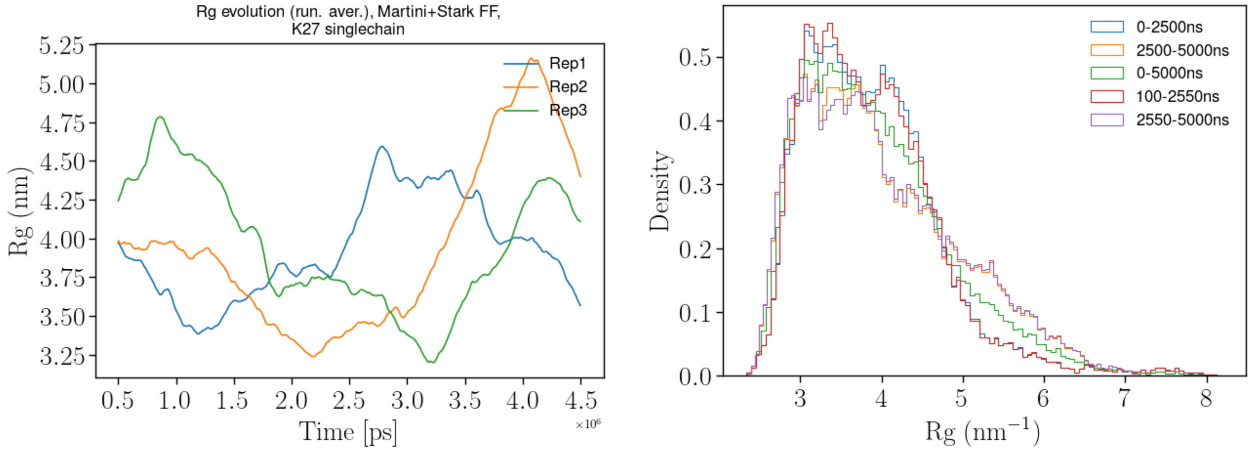

Figure S49: **Left:** Running average of R_g for the different replicate simulations of K27. **Right:** Distribution of R_g for all replicates combined, for different parts of the simulation (time-wise).

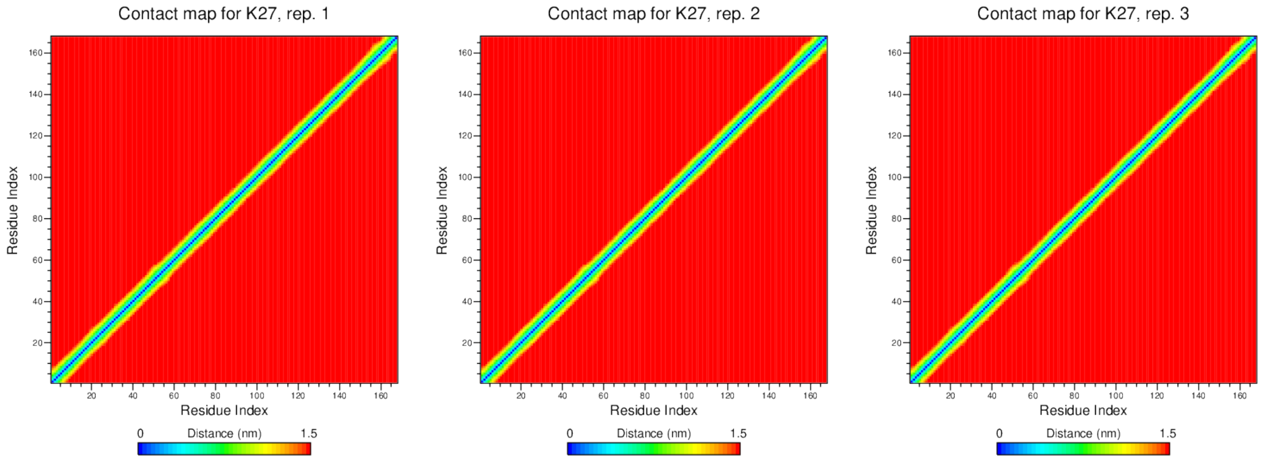

Figure S50: Contact maps from the MARTINI Stark simulation for K27. **Left:** Replicate 1. **Middle:** Replicate 2. **Right:** Replicate 3.

K16

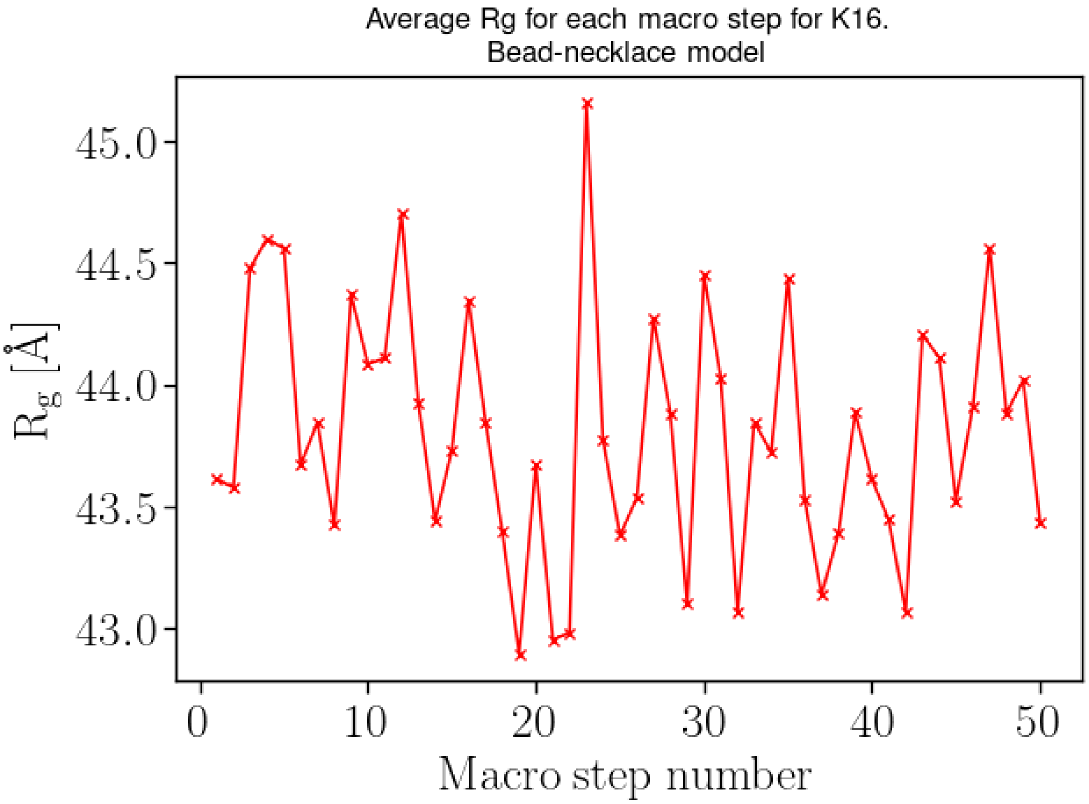

Figure S51: The average R_g for each macrostep (20 000 Monte Carlo steps) in the bead-necklace simulation. Only production run shown here.

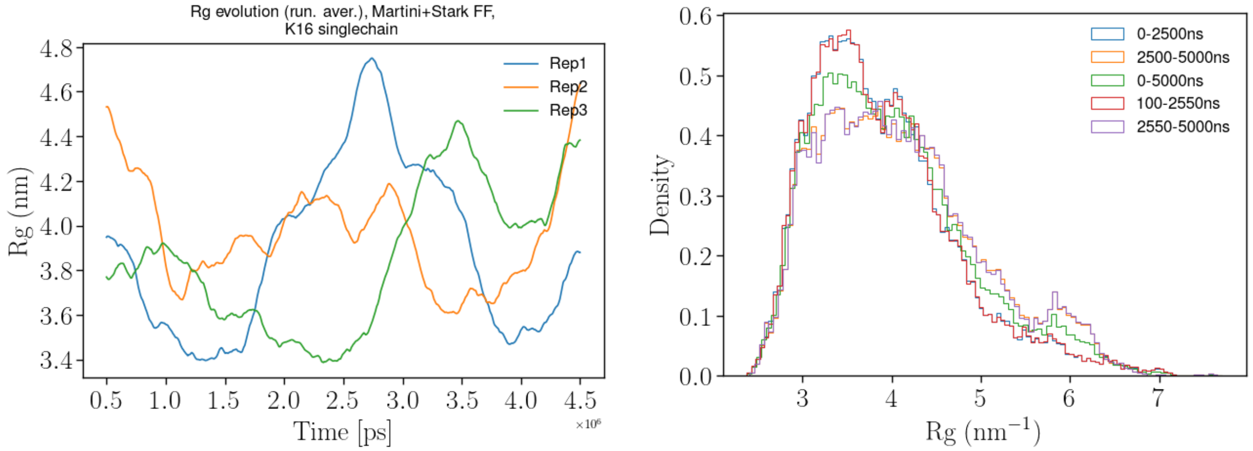

Figure S52: **Left:** Running average of R_g for the different replicate simulations of K16. **Right:** Distribution of R_g for all replicates combined, for different parts of the simulation (time-wise).

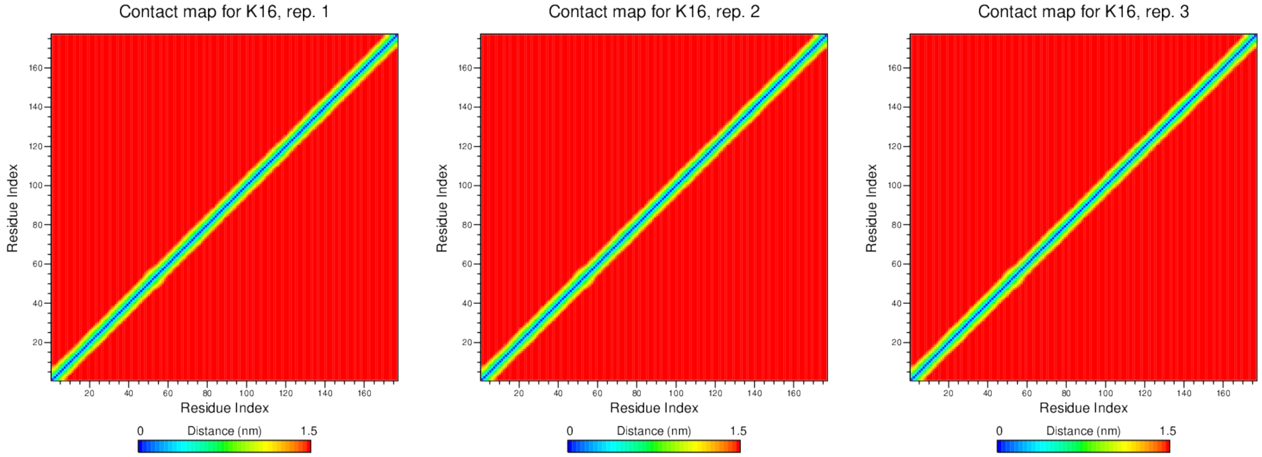

Figure S53: Contact maps from the MARTINI Stark simulation for K16. **Left:** Replicate 1. **Middle:** Replicate 2. **Right:** Replicate 3.

K25

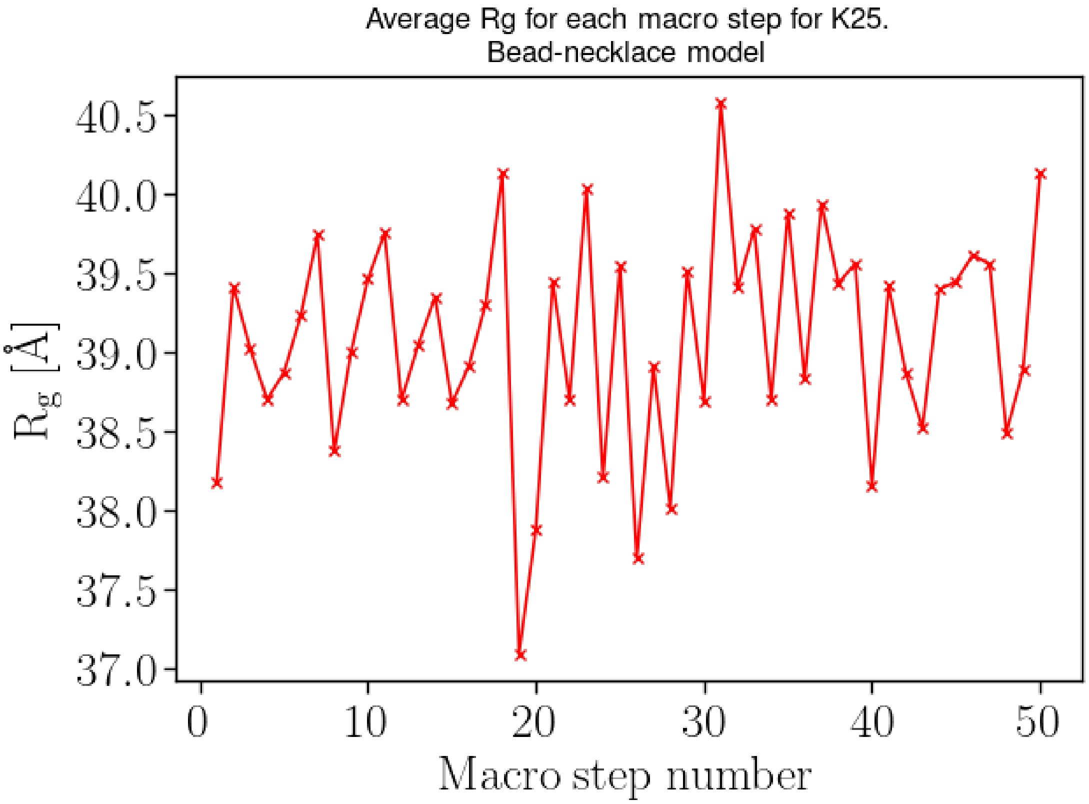

Figure S54: The average R_g for each macrostep (20 000 Monte Carlo steps) in the bead-necklace simulation. Only production run shown here.

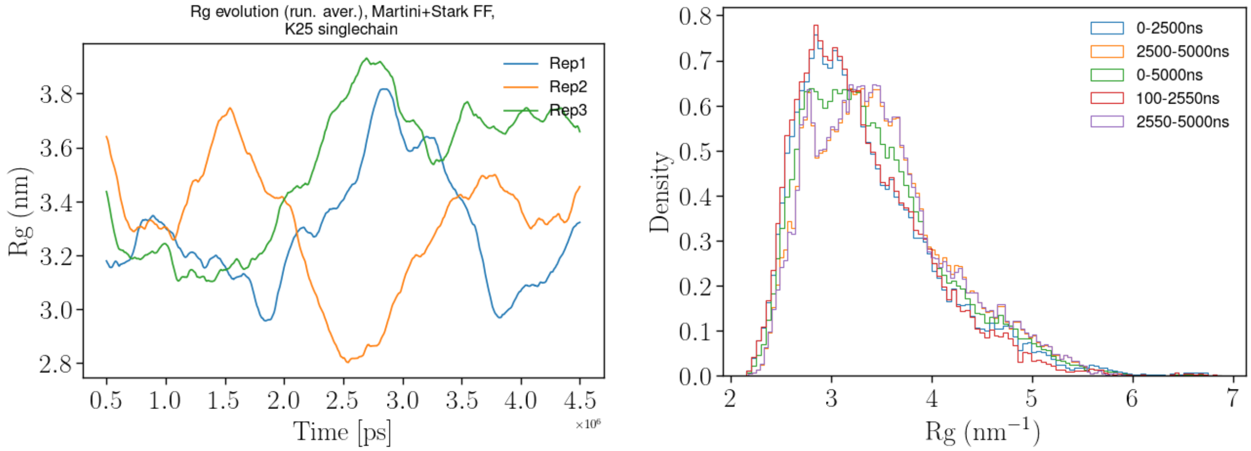

Figure S55: **Left:** Running average of R_g for the different replicate simulations of K25. **Right:** Distribution of R_g for all replicates combined, for different parts of the simulation (time-wise).

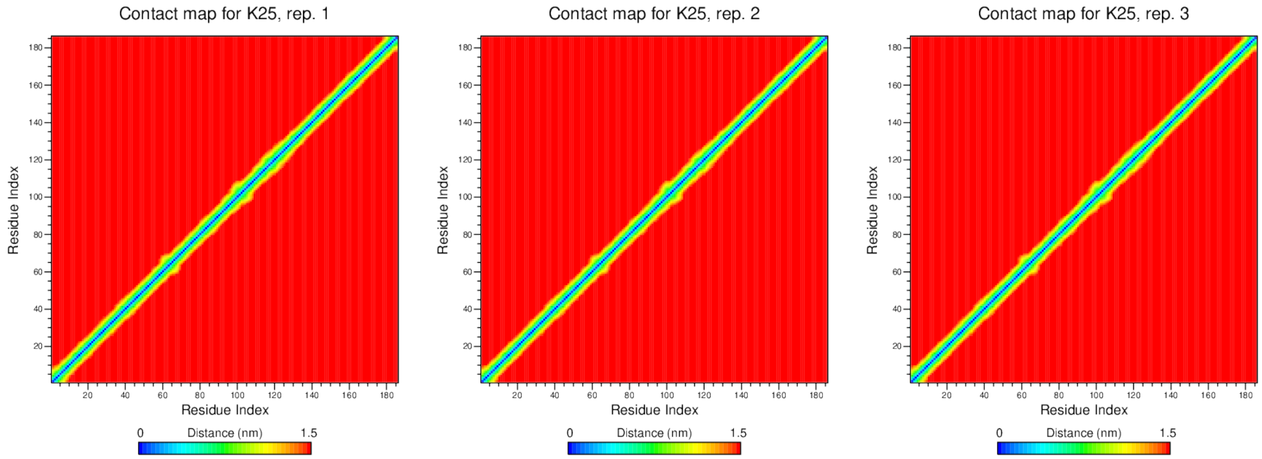

Figure S56: Contact maps from the MARTINI Stark simulation for K25. **Left:** Replicate 1. **Middle:** Replicate 2. **Right:** Replicate 3.

K32

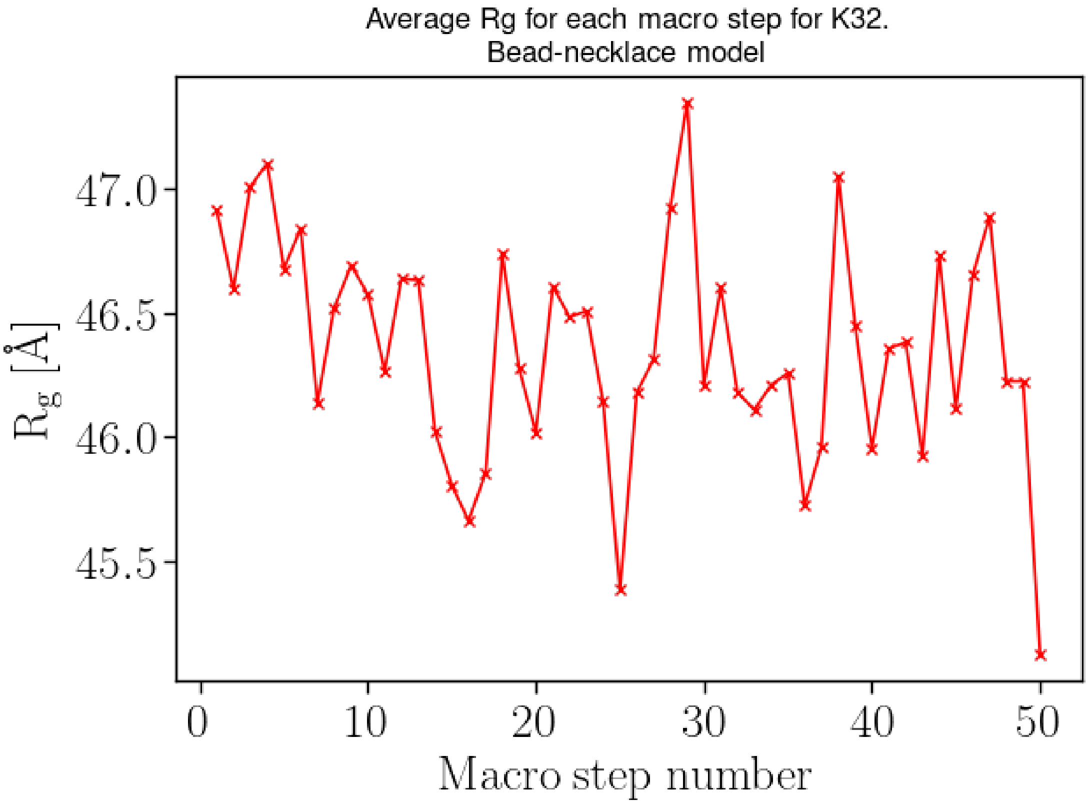

Figure S57: The average R_g for each macrostep (20 000 Monte Carlo steps) in the bead-necklace simulation. Only production run shown here.

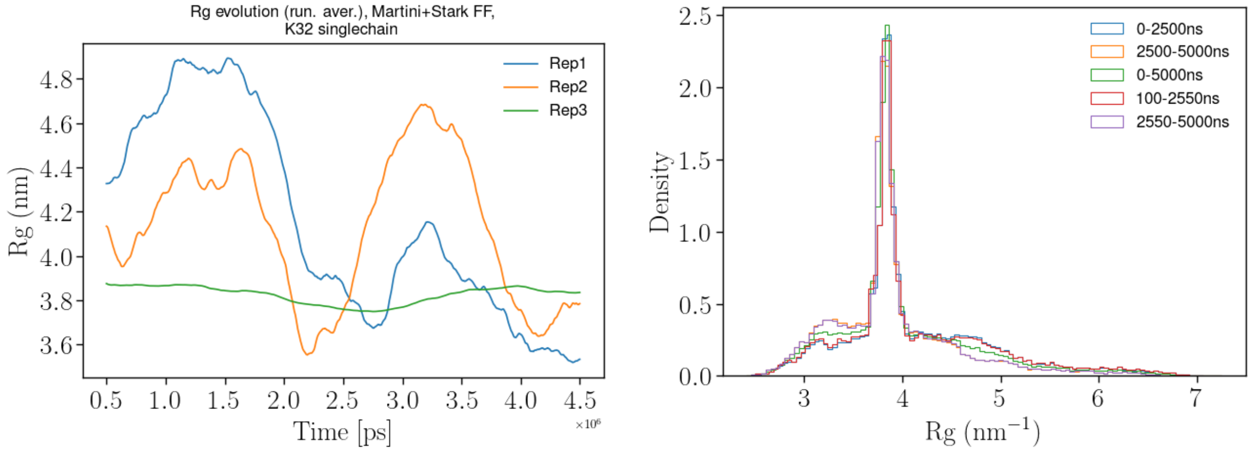

Figure S58: **Left:** Running average of R_g for the different replicate simulations of K32. **Right:** Distribution of R_g for all replicates combined, for different parts of the simulation (time-wise).

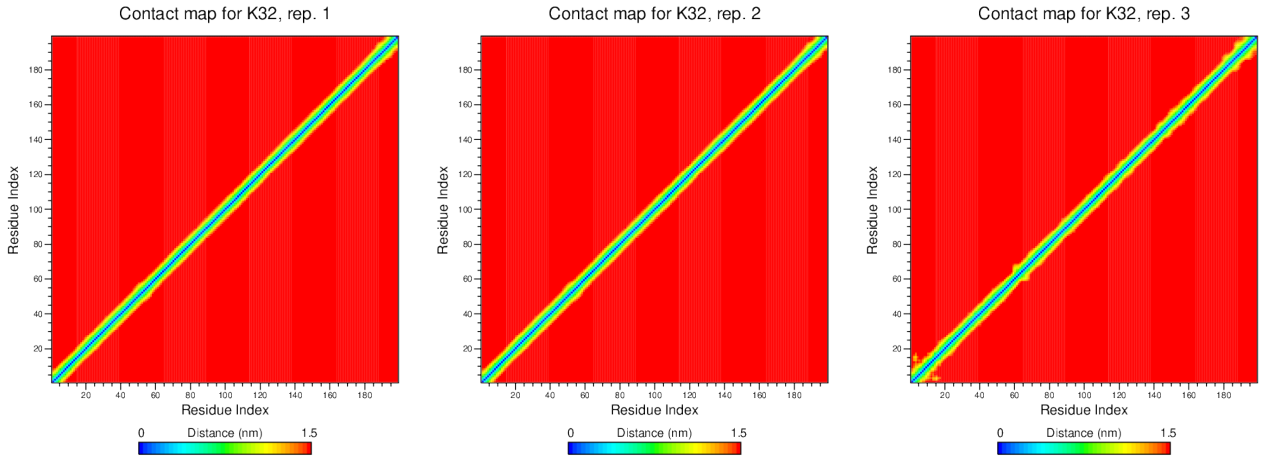

Figure S59: Contact maps from the MARTINI Stark simulation for K32. **Left:** Replicate 1. **Middle:** Replicate 2. **Right:** Replicate 3.

K23

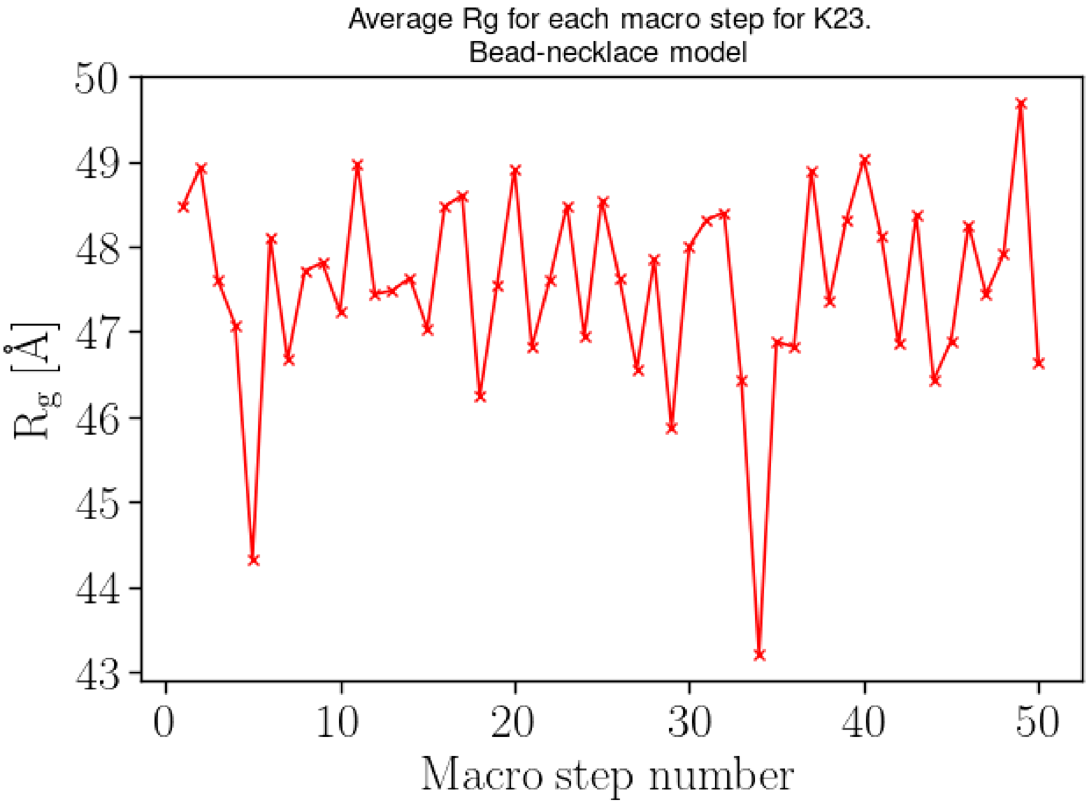

Figure S60: The average R_g for each macrostep (20 000 Monte Carlo steps) in the bead-necklace simulation. Only production run shown here.

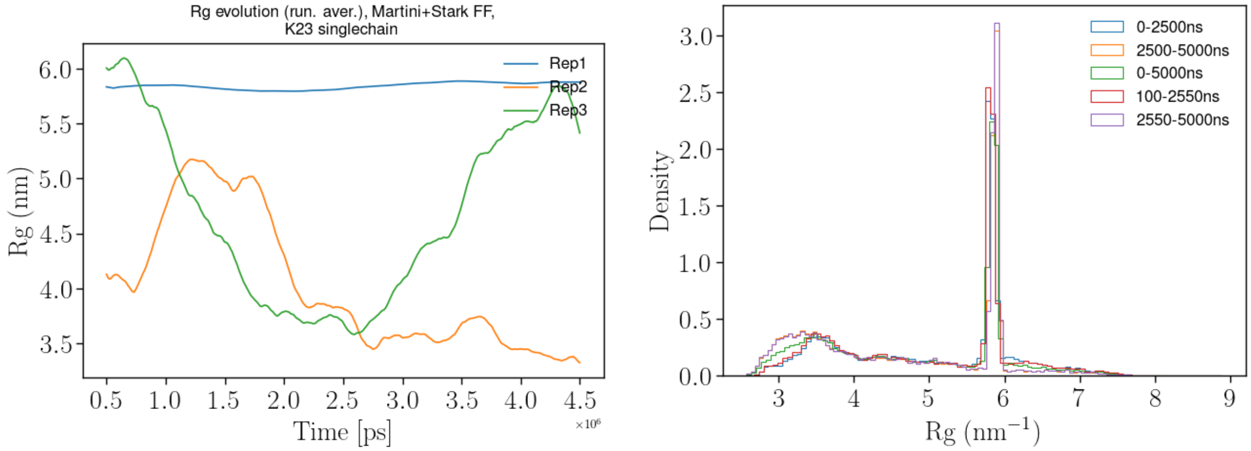

Figure S61: **Left:** Running average of R_g for the different replicate simulations of K23. **Right:** Distribution of R_g for all replicates combined, for different parts of the simulation (time-wise).

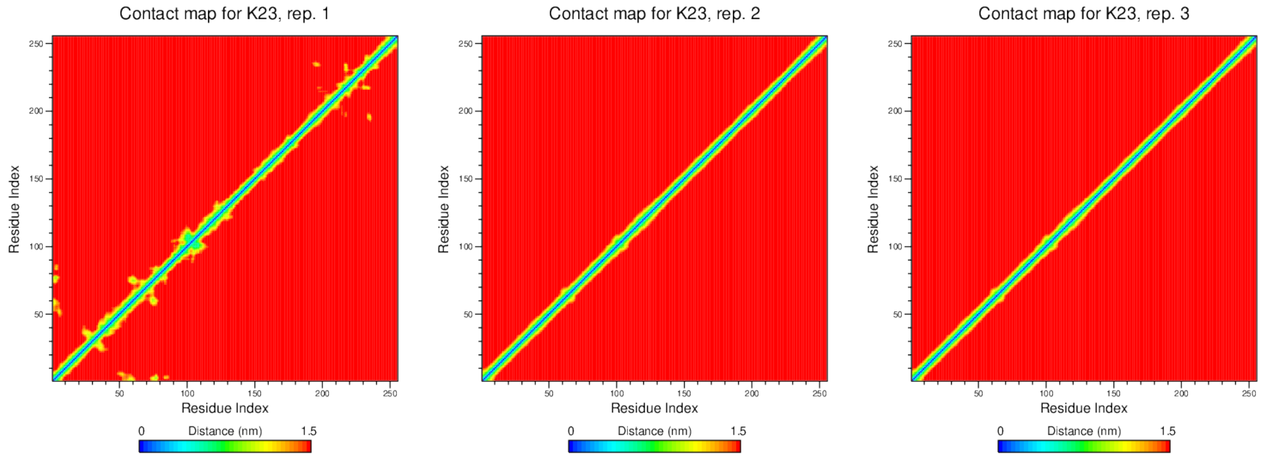

Figure S62: Contact maps from the MARTINI Stark simulation for K23. **Left:** Replicate 1. **Middle:** Replicate 2. **Right:** Replicate 3.

K44

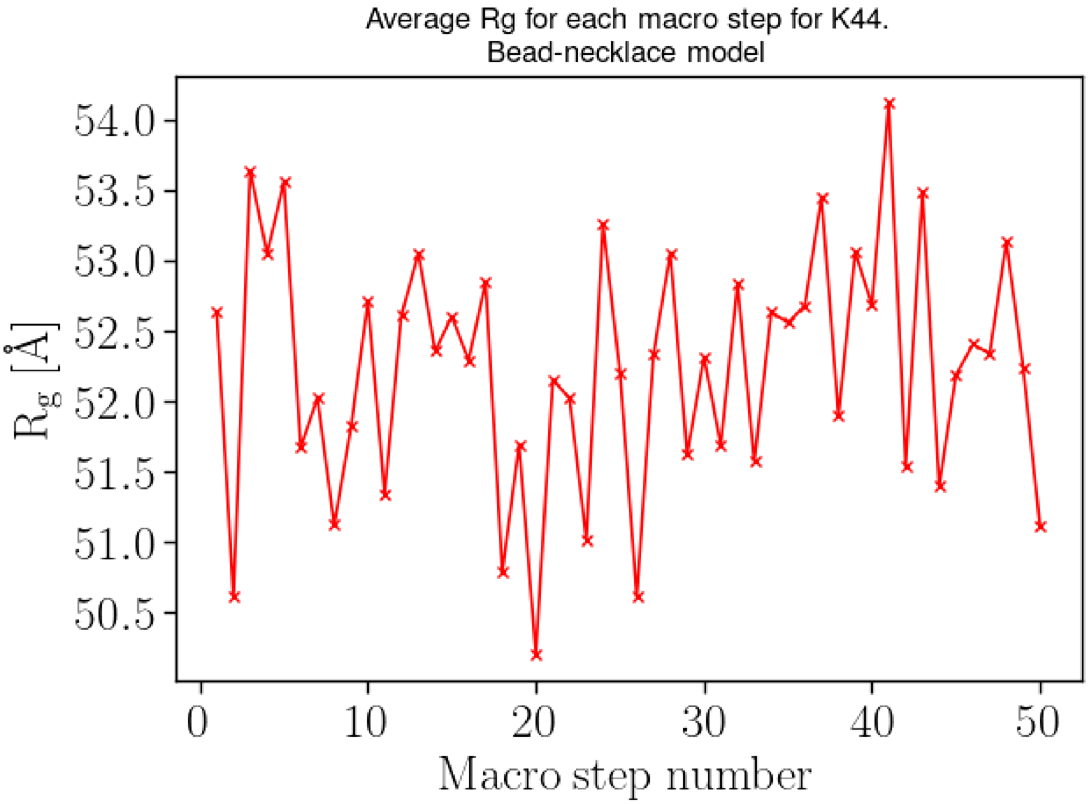

Figure S63: The average R_g for each macrostep (20 000 Monte Carlo steps) in the bead-necklace simulation. Only production run shown here.

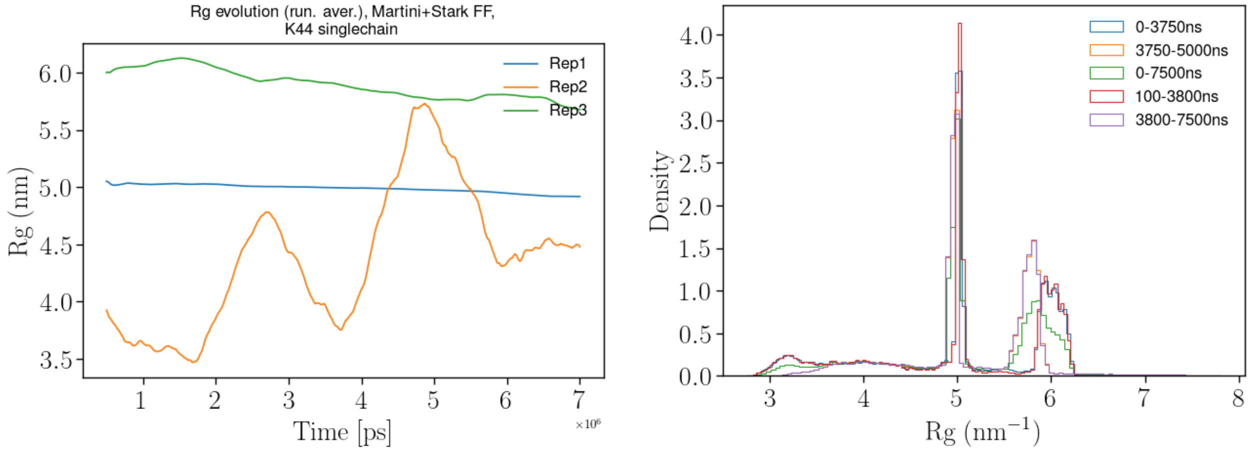

Figure S64: **Left:** Running average of R_g for the different replicate simulations of K44. **Right:** Distribution of R_g for all replicates combined, for different parts of the simulation (time-wise).

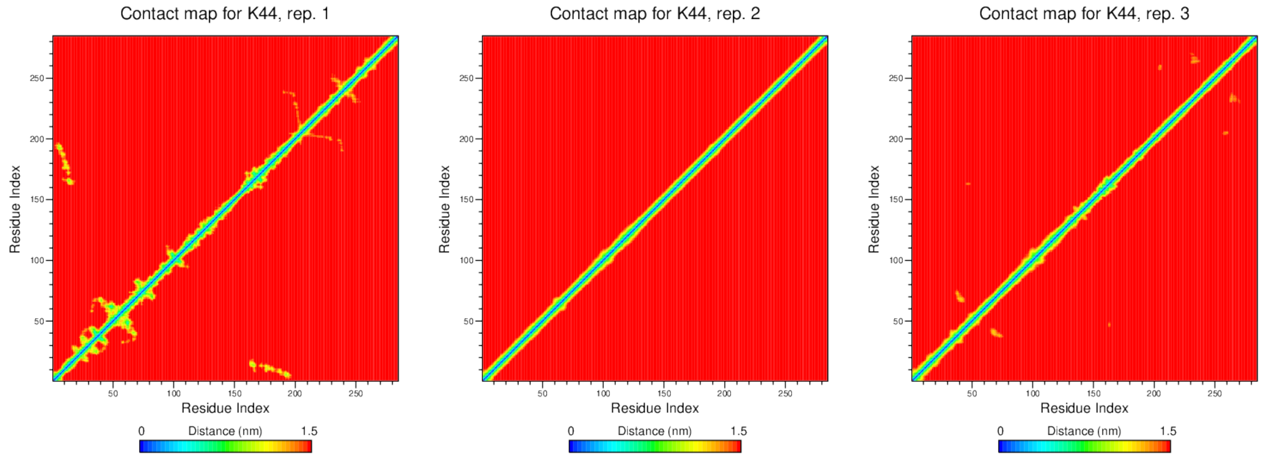

Figure S65: Contact maps from the MARTINI Stark simulation for K44. **Left:** Replicate 1. **Middle:** Replicate 2. **Right:** Replicate 3.

hTau23

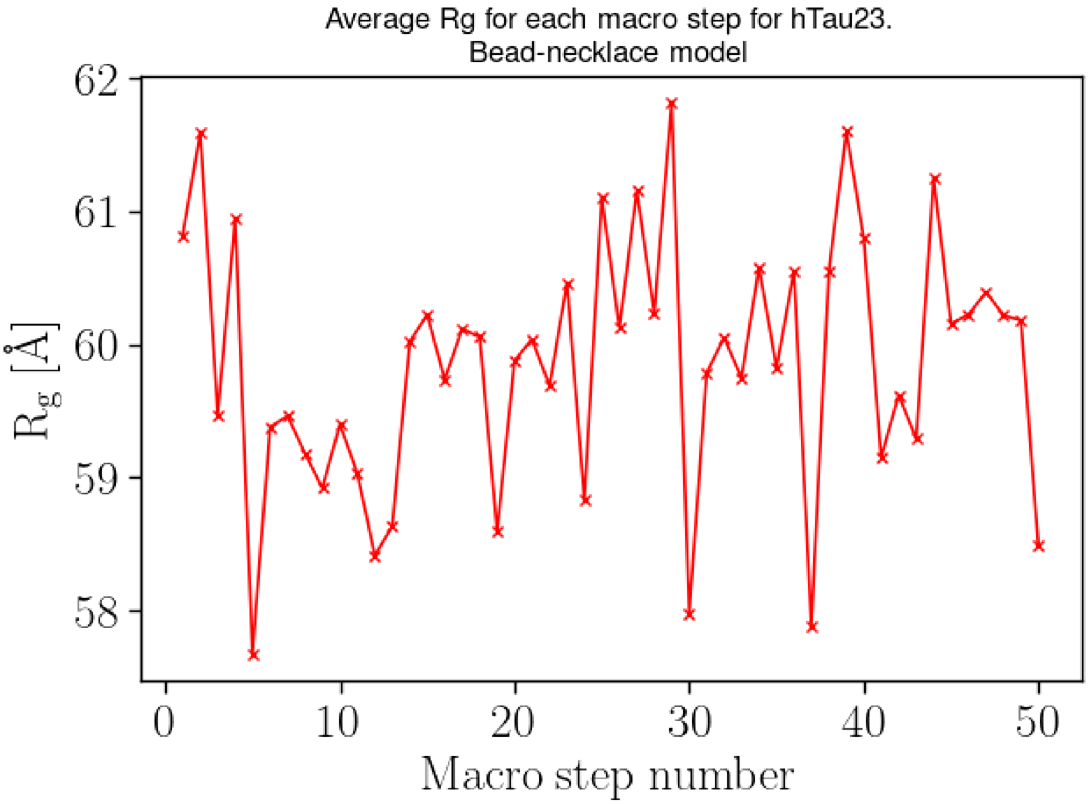

Figure S66: The average R_g for each macrostep (20 000 Monte Carlo steps) in the bead-necklace simulation. Only production run shown here.

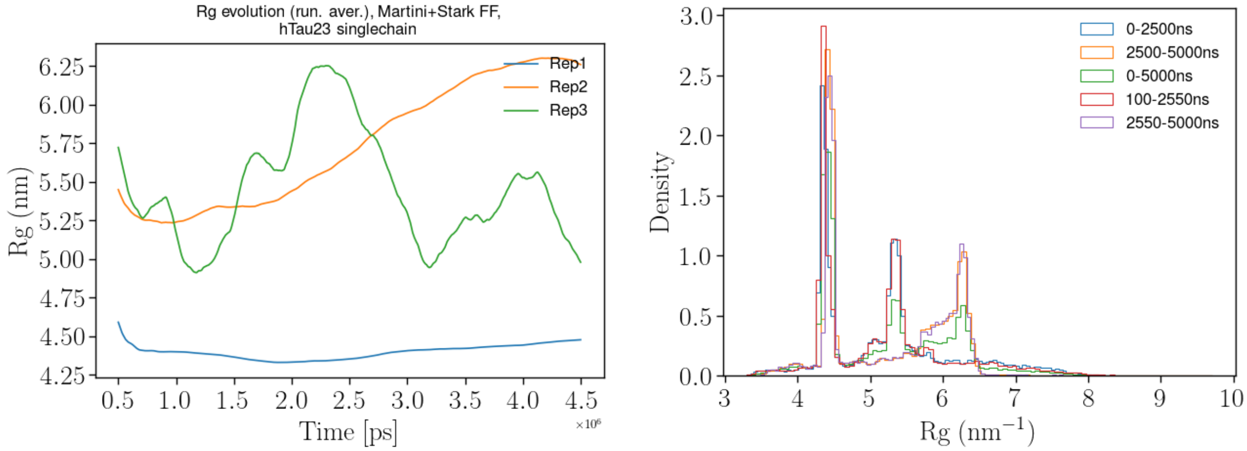

Figure S67: **Left:** Running average of R_g for the different replicate simulations of hTau23. **Right:** Distribution of R_g for all replicates combined, for different parts of the simulation (time-wise).

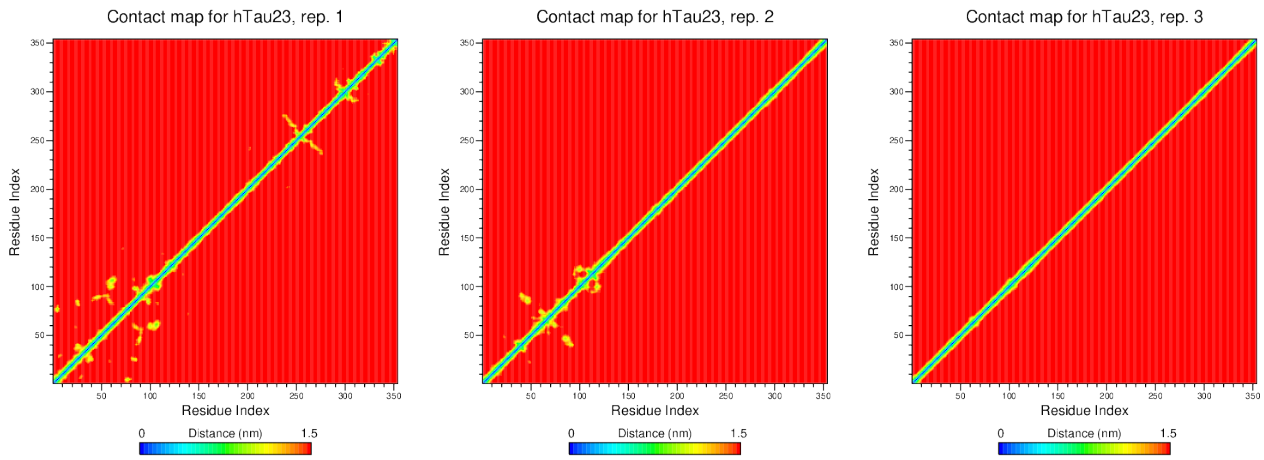

Figure S68: Contact maps from the MARTINI Stark simulation for hTau23. **Left:** Replicate 1. **Middle:** Replicate 2. **Right:** Replicate 3.

hTau40

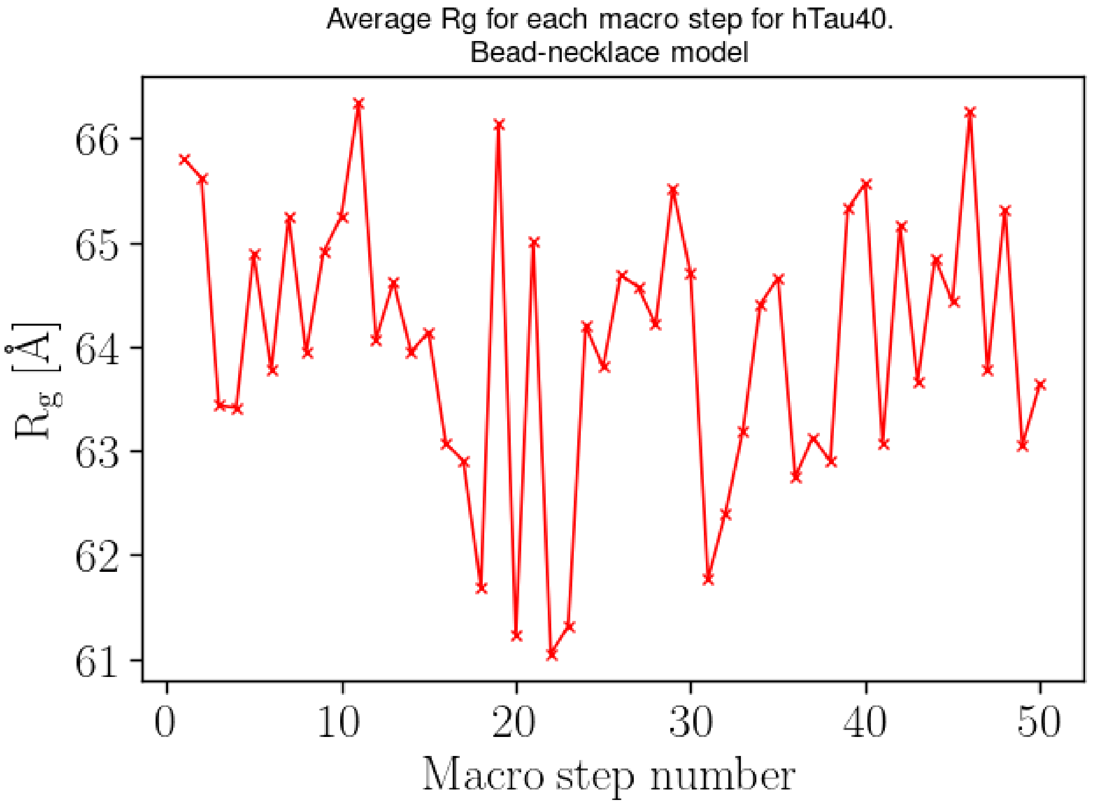

Figure S69: The average R_g for each macrostep (20 000 Monte Carlo steps) in the bead-necklace simulation. Only production run shown here.

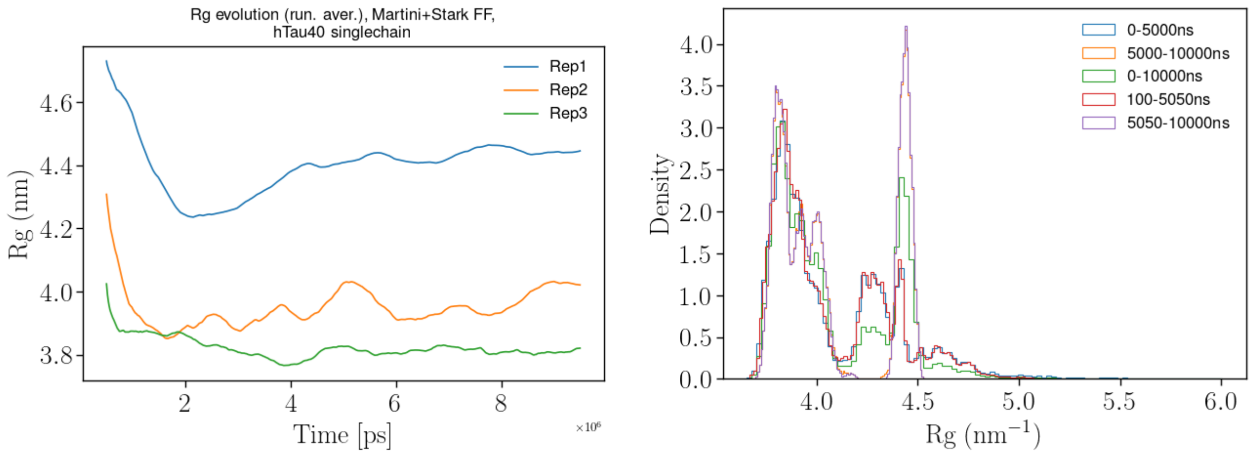

Figure S70: **Left:** Running average of R_g for the different replicate simulations of hTau40. **Right:** Distribution of R_g for all replicates combined, for different parts of the simulation (time-wise).

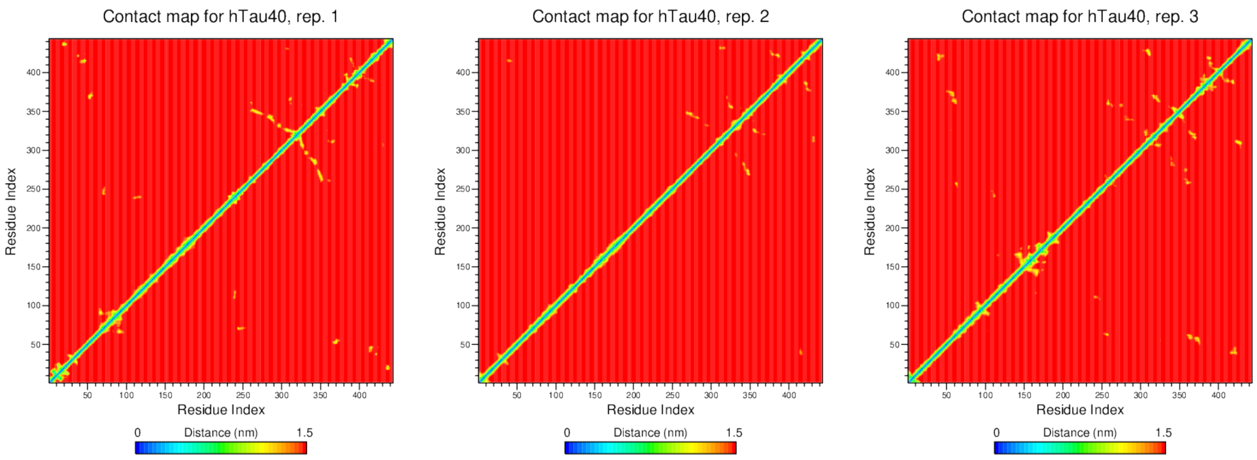

Figure S71: Contact maps from the MARTINI Stark simulation for hTau40. **Left:** Replicate 1. **Middle:** Replicate 2. **Right:** Replicate 3.

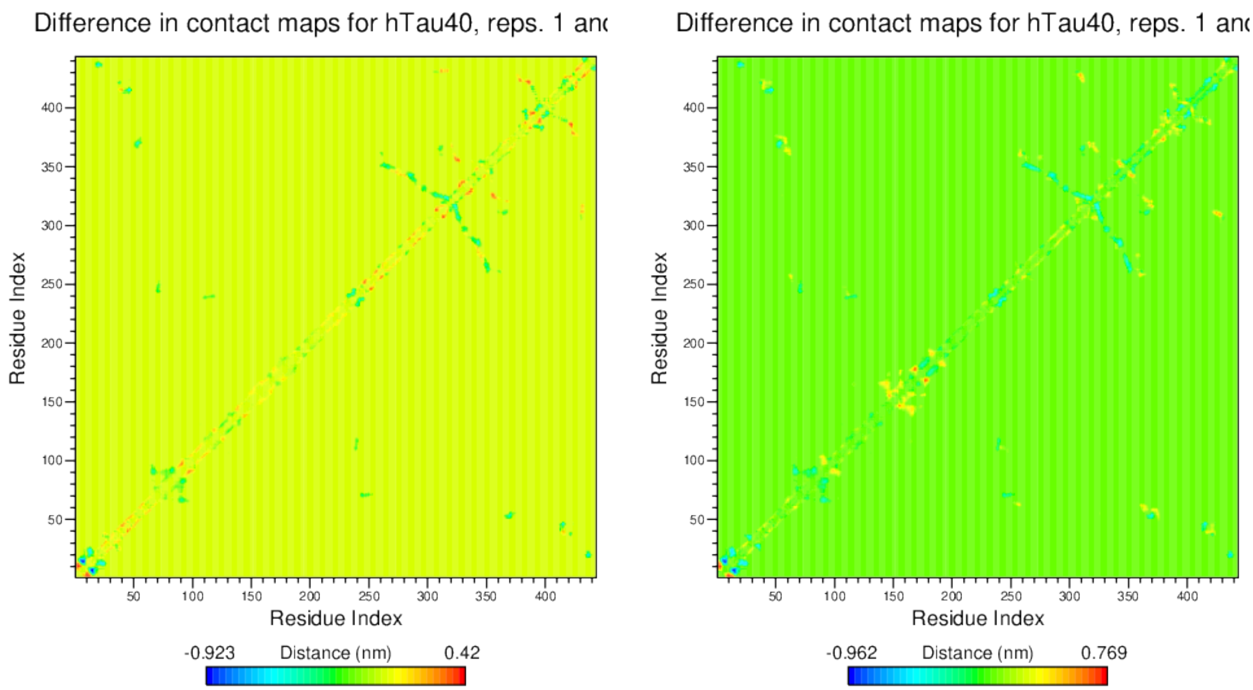

Figure S72: Difference in contact maps from the MARTINI Stark simulation for hTau40. **Left:** Difference between replicate 1 and 2. **Right:** Difference between replicate 1 and 3.

KEIF

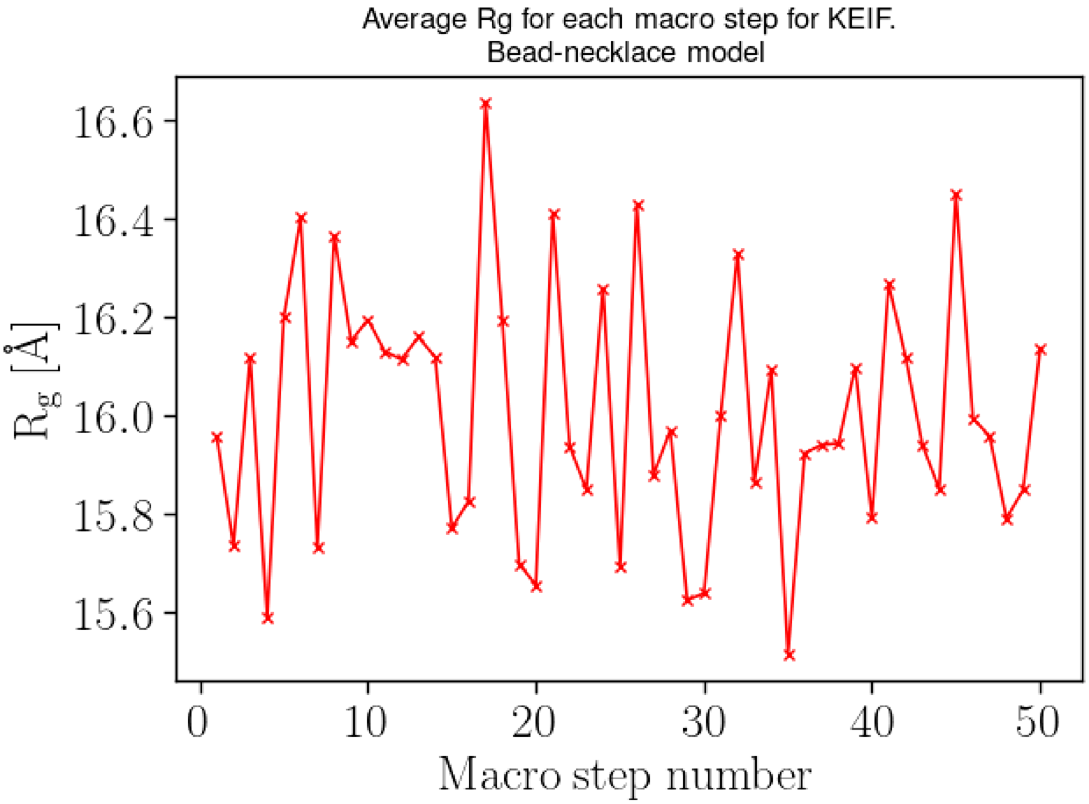

Figure S73: The average R_g for each macrostep (20 000 Monte Carlo steps) in the bead-necklace simulation. Only production run shown here.

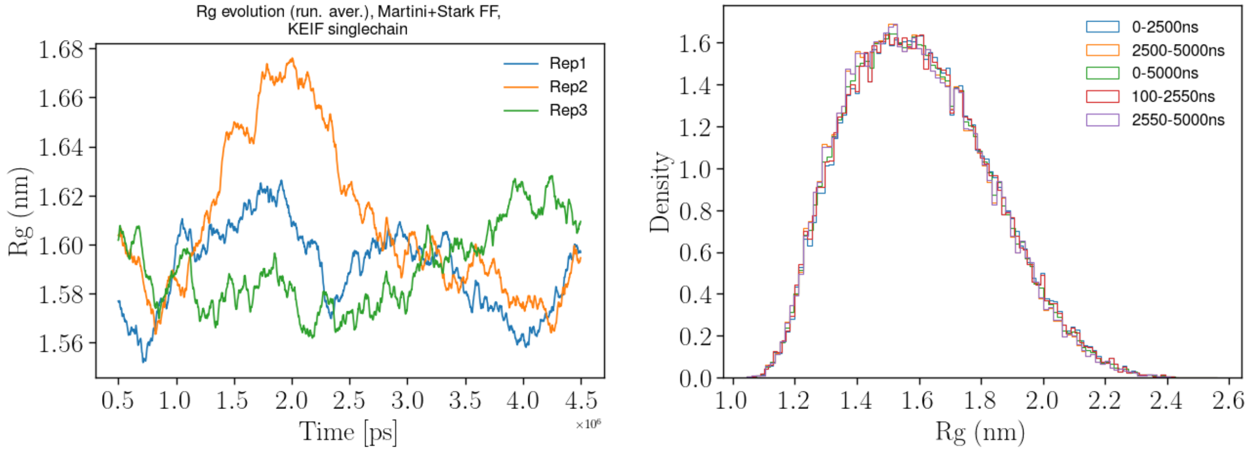

Figure S74: **Left:** Running average of R_g for the different replicate simulations of KEIF. **Right:** Distribution of R_g for all replicates combined, for different parts of the simulation (time-wise).

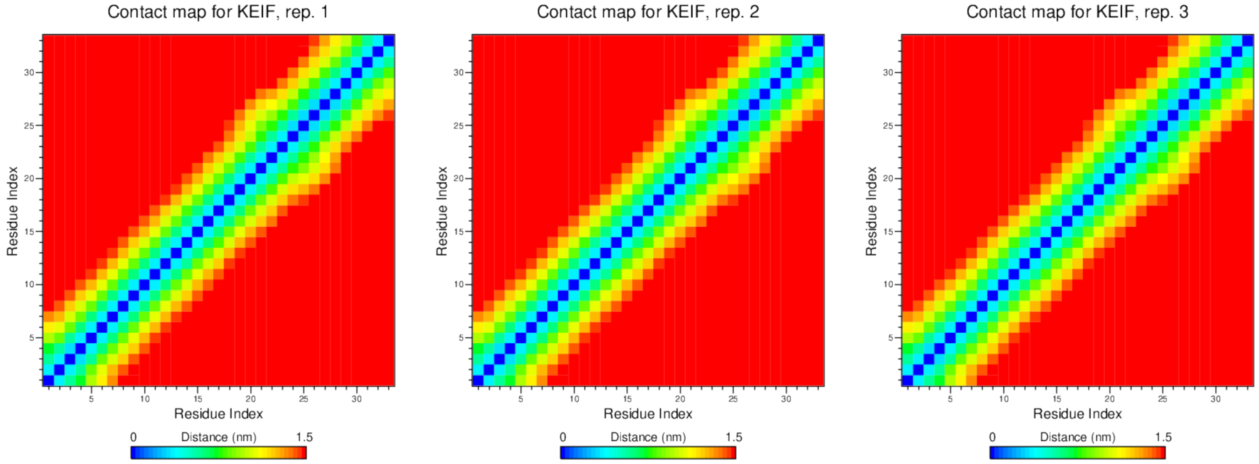

Figure S75: Contact maps from the MARTINI Stark simulation for KEIF. **Left:** Replicate 1. **Middle:** Replicate 2. **Right:** Replicate 3.

(Histatin 5)$_2$

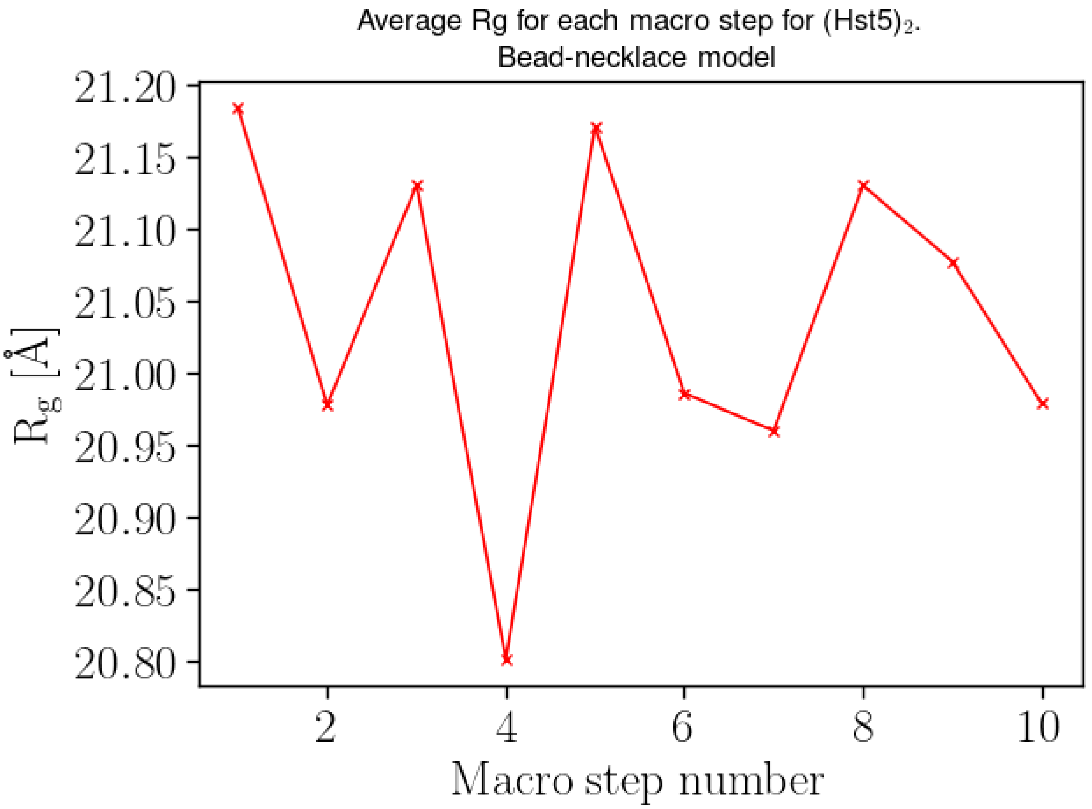

Figure S76: The average R_g for each macrostep (200 000 Monte Carlo steps) in the bead-necklace simulation. Only production run shown here.

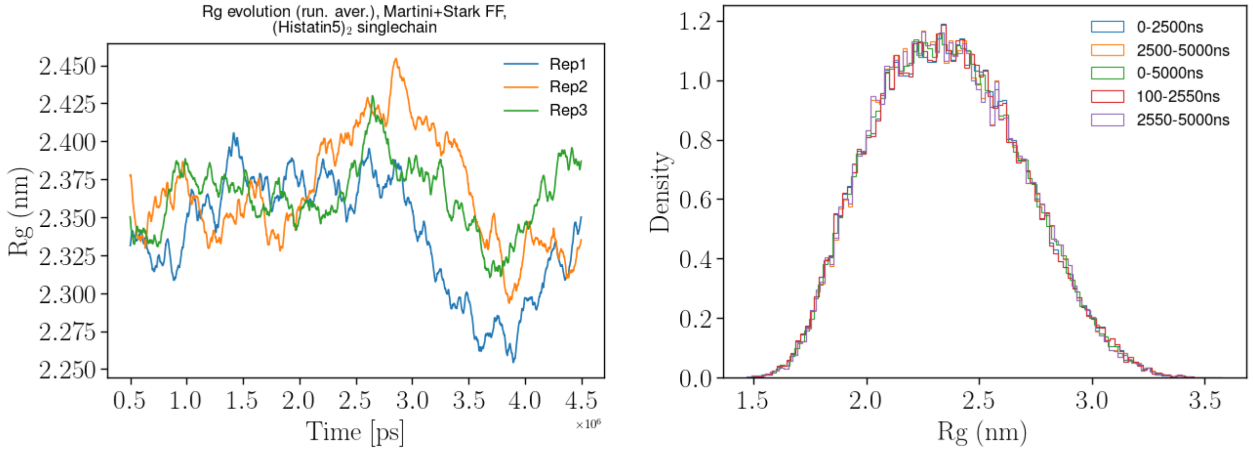

Figure S77: **Left:** Running average of R_g for the different replicate simulations of (Histatin 5)$_2$. **Right:** Distribution of R_g for all replicates combined, for different parts of the simulation (time-wise).

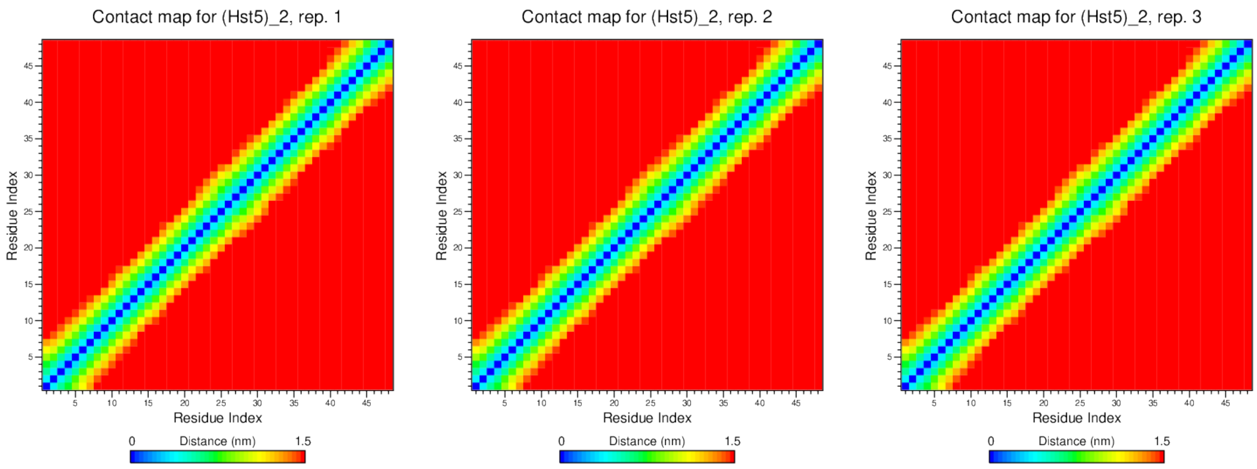

Figure S78: Contact maps from the MARTINI Stark simulation for (Hst5)$_2$. **Left:** Replicate 1. **Middle:** Replicate 2. **Right:** Replicate 3.

4 Notes on experimental data

In Table 1, we reproduce the values reported by Baul *et al.*[1] However, there are some discrepancies comparing the original data with this data:

In the original citation,[2] SH4-UD was reported to have R_g of 31.1 ± 0.3 Å using the P(r) method, and 28.2 ± 0.4 Å using the Guinier approximation.

Prothymosin α was reported by Baul *et al.* to have a radius of gyration of 37.9 Å. However, we find the originally cited article[3] to report a radius of gyration of 37.8 Å. A larger difference between the table of Baul *et al.* and cited literature is found in the case of ERM TADn, where the table lists R_g as 38.1 Å while the cited literature actually reports R_g of 39.6 Å, using the Guinier approximation.[4]

5 Bead-necklace model RDF of counter ions to protein chain

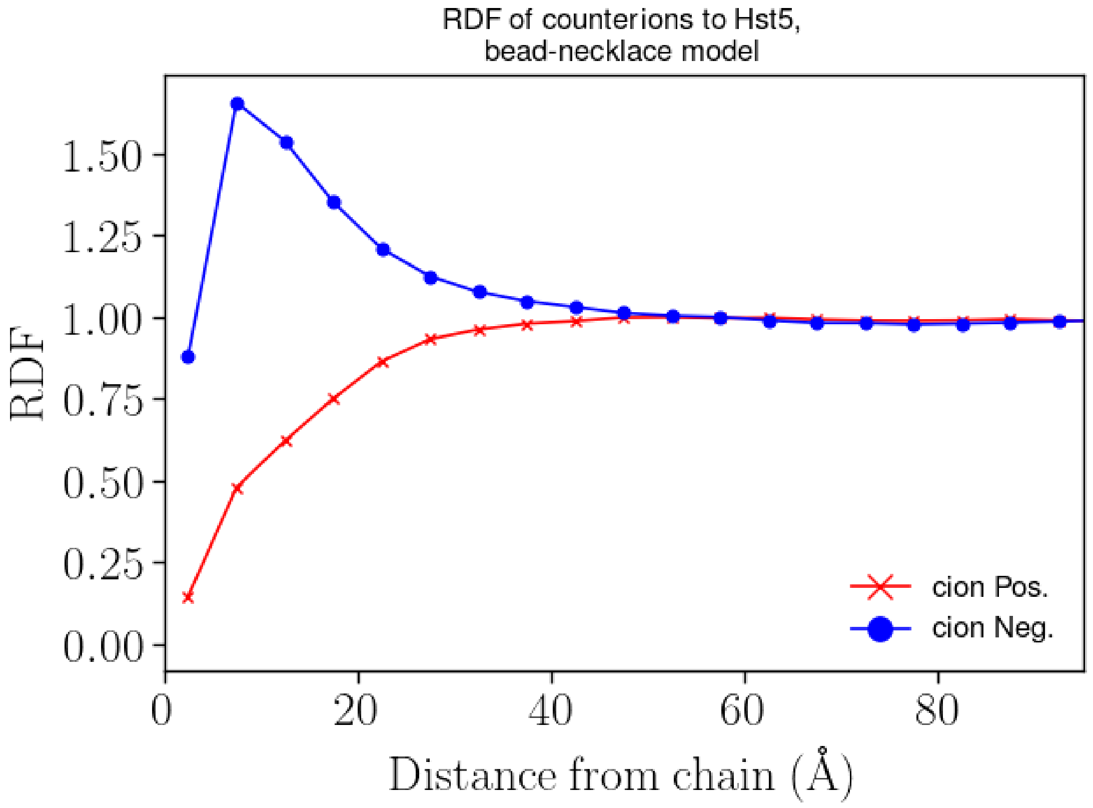

Figure S79: Radial distribution function (RDF) for the counter ions towards the protein chain, Hst5.

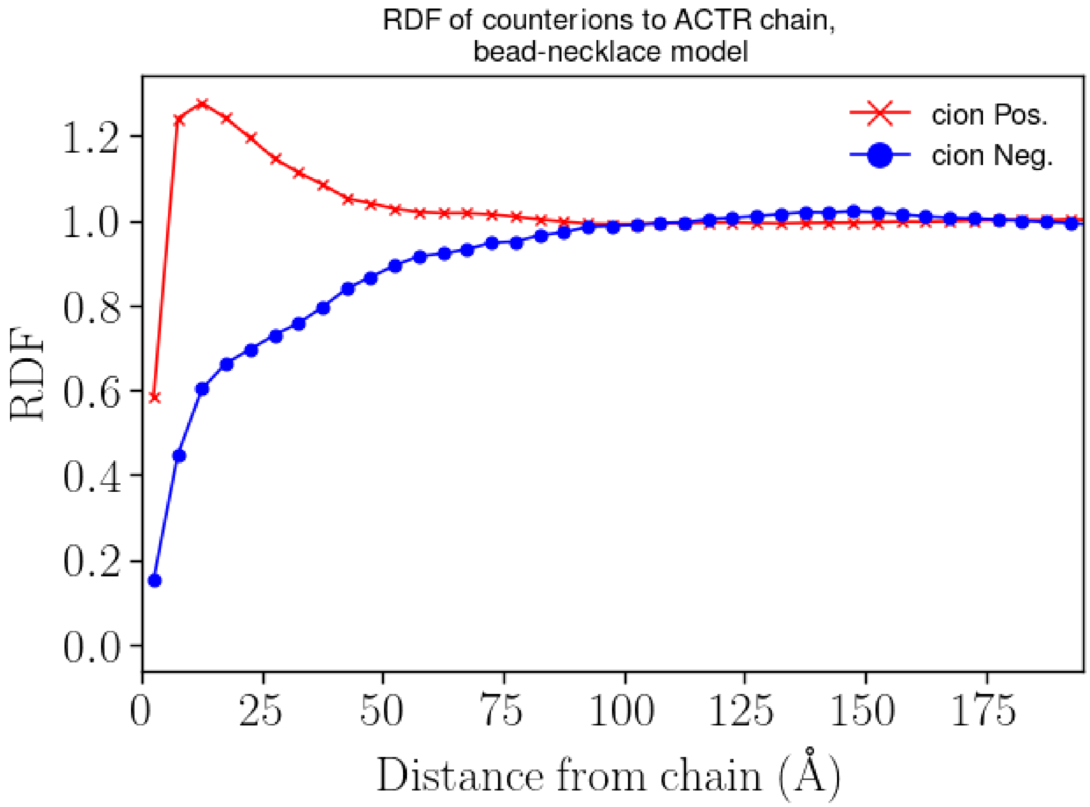

Figure S80: Radial distribution function (RDF) for the counter ions towards the protein chain, ACTR.

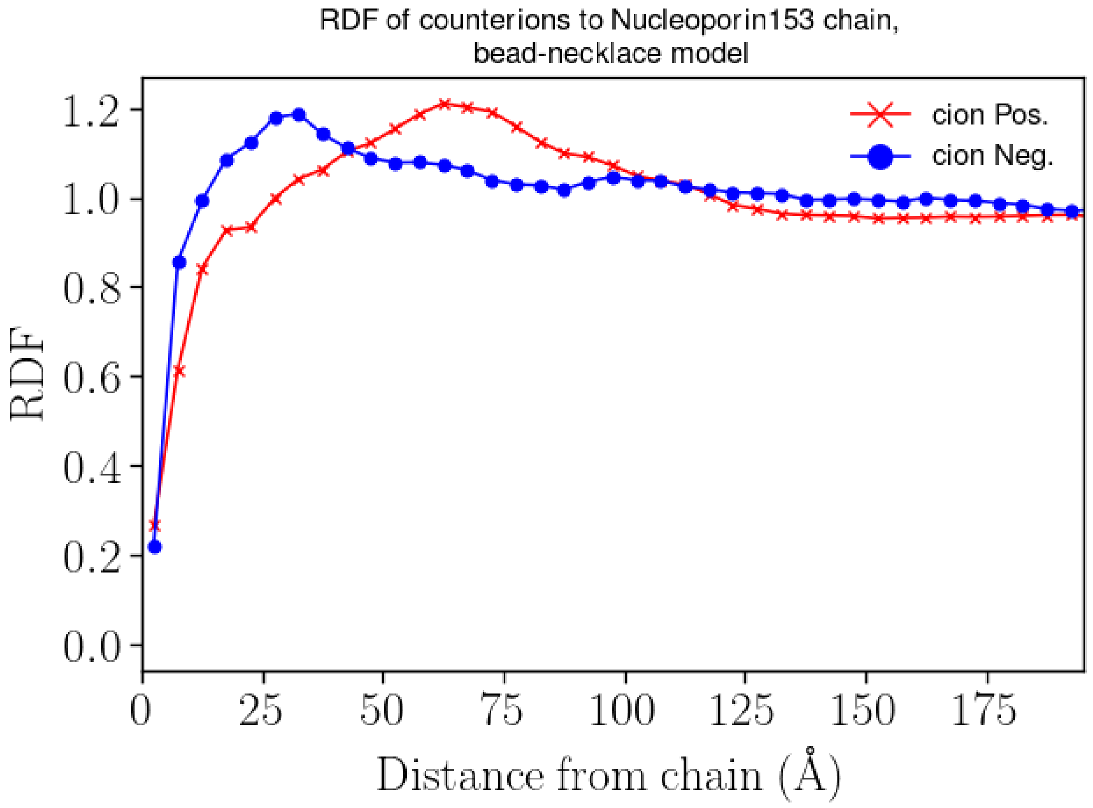

Figure S81: Radial distribution function (RDF) for the counter ions towards the protein chain, Nucleoporin153. Note that in this case, only one counter ion of each type is in the system, as well as Nucleoporin being electrostatically symmetric along the chain. The RDF for different counter ions should thus overlap, but due to the few counter ions in the system, the convergence for the RDF becomes poor.

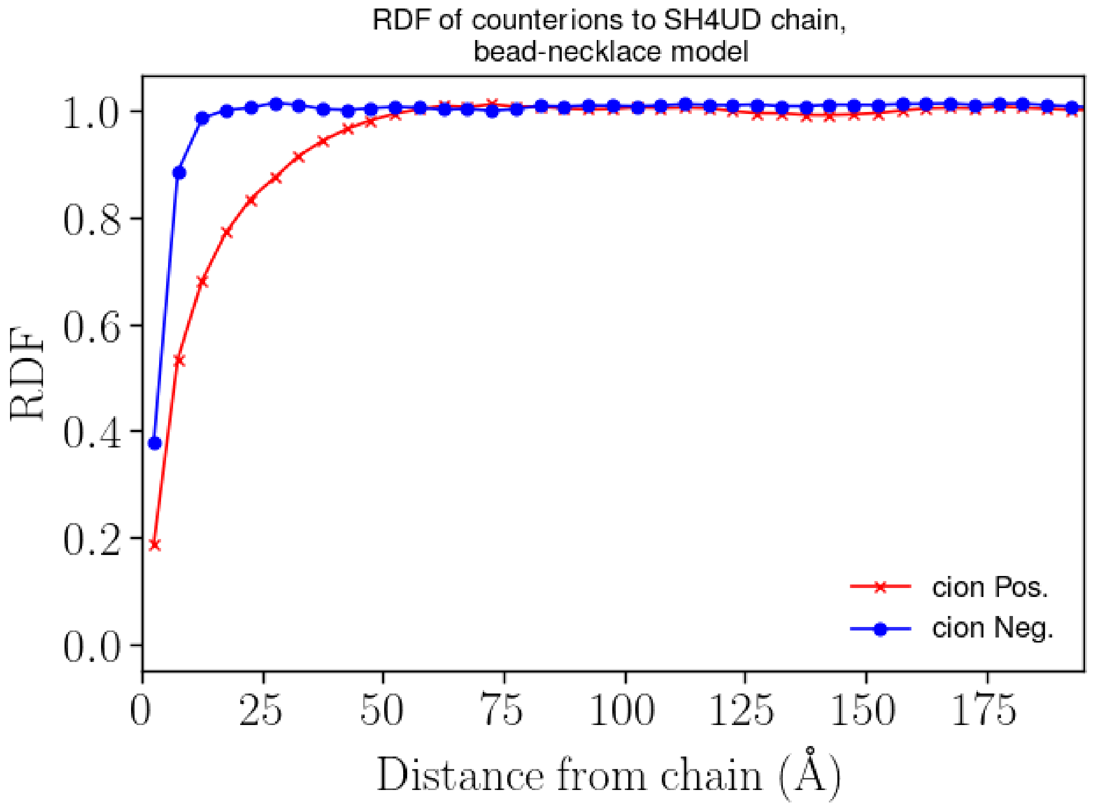

Figure S82: Radial distribution function (RDF) for the counter ions towards the protein chain, SH4UD.

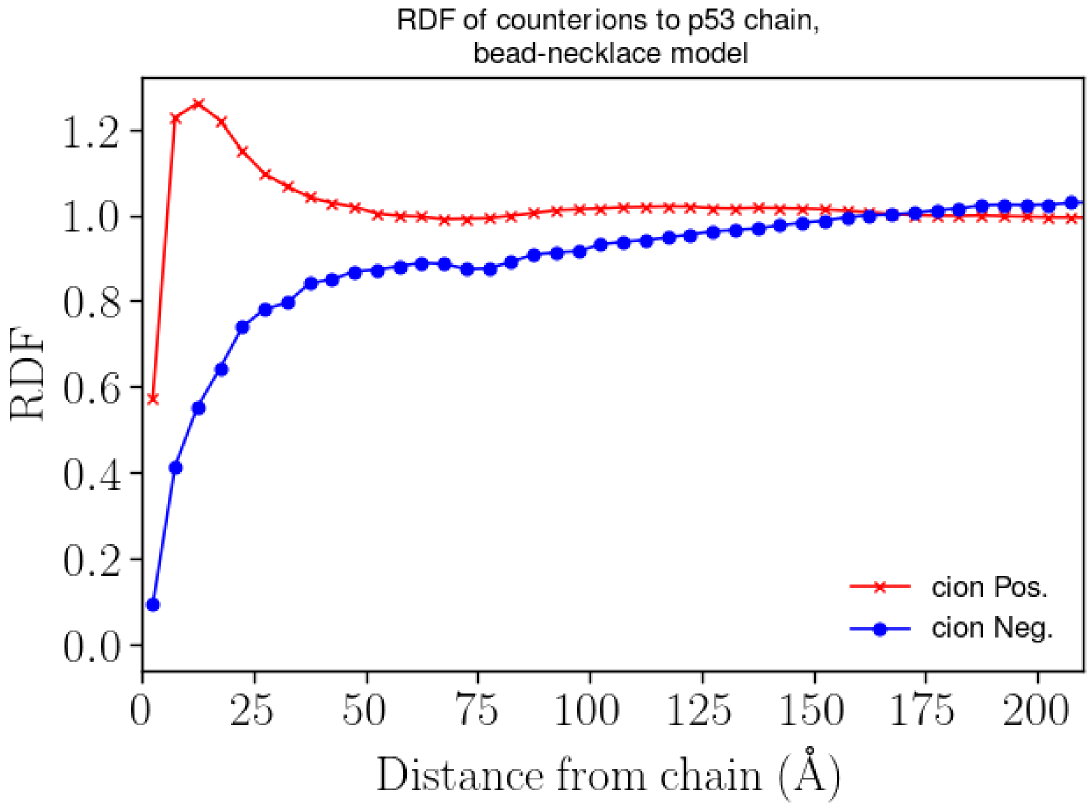

Figure S83: Radial distribution function (RDF) for the counter ions towards the protein chain, p53.

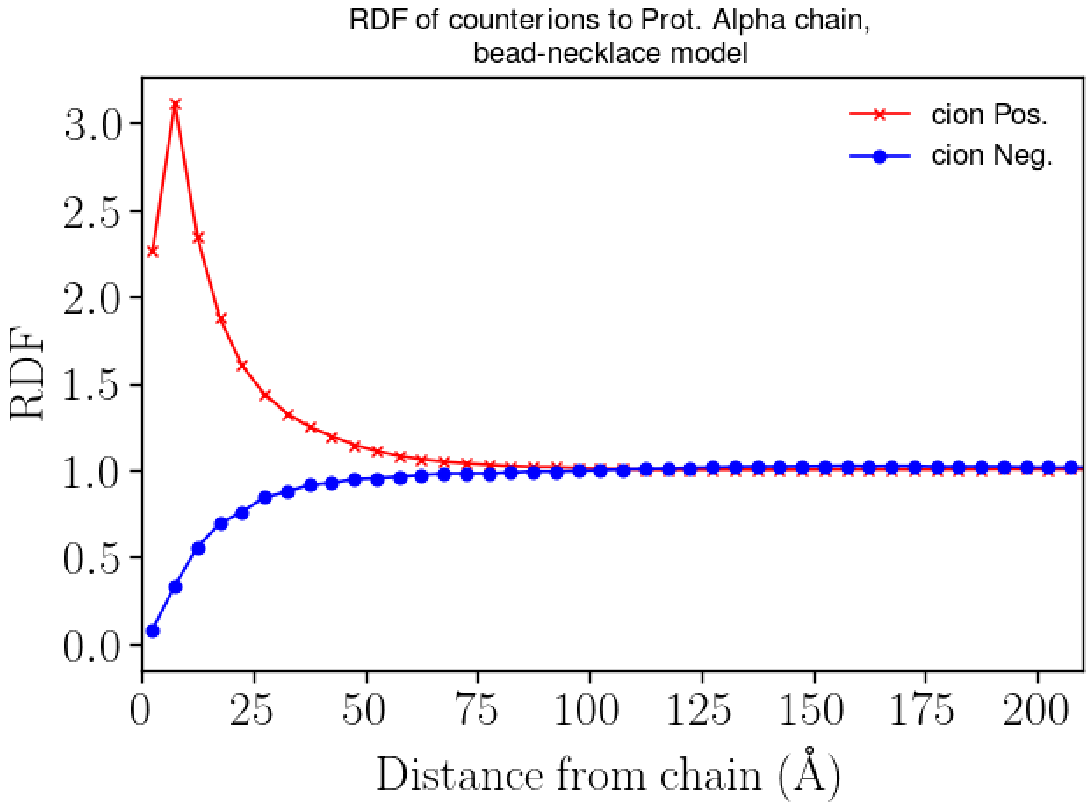

Figure S84: Radial distribution function (RDF) for the counter ions towards the protein chain, Prothymosin α.

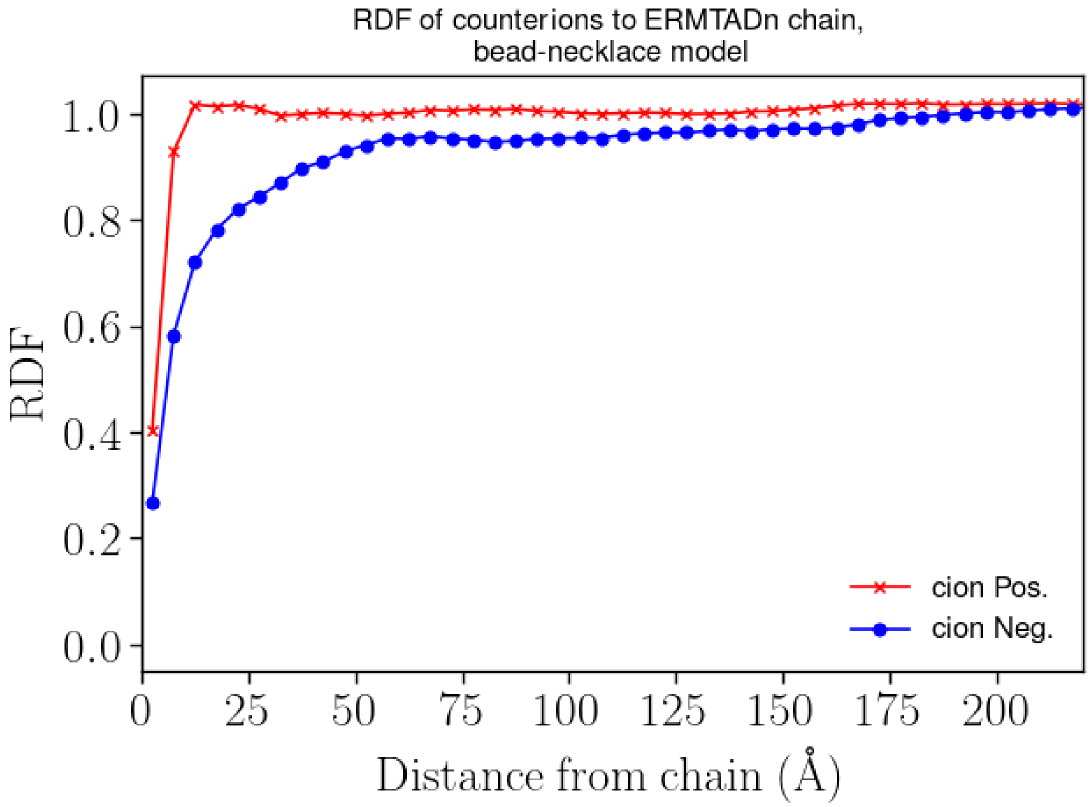

Figure S85: Radial distribution function (RDF) for the counter ions towards the protein chain, ERMTADn.

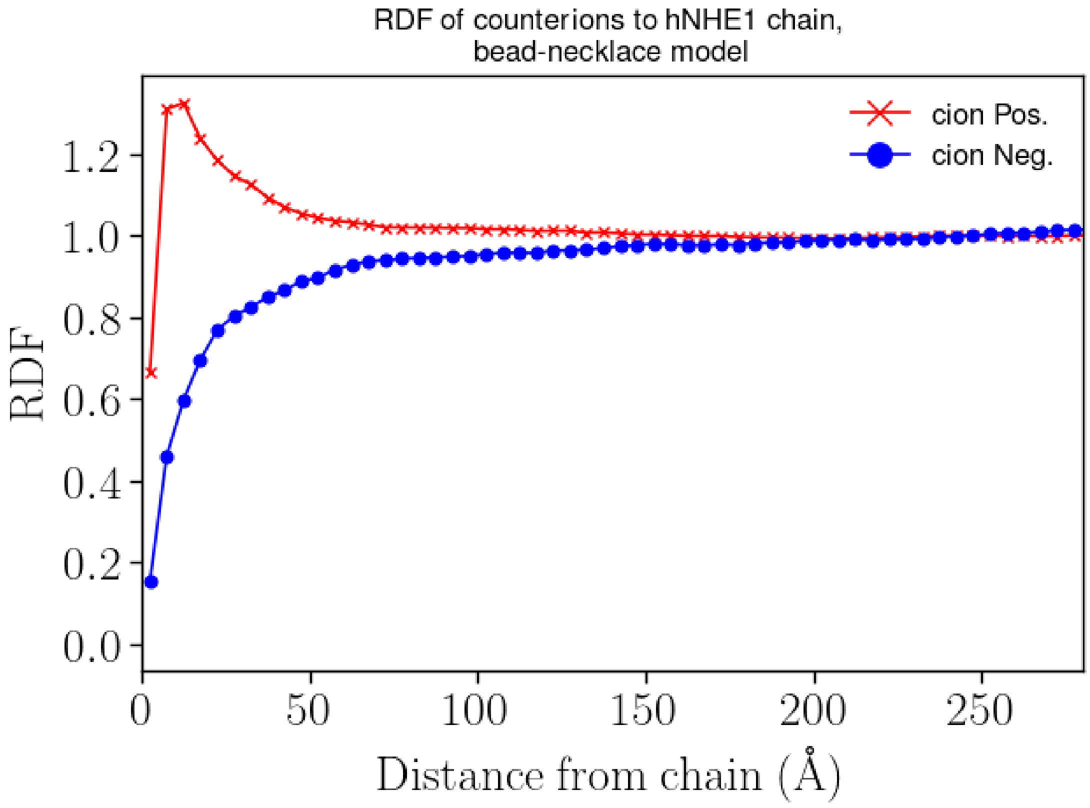

Figure S86: Radial distribution function (RDF) for the counter ions towards the protein chain, hNHE1.

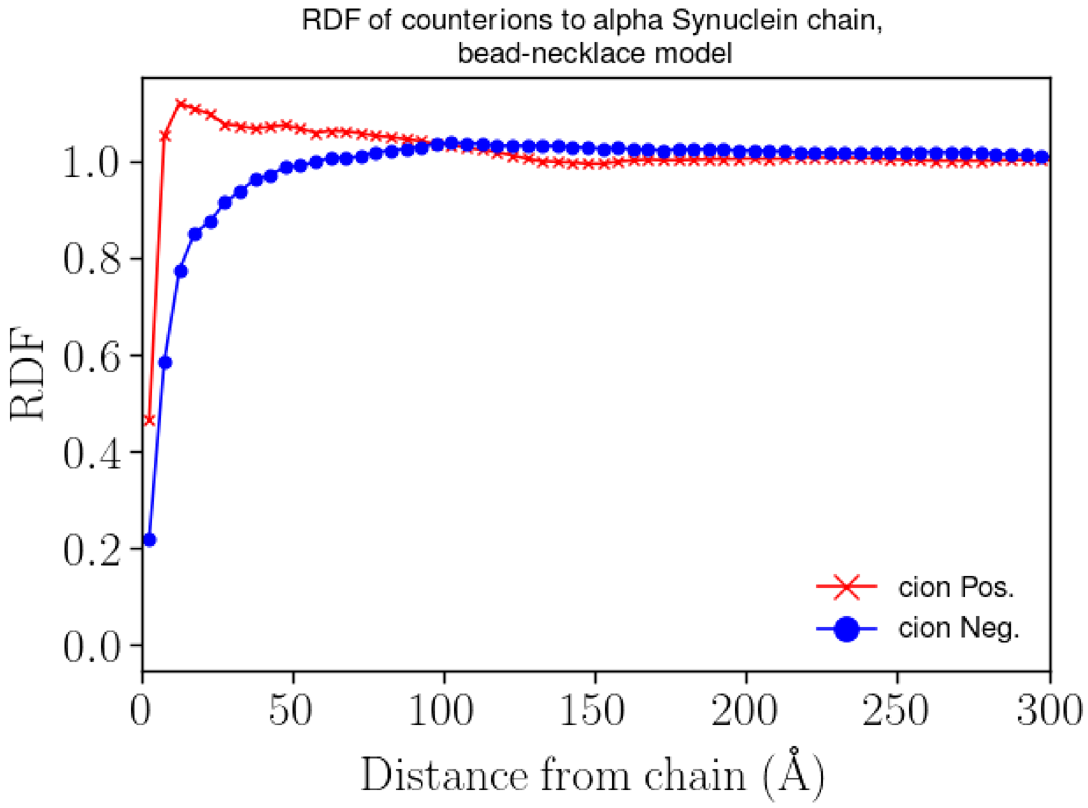

Figure S87: Radial distribution function (RDF) for the counter ions towards the protein chain, α synuclein.

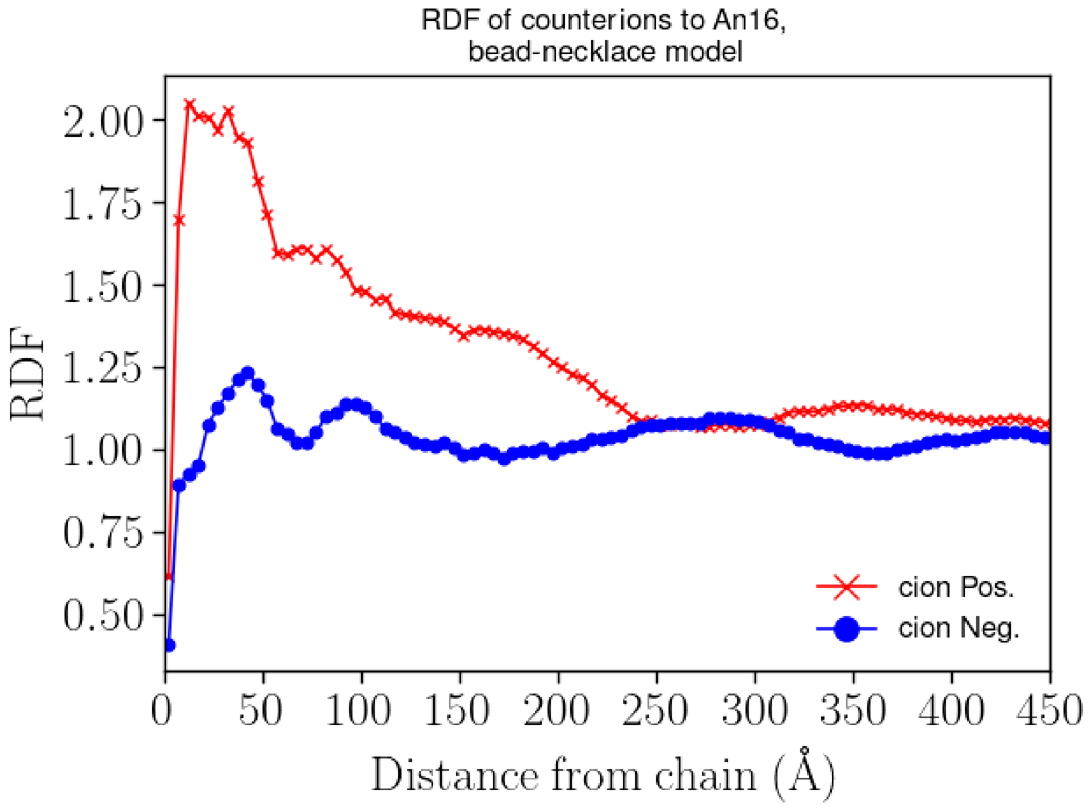

Figure S88: Radial distribution function (RDF) for the counter ions towards the protein chain, An16. Note that as in the case of Nucleoprin, only one counter ion of each type is in this system, as well as An16 being electrostatically symmetric along the chain. The RDF for different counter ions should thus overlap, but due to the few counter ions in the system, the convergence for the RDF becomes poor.

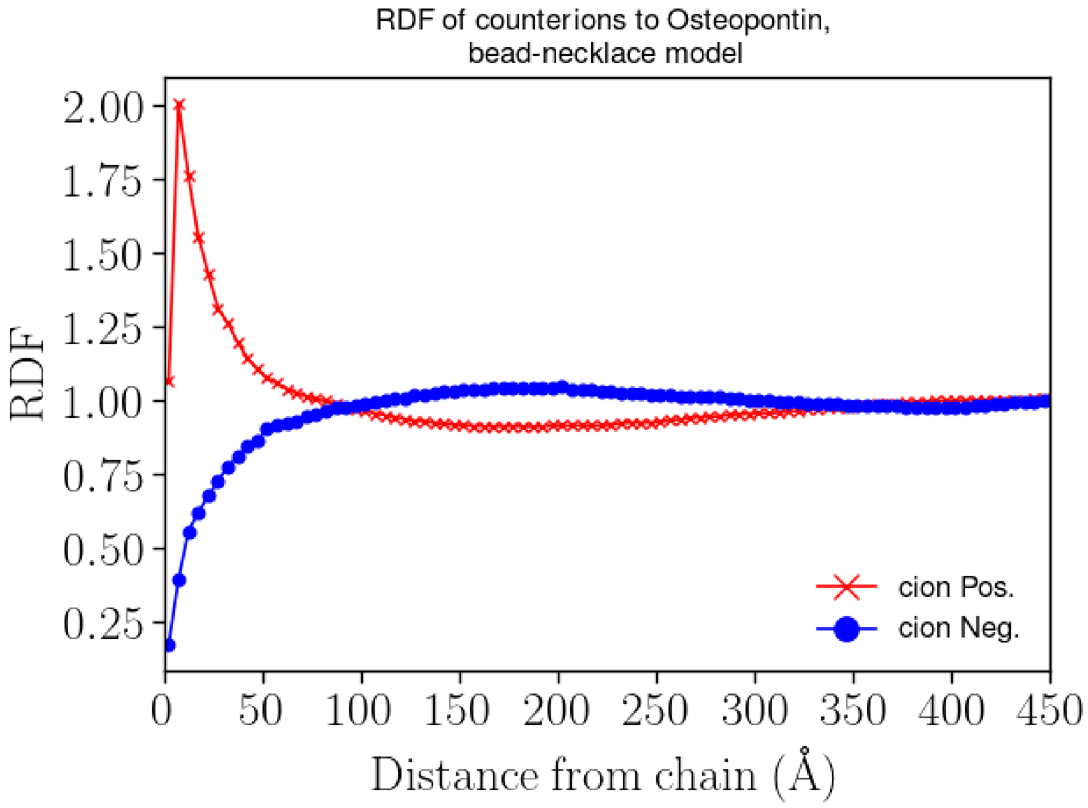

Figure S89: Radial distribution function (RDF) for the counter ions towards the protein chain, Osteopontin.

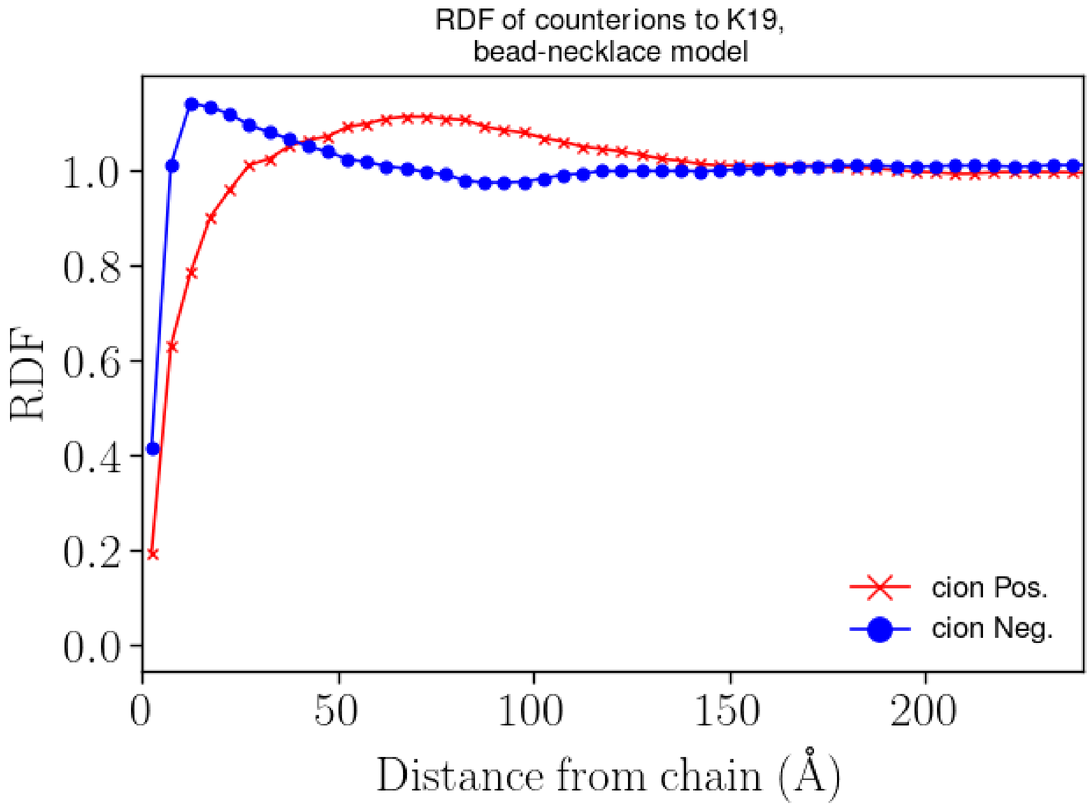

Figure S90: Radial distribution function (RDF) for the counter ions towards the protein chain, K19.

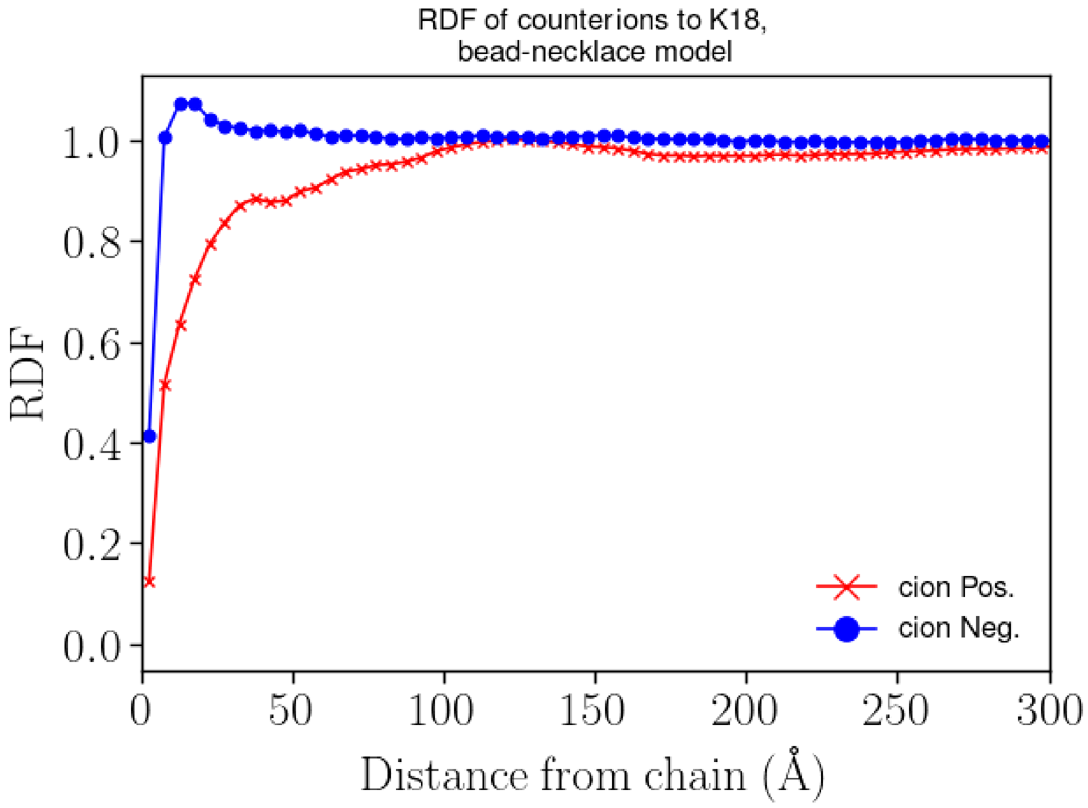

Figure S91: Radial distribution function (RDF) for the counter ions towards the protein chain, K18.

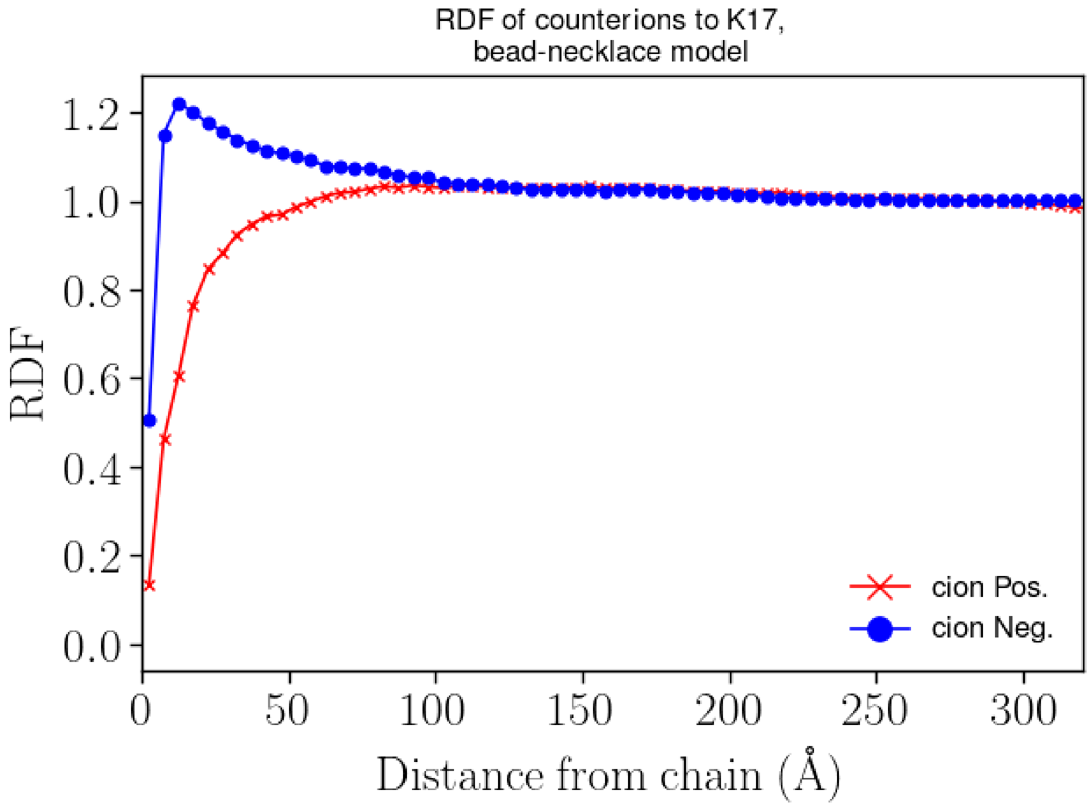

Figure S92: Radial distribution function (RDF) for the counter ions towards the protein chain, K17.

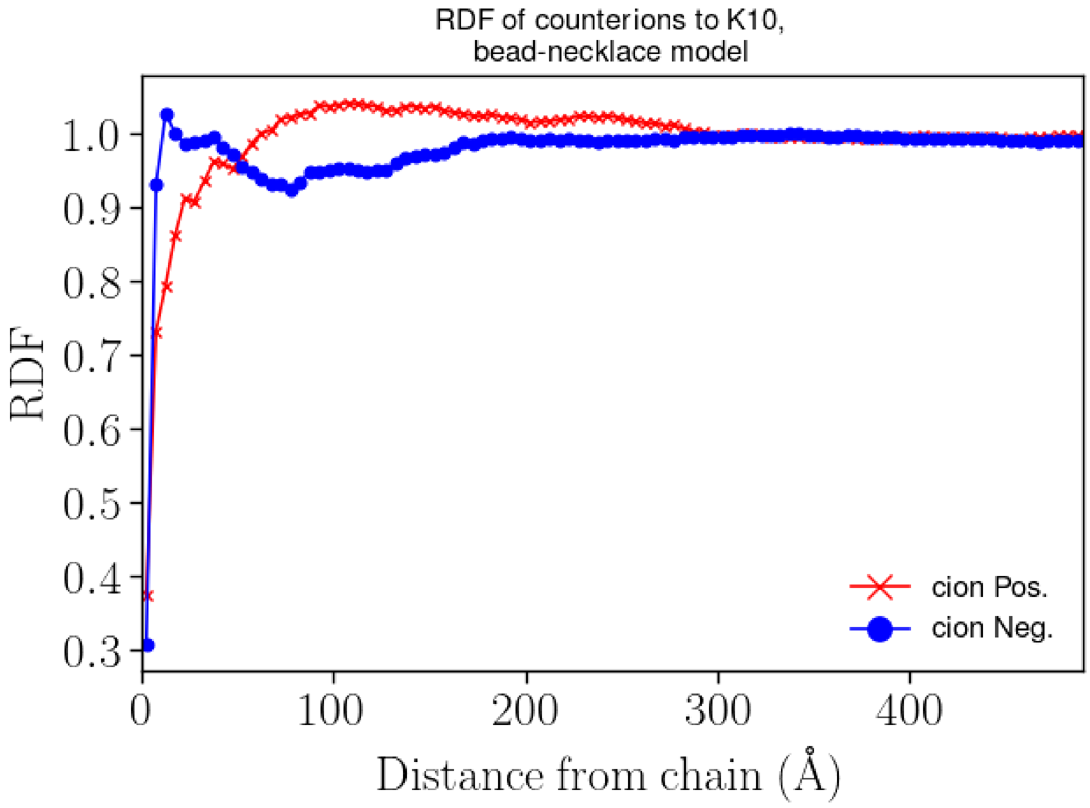

Figure S93: Radial distribution function (RDF) for the counter ions towards the protein chain, K10.

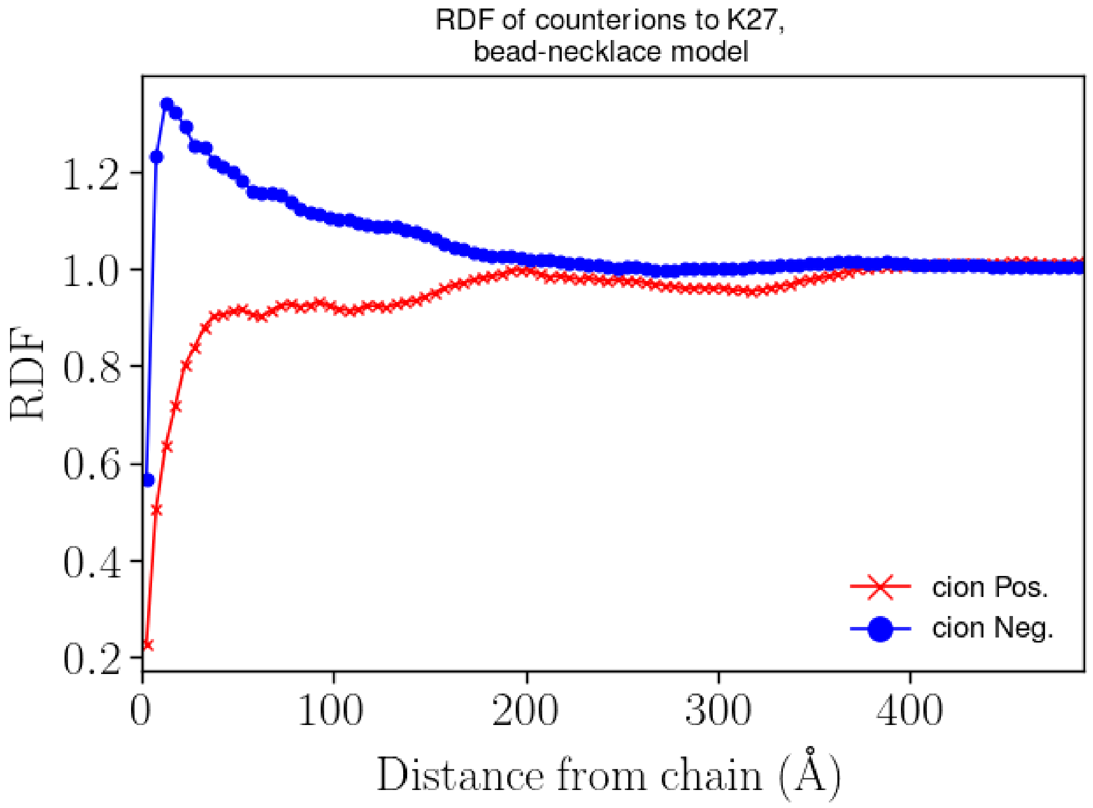

Figure S94: Radial distribution function (RDF) for the counter ions towards the protein chain, K27.

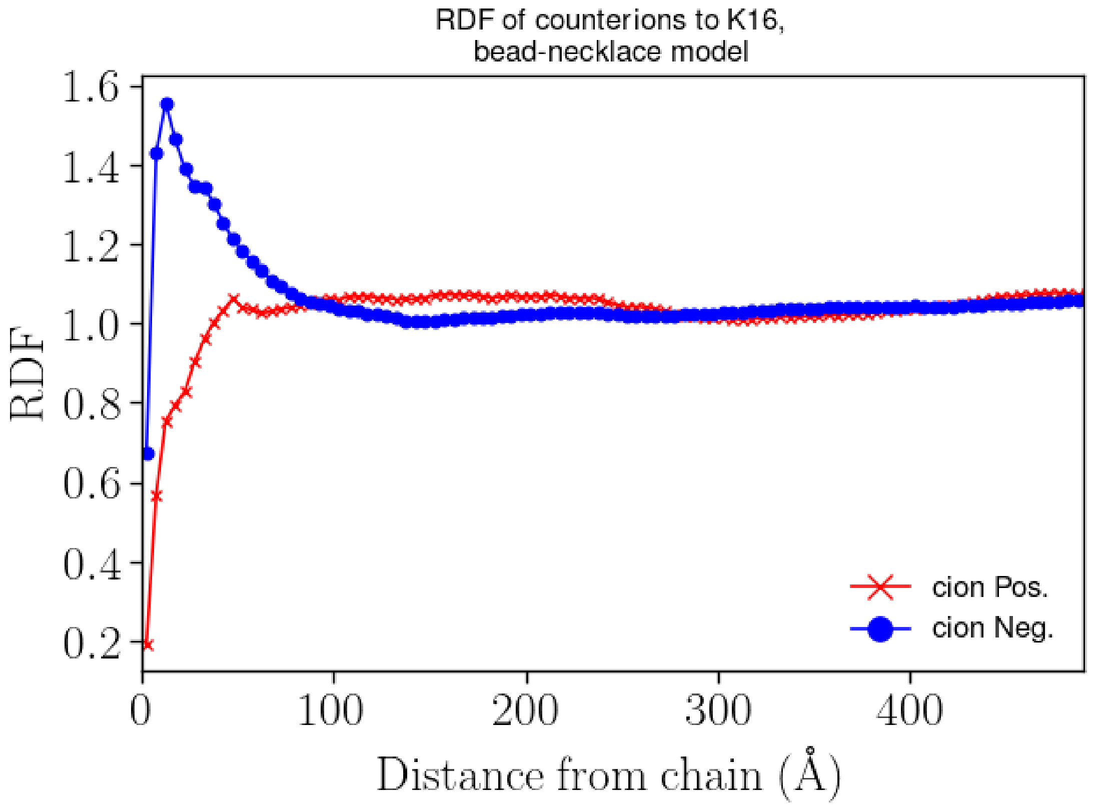

Figure S95: Radial distribution function (RDF) for the counter ions towards the protein chain, K16.

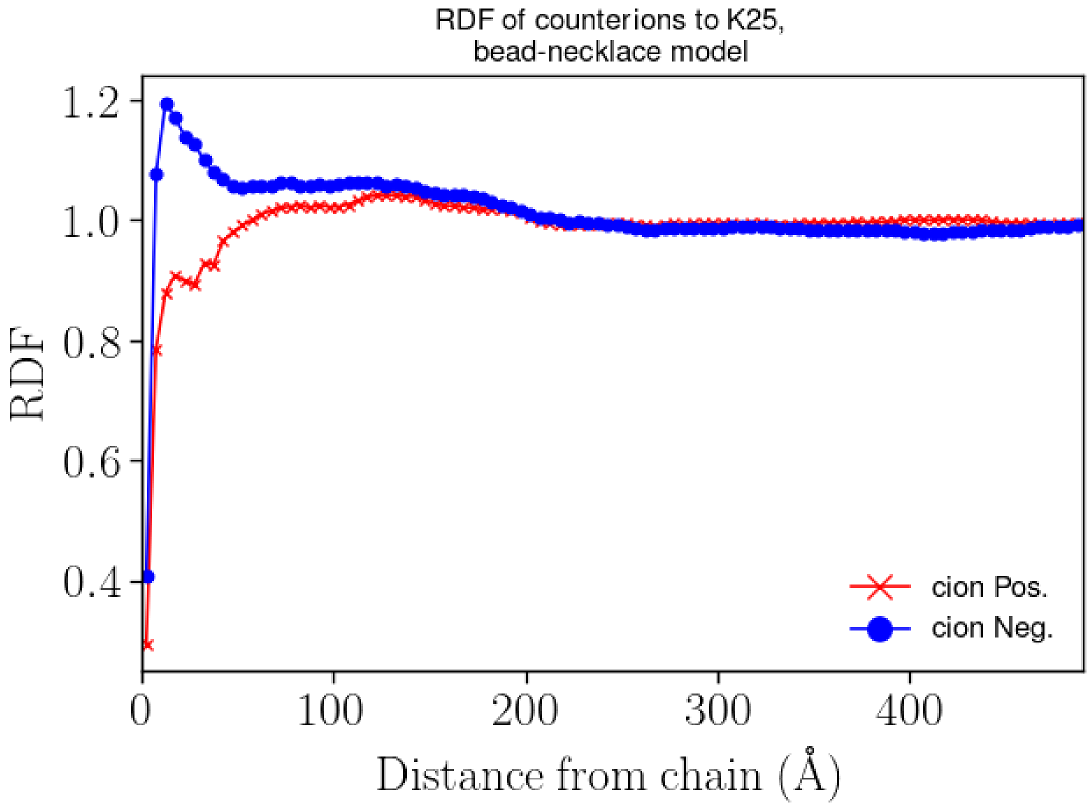

Figure S96: Radial distribution function (RDF) for the counter ions towards the protein chain, K25.

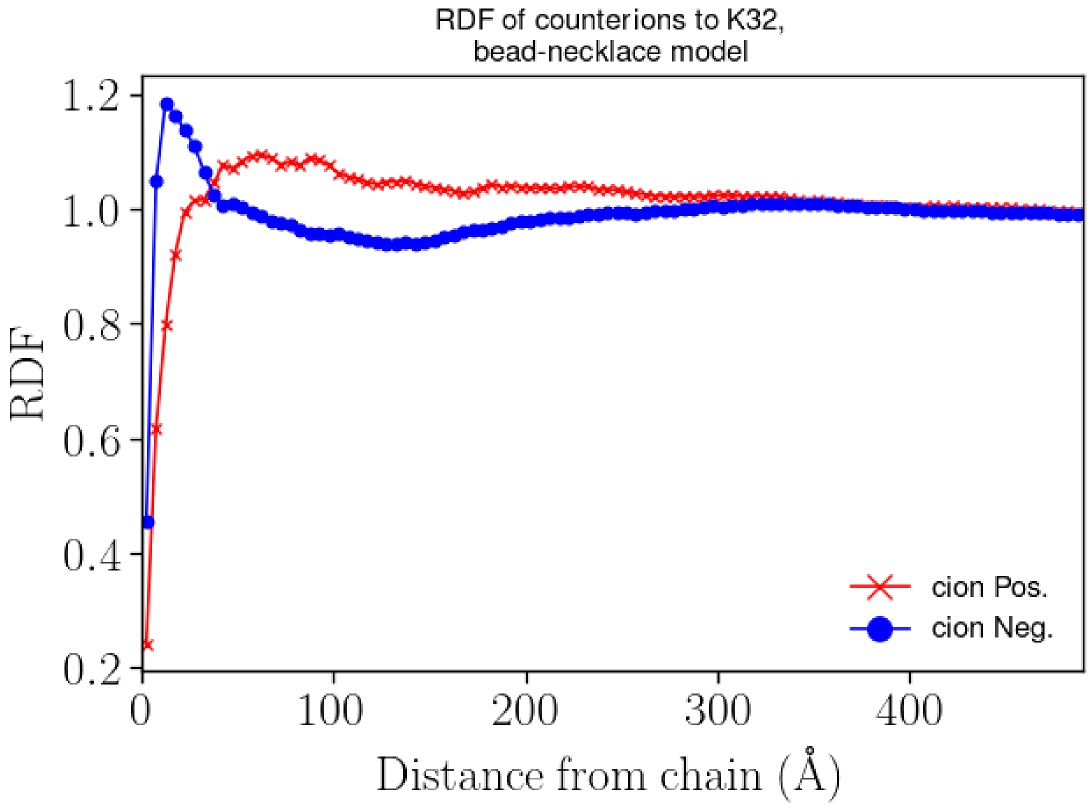

Figure S97: Radial distribution function (RDF) for the counter ions towards the protein chain, K32.

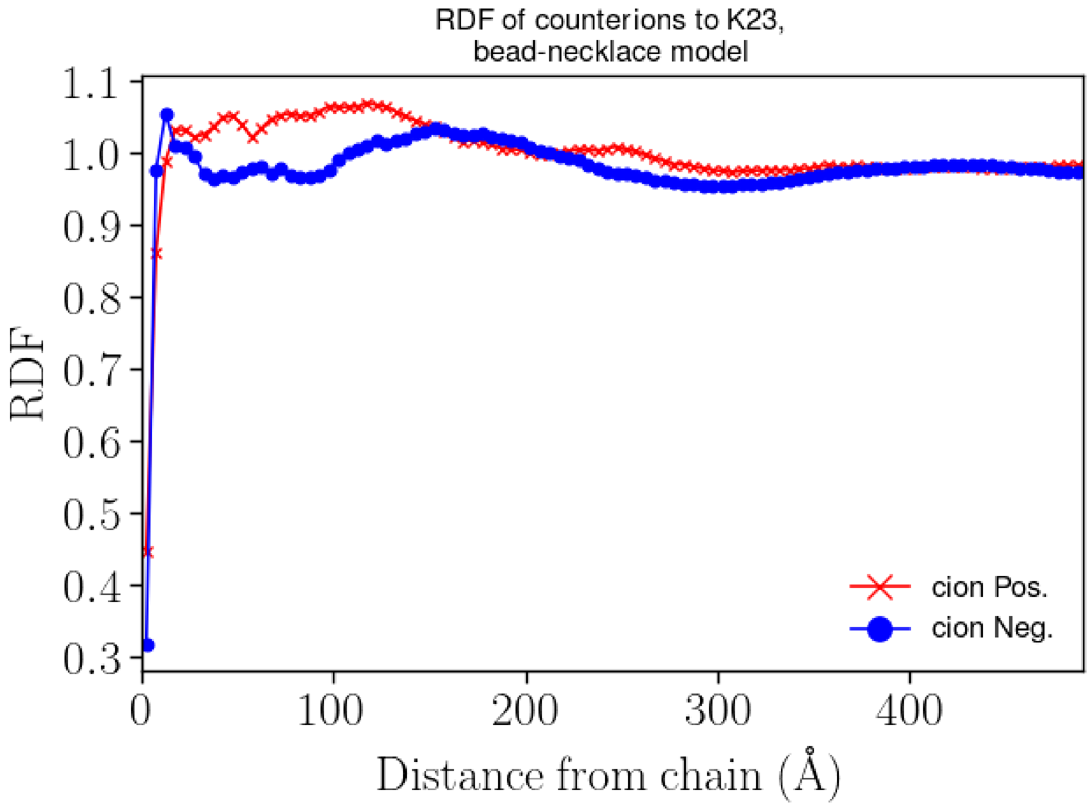

Figure S98: Radial distribution function (RDF) for the counter ions towards the protein chain, K23.

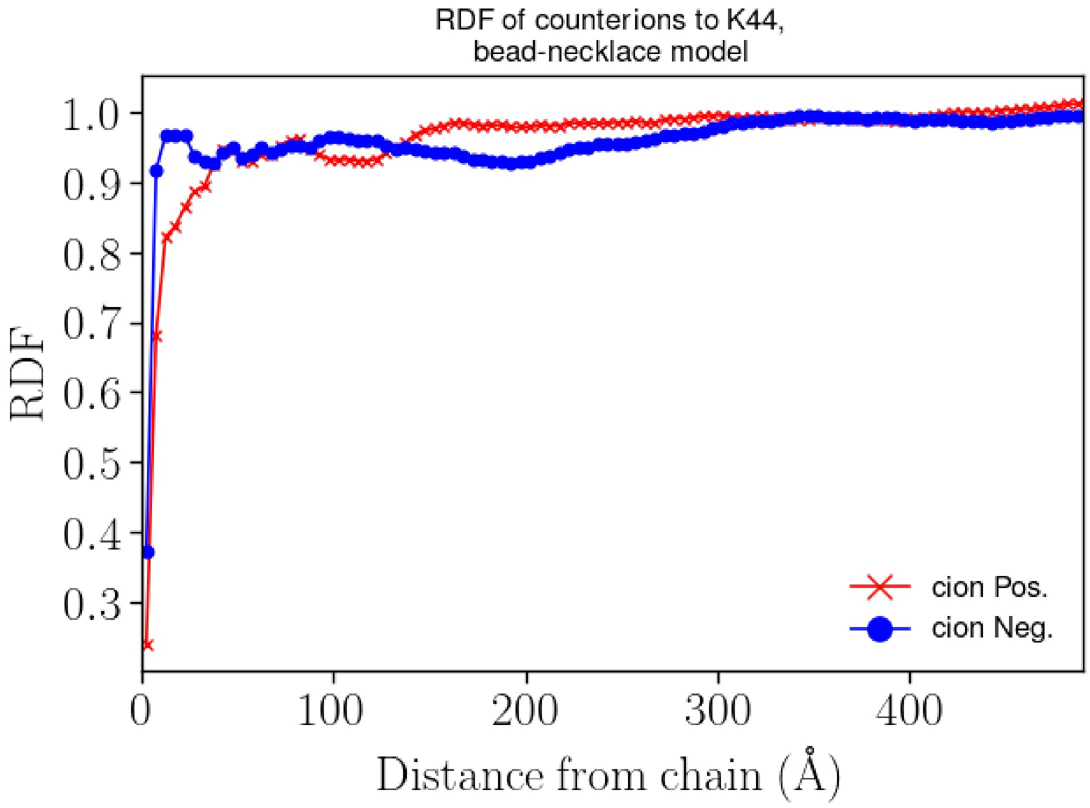

Figure S99: Radial distribution function (RDF) for the counter ions towards the protein chain, K44.

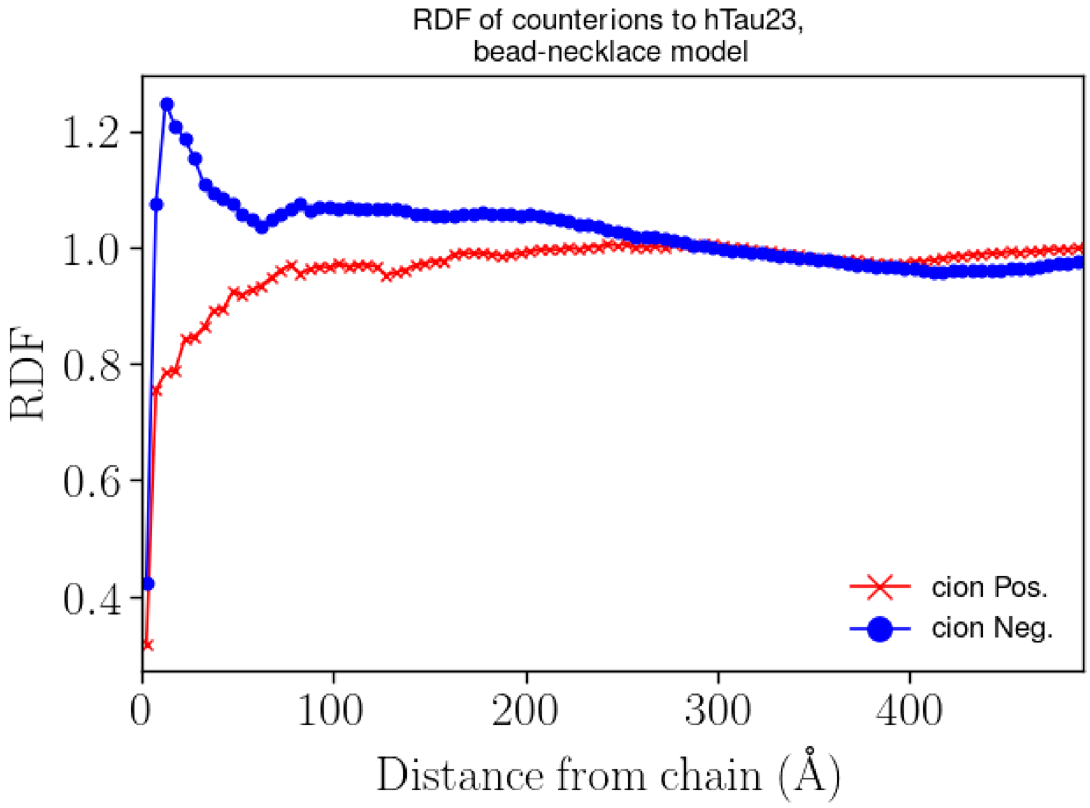

Figure S100: Radial distribution function (RDF) for the counter ions towards the protein chain, hTau23.

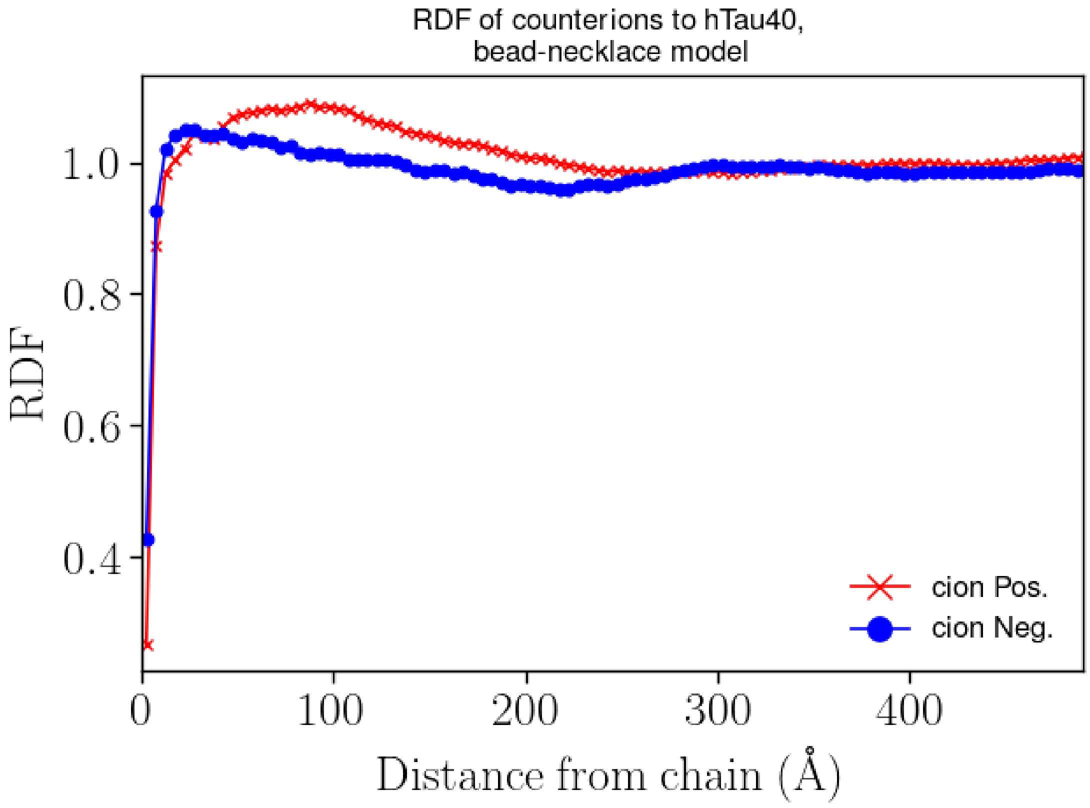

Figure S101: Radial distribution function (RDF) for the counter ions towards the protein chain, hTau40.

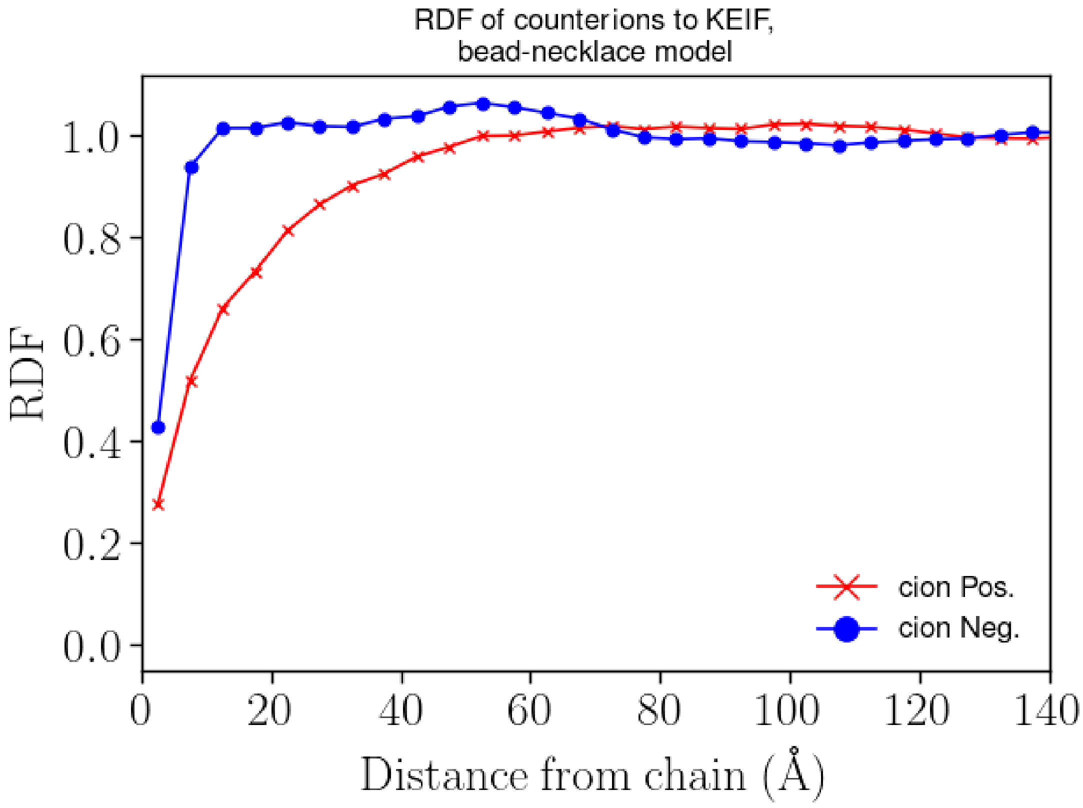

Figure S102: Radial distribution function (RDF) for the counter ions towards the protein chain, KEIF.

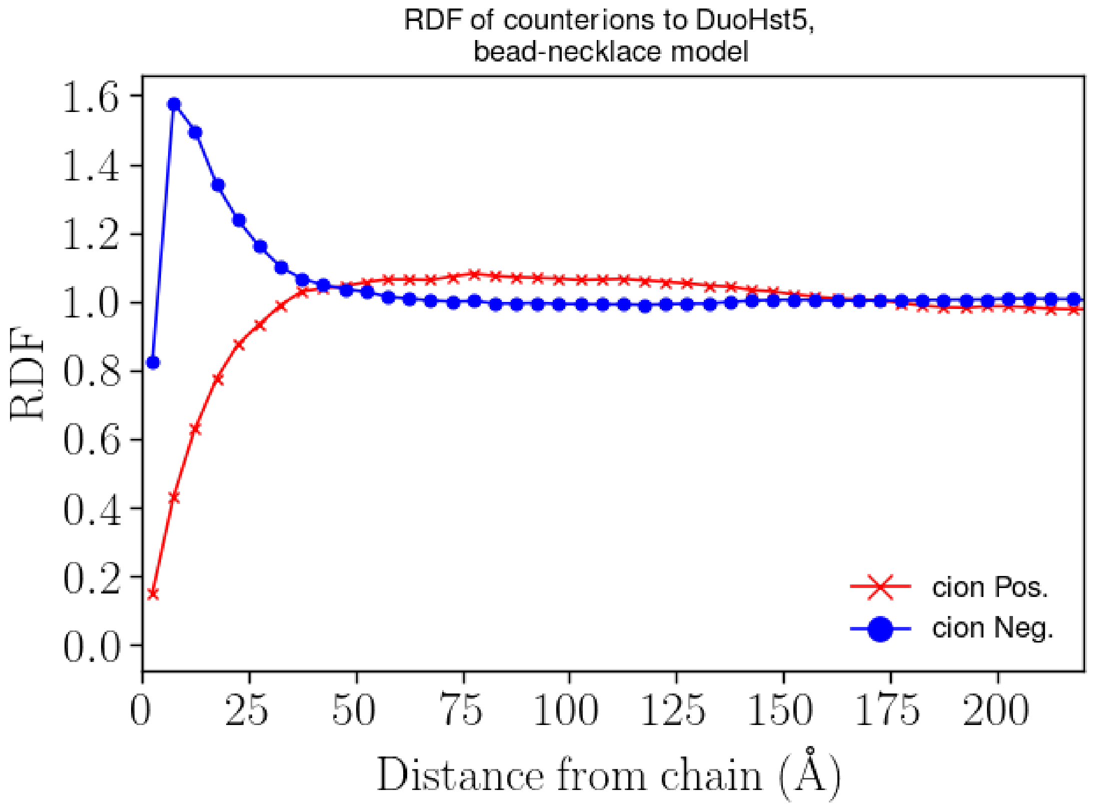

Figure S103: Radial distribution function (RDF) for the counter ions towards the protein chain, DuoHst5.

References

(1) Baul, U.; Chakraborty, D.; Mugnai, M. L.; Straub, J. E.; Thirumalai, D. Sequence Effects on Size, Shape, and Structural Heterogeneity in Intrinsically Disordered Proteins. *The Journal of Physical Chemistry B* **2019**, *123*, 3462–3474, PMID: 30913885.

(2) Arbesú, M.; Maffei, M.; Cordeiro, T. N.; Teixeira, J. M. C.; Pérez, Y.; Bernadó, P.; Roche, S.; Pons, M. The Unique Domain Forms a Fuzzy Intramolecular Complex in Src Family Kinases. *Structure* **2017**, *25*, 630–640.

(3) Uversky, V. N.; Gillespie, J. R.; Millett, I. S.; Khodyakova, A. V.; Vasilenko, R. N.; Vasiliev, A. M.; Rodionov, I. L.; Kozlovskaya, G. D.; Dolgikh, D. A.; Fink, A. L.; Doniach, S.; Permyakov, E. A.; Abramov, V. M. Zn2+-Mediated Structure Formation and

Compaction of the “Natively Unfolded” Human Prothymosin α. *Biochemical and Biophysical Research Communications* **2000**, *267*, 663–668.

(4) Lens, Z.; Dewitte, F.; Monté, D.; Baert, J.-L.; Bompard, C.; Sénéchal, M.; Van Lint, C.; de Launoit, Y.; Villeret, V.; Verger, A. Solution structure of the N-terminal transactivation domain of ERM modified by SUMO-1. *Biochemical and Biophysical Research Communications* **2010**, *399*, 104–110.
